# Multiscale learning of gene network-driven phenotypic dynamics of single cells

**DOI:** 10.1038/s44320-026-00220-x

**Published:** 2026-06-05

**Authors:** Dongyan Zhang, Jinan Li, Qing Nie, Xiaoqiang Sun

**Affiliations:** 1 School of Mathematics, Sun Yat-sen University, Guangzhou, 510275 China; 2 Department of Mathematics and Department of Developmental & Cell Biology, University of California Irvine, Irvine, CA 92697 USA; 3 Zhongshan School of Medicine, Sun Yat-sen University, Guangzhou, 510080 China

**Keywords:** Computational Biology

## Abstract

Understanding how gene regulatory networks (GRNs) dynamically orchestrate cell fate emergence remains a fundamental challenge. Here, we present GRNvelo, a computational framework that reconstructs multiscale cell fate dynamics by integrating GRNs with phenotypic dynamics from temporal single-cell RNA-seq data. GRNvelo establishes a biologically interpretable and mathematically rigorous multiscale model that couples GRN-driven single-cell velocity with nonlocal cell growth-mediated population dynamics. To operationalize this model, GRNvelo devises a two-phase cooperative optimization algorithm based on physics-informed neural networks (PINNs): TC-PINN for jointly inferring GRN velocity and latent time, and MP-PINN for refining GRN velocity within the context of cell population dynamics. In benchmark evaluations, GRNvelo demonstrates superior performance across two synthetic datasets and four real datasets, including branching development and diverse perturbation-response scenarios. Collectively, GRNvelo not only accurately infers GRN-driven cell fate dynamics but also predicts altered cell fates in response to diverse genetic perturbations, including dynamic and combined ones, thus establishing a new computational paradigm for predicting and modulating cell fate outcomes.

## Synopsis

GRNvelo, a multiscale computational framework, integrates physics-informed neural networks (PINNs) with gene regulatory networks (GRNs) to infer cell fate dynamics from time-series single-cell data, enabling prediction of cellular responses to complex perturbations.

- A multiscale model is established, bridging single-cell GRN dynamics to population-level phenotypic dynamics through rigorous mathematical analysis.
- A two-phase PINN-based algorithm is developed, incorporating a temporally-consistent network (TC-PINN) to infer GRN velocity with latent time and a multi-physics network (MP-PINN) to refine dynamics with population constraints.
- Simultaneous inference of cell velocity, latent time, trajectories, growth patterns, and GRNs is achieved within a unified framework, offering functional integration beyond existing methods.
- A new paradigm for complex perturbation predictions is provided, by which cellular responses to multi-gene knockouts and dynamic drug combinations can be predicted.

## Abstract (web summary)

GRNvelo, a multiscale computational framework, integrates physics-informed neural networks (PINNs) with gene regulatory networks (GRNs) to infer cell fate dynamics from time-series single-cell data, enabling prediction of cellular responses to complex perturbations.

## Graphical abstract

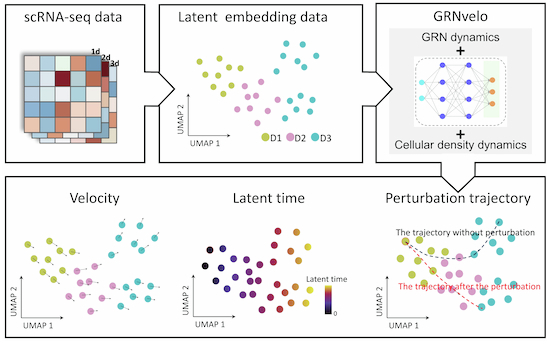

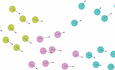

## Introduction

Cell fate determination represents one of the fundamental questions in life sciences (Fei et al, 2022; Wagner and Klein, 2020), spanning from developmental biology to regenerative medicine and disease pathology. The advent of single-cell sequencing technologies (Haque et al, 2017) has revolutionized our ability to examine cellular heterogeneity and dynamic transitions. Despite these advances, inferring continuous cell-state transition trajectories and their underlying regulators from static, snapshot data remains a major methodological challenge (Saelens et al, 2019).

Conventional pseudotime analysis methods for single-cell RNA sequencing (scRNA-seq) data, such as Monocle (Trapnell et al, 2014) and TSCAN (Ji and Ji, 2016), infer developmental trajectories by constructing minimum spanning trees or fitting smooth curves on data following dimensionality reduction. While these approaches have achieved some success in relatively simple linear differentiation processes like hematopoietic system development, they exhibit notable limitations when dealing with complex scenarios such as tumor heterogeneity (Tirosh et al, 2016). Specifically, these methods struggle to accurately capture reversible cell-state transitions, multi-branch differentiation paths, and nonlinear evolutionary relationships (Tritschler et al, 2019). To address these challenges, several dynamical system-based trajectory inference methods for time-series scRNA-seq data have emerged in recent years. TIGON (Sha et al, 2024) employs an unbalanced optimal transport method to simultaneously reconstruct dynamic cellular trajectories and model population growth across multiple snapshots. PI-SDE (Jiang and Wan, 2024) integrates neural stochastic differential equations (SDEs) with the Hamilton-Jacobi equation to reconstruct the underlying cellular potential energy landscape from time-series scRNA-seq data. MIOFlow (Huguet et al, 2022) integrates dynamical systems, manifold learning, and optimal transport to reconstruct continuous gene expression dynamics and decode them back into gene space, revealing expression time course patterns. TrajectoryNet (Tong et al, 2020) formulates trajectory inference as a manifold-constrained optimal transport problem, employing neural networks to learn continuous cell-state transition paths, demonstrating superior performance in mouse cortex and embryoid body datasets. PRESCIENT (Yeo et al, 2021) employs a generative modeling framework that conceptualizes cell differentiation as a diffusion process over an underlying potential landscape. Coupling neural networks to fit the potential function with estimates of cellular proliferation, it enables the prediction of cell fates and the simulation of trajectory changes under static, one-time perturbations applied only at the initial state.

Despite these advancements, current cell trajectory inference technologies still face critical limitations in accuracy and interpretability. The accuracy of trajectory reconstruction and pseudotime estimation remains contentious. Benchmark studies reveal that different algorithms (e.g., Slingshot (Street et al, 2018) vs. TSCAN (Ji and Ji, 2016)) may generate completely conflicting temporal ordering for the same dataset (Saelens et al, 2019), with such inconsistencies being most pronounced near branching points. The fundamental issue lies in the typically weak correlation between pseudotime variables and actual biological time (Campbell and Yau, 2016), compounded by their high susceptibility to cell cycle effects. Emerging latent time models (e.g., CellRank2 (Weiler et al, 2024)) attempt to improve accuracy by decoupling differentiation progression from cell cycle, but their dependence on prior marker genes raises concerns about applicability to rare cell types or pathological conditions. In terms of interpretability, most deep learning approaches (e.g., TrajectoryNet) lack the capability to identify key regulatory factors driving cell fate decisions, limiting their utility in mechanistic studies.

Moreover, fundamental challenges persist in single-cell trajectory inference, including cell growth dynamics modeling and identification of trajectory-driving gene regulatory networks (GRNs). Regarding cell growth modeling, most existing methods rely on the idealized assumption of constant cell numbers (Schiebinger et al, 2019), which contradicts the active processes of cell proliferation/apoptosis observed in real biological systems. As for the inference of driver GRNs, while mainstream trajectory analysis tools (e.g., PAGA (Wolf et al, 2019), Monocle2 (Qiu et al, 2017)) can delineate cell-state transition paths, they fail to identify the key regulatory genes driving these transitions. This limitation stems from conventional GRN inference tools (e.g., SCENIC (Aibar et al, 2017), PIDC (Chan et al, 2017)) generally lacking effective utilization of pseudotemporal information, causing dynamic regulatory relationships to be obscured by static correlations (Moerman et al, 2019). On the other hand, although several GRN inference methods (e.g., SCODE (Matsumoto et al, 2017) and Dictys (Wang et al, 2023)) represent an advance by utilizing temporal information, they often fail to bridge the gap between micro-scale regulations and macro-scale fate decisions. Their inferred networks remain descriptive of correlations over time rather than mechanistically explaining how evolving regulatory interactions drive trajectory formation, a problem compounded by the inherent inaccuracies in pseudotime estimation.

The coupling between single-cell gene regulatory dynamics and population-level processes lies at the core of cell fate determination. Synthetic biology has provided direct experimental evidence for this multiscale interdependence. A bistable synthetic gene circuit in yeast established that predicting population-level fitness under drug stress requires knowledge of both single-cell division rates and the nongenetic memory of distinct expression states (Nevozhay et al, 2012). Subsequent work further demonstrated that nonoptimal temperatures induce a bifurcation between growth arrest and stress resistance; capturing such bimodal population behavior necessitated models integrating population dynamics with single-cell growth and reaction kinetics (Charlebois et al, 2018). Together, these findings highlight the necessity of a computational framework capable of bridging molecular regulatory mechanisms with population phenotypes, a capability largely absent from current approaches to predicting cell fate dynamics.

To address the above limitations and challenges, we introduce GRNvelo, a computational framework designed to infer multiscale cell fate dynamics (MCFD) from time-series single-cell transcriptomic data. GRNvelo integrates gene regulatory mechanisms with cell population dynamics through a multiscale model optimized using physics-informed neural networks (PINNs) (Roquemen-Echeverri and Mosquera-Lopez, 2025). The core innovations of our approach are threefold. First, at the gene level, it simultaneously infers gene–gene interaction dynamics and cellular latent time under mutual constraints, achieving a novel integration of temporal and kinetic information. Second, at the population level, the model incorporates phenotypic (epi)mutations and environment-constrained growth to further refine the dynamic behavior of cell fates. Third, the framework supports multitasking, concurrently outputting diverse key MCFD information, including cell velocity, latent time, trajectory, growth patterns, and GRNs. To assess the accuracy and utility of GRNvelo, we systematically evaluated its performance on two simulated bifurcation datasets and four real datasets spanning representative biological processes, including developmental differentiation, infectious immune responses, and tumor drug resistance. Furthermore, we demonstrate the ability of GRNvelo to predict cell fate dynamics under complex perturbation scenarios, such as dynamic drug combination schedules and multi-gene knockout (KO) experiments.

This study offers three main contributions. Theoretically, we build the first multiscale model by connecting the GRN-based state transition velocity of single cells to cell population-level phenotypic dynamics via rigorous mathematical analysis. Methodologically, we devise a two-phase cooperative optimization algorithm based on physics-informed neural networks (PINNs): a temporally-consistent network (TC-PINN) for jointly inferring GRN velocity and latent time, and a multi-physics network (MP-PINN) that refines GRN velocity within the context of cell population dynamics. Practically, we develop a new versatile tool for identifying key driver genes of fate transitions and predicting dynamic cellular responses to diverse genetic perturbations, including dynamic and combined ones.

## Results

### Overview of GRNvelo

**Figure 1:**
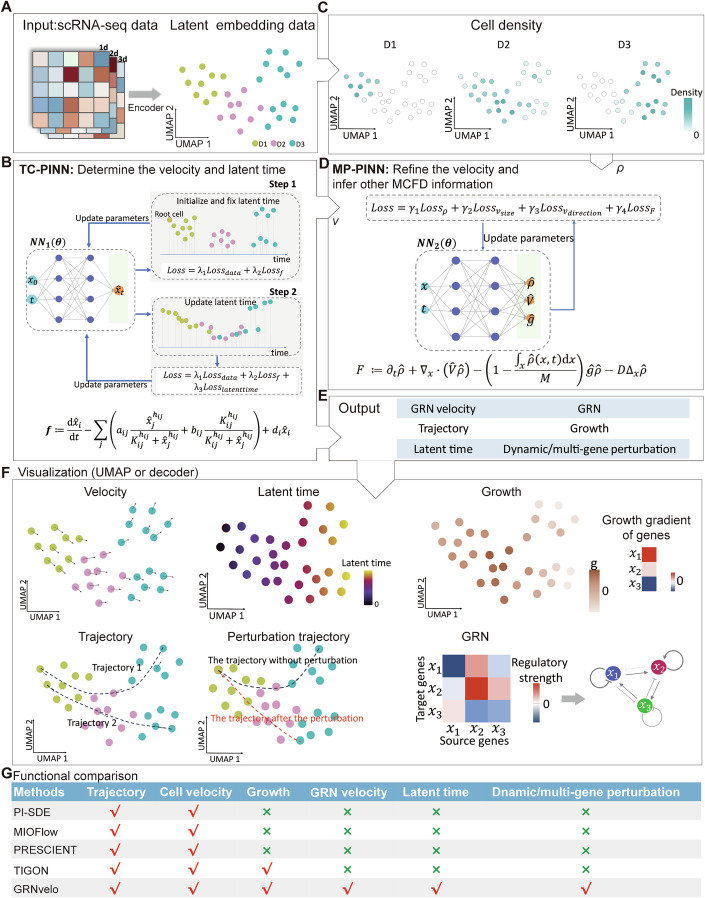
GRNvelo framework overview. (**A**) Input: temporal scRNA-seq data (high-dim) and latent embeddings (low-dim). (**B**) GRN Velocity inference via TC-PINN. (**C**) Cell density estimation by GMM. (**D**) Cell fate dynamics inference via MP-PINN. (**E**) Outputs: Velocity, trajectory, growth, GRN, latent time and perturbation trajectory. (**F**) Visualization of outputs. (**G**) Functional comparison of GRNvelo against other cellular dynamics inference methods.

We present GRNvelo, a multiscale computational framework that infers GRN-driven cell-state transition velocity by modeling biological processes from gene regulation to cell population dynamics (Fig. 1). The framework builds upon a multiscale kinetic model that bridges microscopic gene regulation mechanisms of single cells described by ordinary differential equations (ODEs) to macroscopic population dynamics described by partial differential equation (PDE). Through rigorous mathematical analysis, we established a connection between the GRN-based RNA velocity derived from the ODEs model and the cell population-level phenotypic transition velocity described by the PDE model.

GRNvelo takes discrete-time single-cell gene expression data as input (Fig. 1A) and operates through two functionally coupled submodules, TC-PINN (Fig. 1B) and MP-PINN (Fig. 1D), enabling comprehensive analysis of multiscale cell fate dynamics. The first module, TC-PINN, integrates the nonlinear dynamic equations of gene networks with neural networks. It innovatively incorporates discrete time points as prior information to enforce temporal consistency constraints, ensuring that the latent time inferred by the neural network maintains biological plausibility along the time dimension, while learning a continuous dynamic process from discrete and highly sparse time-series data. This module effectively mitigates the overfitting problem commonly encountered with traditional methods when data are scarce, providing a robust description of gene-scale dynamics and laying the foundation for accurately quantifying phenotypic dynamics. In this study, latent time refers to the continuous temporal variable learned by GRNvelo through the TC‑PINN module, which enforces consistency with experimental time points and gene regulatory ODE constraints. The latent time is normalized to [0,1] for interpretability, and it preserves an affine relationship with the true experimental time, thereby retaining absolute temporal information. This mechanistic model-based latent time distinguishes it from conventional expression similarity-based pseudotime that provides only a relative sequence of cell ordering without alignment to real time.

Building on this, the second module, MP-PINN, models population-level dynamics encompassing phenotypic transitions and cell growth. It combines cell density dynamics with neural networks to further refine the GRN velocity derived from TC-PINN, ensuring consistency with the cell population dynamics. By using the magnitude and direction of the GRN velocity provided by TC-PINN as a constrained reference, and simultaneously integrating density constraints governing cell behavior into a joint optimization process (Fig. 1C), it deeply fuses gene expression information with population dynamics equations controlling cell fate transitions. This approach not only accurately predicts cell-state transition rates, trajectories, and population growth dynamics, but also infers the underlying gene networks driving these phenotypes (Fig. 1E,F).

Through this multiscale integration strategy, GRNvelo establishes for the first time a unified modeling and inference framework—driven by both data and physical models—that spans from single-cell gene regulatory mechanisms to the time course of phenotypic structures at the population level. It comprehensively reveals the dynamic relationships from genes to phenotypes in a top-down manner. Furthermore, GRNvelo can predict cellular response trajectories under perturbed conditions, significantly enhancing the generalizability and interpretability of predictions in complex biological systems.

To address the computational challenges posed by the high-dimensional nature of scRNA-seq data, we employed autoencoders (AEs) as a reversible dimensionality reduction technique (Fig. 1A). This approach enables us to perform computations in a reduced-dimensional latent space while preserving the essential features of the original data through its encoder-decoder architecture. In the present method, the original high-dimensional gene expression data are mapped by an encoder into a low-dimensional latent space, wherein subsequent computations are performed. Following model training, we visualized the cellular distributions using UMAP projections and subsequently mapped the results back to the original high-dimensional space to conduct gene perturbation analyses and other investigations. This bidirectional dimensional transformation strategy effectively balances computational tractability with biological interpretability, allowing for efficient model training without compromising our ability to analyze high-dimensional gene regulatory patterns.

We summarized the characteristics of GRNvelo in comparison with other methods for inferring cellular dynamics from time-series scRNA-seq data (Fig. 1G), which revealed distinct functional specializations. Existing methods, such as MIOFlow, PI-SDE, and PRESCIENT, primarily focus on predicting cellular velocity and trajectories; PRESCIENT can also simulate cellular responses under perturbed conditions. TIGON represents an advancement, as it infers not only cellular velocity and trajectories but also GRNs and cellular growth rates. Remarkably, GRNvelo achieves an unparalleled level of functional integration. Unlike other methods that derive velocity from cell population models, GRNvelo infers “GRN velocity” for each cell based on the intrinsic gene regulatory network. This unique foundation makes GRNvelo the only framework capable of simultaneously inferring cell-specific latent time and simulating trajectories under perturbation. This integrated capability allows GRNvelo to provide a holistic and dynamic view of cell fate decisions, capturing intrinsic temporal progression and system-level responses to external or genetic perturbations. Consequently, GRNvelo offers an unprecedented multiscale perspective for dissecting gene regulatory network-driven phenotypic dynamics in single cells.

### Validation on synthetic data

To evaluate GRNvelo on the simplest regulatory motif, we first simulated a single-gene positive autoregulation system using the Gillespie algorithm (Gillespie, 1977; Matthew et al, 2023), which captures the intrinsic stochasticity of biochemical reactions (Appendix Fig. S1A). From the simulated trajectories, we sampled cells at five discrete time points for model training and validation (Appendix Fig. S1B). GRNvelo successfully reconstructed the gene expression dynamics (Appendix Fig. S1C) and accurately predicted cellular trajectories (Appendix Fig. S1D), demonstrating its capability to infer cell fate dynamics from data generated under a fundamentally different stochastic modeling framework. Building on this validation, we next evaluated GRNvelo on more complex synthetic networks.

**Figure 2:**
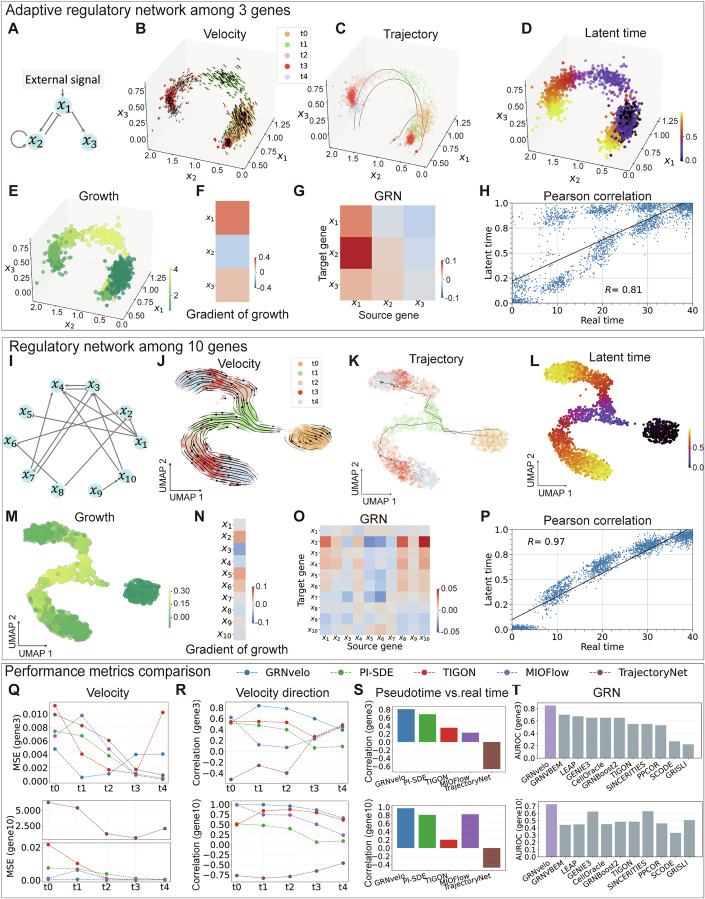
Validation and comparison of GRNvelo on synthetic datasets generated from simulated 3-gene and 10-gene networks. (**A**) Topology of the 3-gene network. (**B**–**H**) Performance of GRNvelo on the 3-gene network data in terms of (**B**) velocity field estimation, (**C**) trajectory reconstruction, (**D**) latent time estimation, (**E**) growth dynamics, (**F**) gene gradients of growth, (**G**) GRN reconstruction, and (**H**) correlation between latent time and real time. Pearson’s correlation, *r* =  0.81, *n* = 2778, *p* < 0.0001. (**I**) Topology of the 10-gene network. (**J**–**P**) Performance of GRNvelo on the 10-gene network data in terms of (**J**) velocity field, (**K**) trajectory reconstruction, (**L**) latent time estimation, (**M**) growth dynamics, (**N**) gene gradients of growth, (**O**) GRN reconstruction, and (**P**) correlation between latent time and real time. Pearson’s correlation, *r* = 0.97, *n* = 2851, *p* < 0.0001. (**Q**–**T**) Comparative metrics: (**Q**) MSE of velocity magnitudes, (**R**) directional correlation of velocities, (**S**) Pearson correlation of pseudotime vs. real time, and (**T**) GRN inference AUROC. Source data are available online for this figure.

To further evaluate GRNvelo on more complex regulatory networks, we next conducted systematic evaluations using two synthetic datasets generated from networks of different scales (3-gene and 10-gene systems) (Fig. 2). For the 3-gene network (Fig. 2A), we implemented an adaptive circuit featuring a negative feedback loop with a buffering node (Ma et al, 2009), which simulated two distinct cellular trajectories. From each trajectory, we sampled cells at five time points for model training. Both trajectories incorporated external signal inputs but exhibited fundamentally different regulatory behaviors. In the first trajectory, gene *x*_2_ played a dominant role through both autoregulation and inhibition of *x*_1_, generating highly nonlinear state transitions. Specifically: (i) *x*_1_ expression initially increased then stabilized under negative feedback regulation of *x*_2_; (ii) *x*_2_, activated by both *x*_1_ and itself, peaked before maximum expression of *x*_1_ then declined to a steady-state; and (iii) *x*_3_, promoted by *x*_1_, followed similar biphasic dynamics (Appendix Fig. S2A). The second trajectory presented simpler linear dynamics due to weakened *x*_2_ autoregulation and *x*_1_ inhibition. Here, *x*_1_ gradually decreased through self-degradation, while *x*_2_ and *x*_3_ followed this declining trend through *x*_1_-mediated activation (Appendix Fig. S2B). Notably, we assumed that *x*_1_ upregulation triggered cell division and subsequent population expansion in this linear regime (see details in the Methods section). Additionally, to evaluate robustness under higher noise, we generated synthetic datasets from the 3-gene network with noise standard deviations ranging from 0.02 to 0.10. Across all noise levels, the velocity magnitude MSE remained below 0.005. GRNvelo maintained stable performance for noise levels ≤0.06, with velocity direction correlation >0.5 and latent time correlation >0.6, while performance slightly declined at higher noise levels (Appendix Fig. S3).

For the 10-gene network, we adopted a gene regulatory network from DREAM4 (Greenfield et al, 2010), in which the core node *x*_3_ formed two negative feedback loops with *x*_4_ and *x*_7_, respectively, and the remaining nodes further expanded the regulatory hierarchy. The simulation generated two distinct trajectories of cellular dynamics under this network, with cells sampled at five time points for model training. Both groups shared similar initial gene expression values, with identical inhibitory strengths but differing activation strengths—the first group exhibited stronger activation effects than the second. In both trajectories, genes *x*_3_, *x*_4_, and *x*_7_ within the negative feedback loops displayed the most significant changes. In the first trajectory, strong activation maintained near-constant expression levels for *x*_4_ and *x*_7_ (Appendix Fig. S4A). In contrast, weaker activation in the second trajectory led to a decline in *x*_4_ and *x*_7_ expression before stabilization (Appendix Fig. S4B). Notably, the *x*_3_ expression increased more prominently in the second trajectory compared to the first. Of note, upregulation of *x*_2_ in this simulated system was assumed to trigger cell division and enhanced population growth (see details in the Methods section).

For the 3-gene network data, GRNvelo successfully reconstructed cellular velocities, trajectories, latent time, and growth patterns (Fig. 2B–E), accurately distinguishing between the two dynamic states (Appendix Fig. S2C,D). The model precisely captured the true velocity vectors of individual cells in 3D space (Fig. 2B) and correctly assigned each cell to its respective developmental trajectory (Fig. 2C). The derived latent time effectively positioned cells along their differentiation courses, with cells in the shorter linear trajectory (exhibiting minimal state changes, particularly during t1–t4) being correctly clustered within unified time windows. The latent time (Fig. 2D) demonstrated strong correlation with ground truth (Pearson’s *r* = 0.81, *p* < 0.01; Fig. 2H), outperforming conventional velocity-based pseudotime estimates (*r* = 0.73, *p* < 0.01; Appendix Fig. S2E,F). We observed a strong concordance between the computed growth dynamics (Fig. 2E) and the mathematical gradients of growth with respect to the expression of three genes (Fig. 2F). This alignment indicates that the inferred regulatory influences of these genes on growth are consistent with the actual phenotypic outcomes, with analysis correctly identifying *x*_1_ as the sole upregulated growth gene. GRN reconstruction (Fig. 2G) correctly revealed the activation effect of *x*_1_ on *x*_2_.

For the 10-gene network data (Fig. 2I), GRNvelo also reconstructed cell differentiation velocities (Fig. 2J) and trajectories (Fig. 2K), with latent time (Fig. 2L) showing exceptional temporal accuracy (*r* =  0.97, *p* < 0.01; Fig. 2P) compared to conventional velocity-based pseudotime (*r* = 0.90, *p* < 0.01; Appendix Fig. S4E,F). Growth dynamics (Fig. 2M) captured accelerated cell division during bifurcation points and stabilization upon differentiation completion. Gene-gradient (Fig. 2N) mapping correctly identified *x*_2_ as the proliferation driver, while GRN reconstruction (Fig. 2O) accurately predicted the activation of *x*_3_*/x*_4_ by *x*_10_ and the inhibition of *x*_2_ by *x*_6_.

To quantitatively benchmark GRNvelo’s performance on the synthetic data, we conducted systematic comparisons with four state-of-the-art trajectory inference methods: (1) TrajectoryNet (Tong et al, 2020) (balanced optimal transport with continuous normalizing flows), (2) MIOFlow (Huguet et al, 2022) (balanced optimal transport with geodesic autoencoders), (3) TIGON (Sha et al, 2024) (unbalanced optimal transport with deep learning), and (4) PI-SDE (Jiang and Wan, 2024) (physics-informed neural networks with Hamilton-Jacobi equations). PRESCIENT was not included in this benchmark because its requirement for pre-specified proliferation rates is incompatible with our synthetic data, where such prior information is absent. Our evaluation assessed both velocity accuracy (magnitude and direction) and pseudotime estimation performance, benchmarking GRNvelo against other methods. Across all metrics (Fig. 2Q–S), GRNvelo demonstrated superior performance. For the 3-gene network, GRNvelo achieved the best overall velocity reconstruction (magnitude and direction) across all five time points, with Pearson correlation between latent time and true time reaching 0.81. In the 10-gene network, GRNvelo maintained the best performance in velocity reconstruction at all time points and achieved exceptional temporal accuracy (Pearson correlation = 0.97). Comparative analysis revealed method-specific characteristics: PI-SDE showed improved velocity magnitude fitting at later time points; TIGON exhibited weaker initial time point predictions but performed well at intermediate (3-gene) and most (10-gene) time points; MIOFlow demonstrated better performance on the 10-gene network; while TrajectoryNet showed inconsistent performance, with particularly poor fitting on the 10-gene network. Additionally, we benchmarked GRNvelo against ten GRN inference methods: GRNVBEM (Sanchez-Castillo et al, 2018), LEAP (Specht and Li, 2017), GENIE3 (Huynh-Thu et al, 2010), CellOracle (Kamimoto et al, 2023), GRNBoost2 (Moerman et al, 2019), TIGON (Sha et al, 2024), SINCERITIES (Gao et al, 2018), PPCOR (Kim, 2015), SCODE (Matsumoto et al, 2017), and GRISLI (Aubin-Frankowski and Vert, 2020). GRNvelo consistently outperformed alternatives in both network configurations, while GENIE3, SINCERITIES and GRISLI showed better accuracy on the 10-gene network compared to the 3-gene system (Fig. 2T). These results collectively demonstrate GRNvelo’s superior and robust performance across different network complexities and evaluation metrics.

To assess the adaptability of our framework to different contexts, we evaluated GRNvelo using simulation data generated from dynamical equations used in TIGON’s simulation study (Appendix Fig. S5). These simulations are characterized by two key features: (1) cooperative gene–gene interactions in their governing equations, and (2) two distinct cell populations with markedly different differentiation rates (a feature also present in our own simulations). Remarkably, GRNvelo successfully reconstructed the cellular velocity fields and developmental trajectories while accurately identifying population-specific growth patterns and correctly pinpointing the key growth-regulating genes in both populations. The robust performance of GRNvelo across these varied biological assumptions, despite not being trained on such cooperative interaction dynamics, suggests its good generalization capability and broad applicability to diverse cellular processes.

To assess whether the performance of GRNvelo depends on the specific stochastic modeling framework used for data generation, we further evaluated the method on synthetic data simulated using the Gillespie algorithm, which numerically solves the chemical master equation and captures the intrinsic stochasticity of biochemical reactions without the continuity assumption inherent in SDEs. Using the same 3-gene and 10-gene network topologies, we generated Gillespie-based time-series data and applied GRNvelo for trajectory inference. The Pearson correlation between inferred latent time and true simulation time was 0.81 for the 3-gene network and 0.93 for the 10-gene network (Appendix Figs. S6 and S7), comparable to the results obtained on SDE-generated data (0.81 and 0.97, respectively). These results demonstrate that GRNvelo maintains robust inference performance across distinct stochastic modeling frameworks, supporting its applicability to biological systems where gene expression noise arises from discrete molecular events.

### Validation on pancreatic β-cell differentiation data

**Figure 3:**
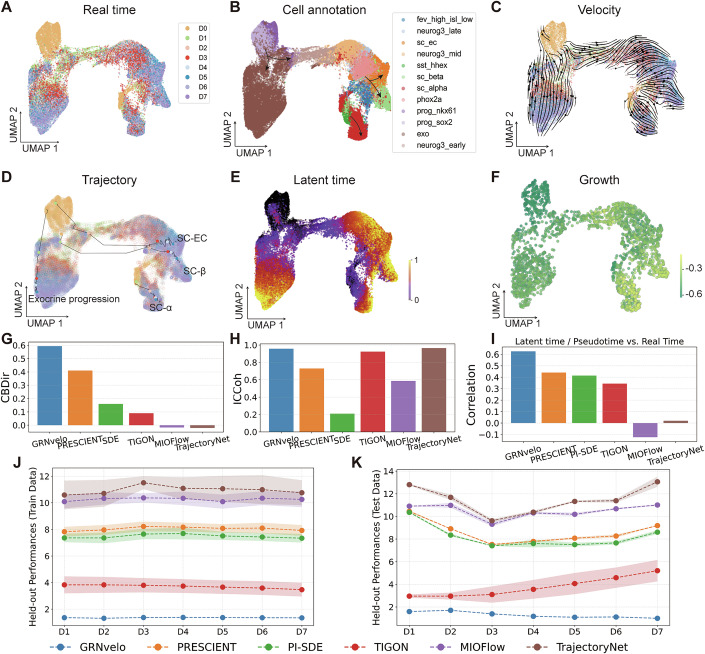
Validation and comparison of GRNvelo on pancreatic β-cell data. (**A**) Experimental time labels. (**B**–**E**) Model predictions. (**B**) Velocity field. (**C**) Trajectory reconstruction. (**D**) Latent time. (**E**) Growth. (**F**) Cell annotations with ground truth trajectory. (**G**) CBDir scores from velocity. (**H**) ICCoh scores from velocity. (**I**) Pearson correlation (Latent time/pseudotime vs. real time). (**J**,** K**) Performance evaluation using cross-validation. Shown are training error (**J**) and prediction error (**K**) on held-out seven time points evaluated using Wasserstein distance (shading: ±SD across five random seeds). Source data are available online for this figure.

Following successful validation on synthetic datasets, we quantitatively evaluated GRNvelo’s performance using single-cell RNA sequencing data from Veres et al.‘s in vitro pancreatic β-cell differentiation study (Veres et al, 2019a; Data ref: Veres et al, 2019b) (Fig. 3A). The dataset comprises 51,274 cells collected across eight time points (D0 to D7), annotated into 12 distinct cell types (Fig. 3B). Through high-resolution scRNA-seq and diffusion pseudotime analysis, combined with functional validation such as glucose-stimulated insulin secretion assays and immunostaining, Verse et al. identified four major differentiation trajectories of pancreatic β-cells: (1) endocrine induction producing Stem Cell-Derived Beta Cells (SC-β) and (2) Stem Cell-Derived Enterochromaffin-like Cells (SC-EC), (3) premature endocrine induction yielding Stem Cell-Derived Alpha-like Cells (SC-α), and (4) exogenous growth factor-induced differentiation into duct-like and acinar-like exocrine lineages (collectively termed Exocrine progression) (Veres et al, 2019a). We applied GRNvelo to reconstruct these four biologically distinct differentiation trajectories (SC-β, SC-EC, SC-α, and Exocrine progression), with particular focus on characterizing their dynamic transitions along each developmental path.

GRNvelo accurately reconstructed all four differentiation trajectories while precisely determining each cell’s position along its developmental path (Fig. 3C,D). Velocity analysis (Appendix Fig. S8) revealed accelerated cellular dynamics during D1–D3, followed by gradual deceleration in D4–D7. Trajectory visualization showed that by D3, cells were positioned closer in the transcriptional state space to the terminal state (D7) than to the initial state (D0), indicating that they had already reached the vicinity of terminal differentiation by this time. These computational findings align with experimental observations by Veres et al. (Veres et al, 2019a), where transient NEUROG3 expression (a key endocrine differentiation driver) peaked at D1, and SC-β/SC-EC cells first emerged at D3. Temporal reconstruction demonstrated strong agreement with experimental timelines, with pseudotime endpoints accurately matching D7 and showing a Pearson correlation of 0.627 (*p* < 0.01) with true time (Fig. 3E). Growth dynamics (Fig. 3F) captured the characteristic proliferation pattern: a progressive decline during differentiation followed by stabilization near completion. This precisely matches the biological process described by Petersen et al. (Petersen et al, 2017), where NEUROG3+ endocrine progenitors showed reduced proliferation (<2%), while subsequent SOX9+ non-endocrine cell expansion restored population growth to ~5%.

Here we present a comprehensive quantitative evaluation of GRNvelo’s performance in reconstructing pancreatic β-cell differentiation dynamics compared to five temporal scRNA-seq data-based velocity inference methods, including PRESCIENT (Yeo et al, 2021), PI-SDE (Jiang and Wan, 2024), TIGON (Sha et al, 2024), MIOFlow (Huguet et al, 2022), TrajectoryNet (Tong et al, 2020) (Appendix Fig. S9). Using cell-type annotations and established differentiation pathways (Fig. 3B) as biological ground truth, we assessed three critical metrics across the eight densely sampled time points: cluster-boundary direction consistency (CBDir), intra-cluster coherence (ICCoh), and pseudotime-real time correlation (Fig. 3G–I). GRNvelo demonstrated superior performance, achieving the highest CBDir score (0.60) for inter-cluster directionality and near-optimal ICCoh (0.95) for intra-cluster consistency. Most notably, GRNvelo's latent time reconstruction showed significantly stronger correlation with experimental timelines (0.627) than all competing methods. Among alternative approaches, PRESCIENT emerged as the second-best performer across all metrics, particularly in capturing inter-cluster relationships. PI-SDE showed limited accuracy in velocity estimation (~0.2) with the poorest intra-cluster coherence, while TIGON and TrajectoryNet exhibited complementary strengths to PI-SDE: in contrast to its poor intra-cluster coherence, both maintained relatively high intra-cluster consistency but struggled with inter-cluster directionality, with TrajectoryNet showing particularly poor pseudotime reconstruction. MIOFlow performed suboptimally across all evaluation dimensions, demonstrating the weakest correlation between pseudotime and real time among all methods compared (Fig. 3I). These systematic comparisons reveal that GRNvelo uniquely balances accurate local velocity estimation with global trajectory reconstruction. While other methods showed fluctuating performance across the different metrics, GRNvelo delivered robust and top-tier biological plausibility in all aspects evaluated.

To further compare the performance of the above methods, we conducted cross-validation by sequentially holding out each non-initial time point as test data while using the remaining time points for training, then evaluating both training and test sets against ground truth using Wasserstein distance (Fig. 3J,K). All methods maintained consistent performance rankings across both training and test sets, ordered from best to worst as: GRNvelo, TIGON, PI-SDE, PRESCIENT, MIOFlow, and TrajectoryNet. The training set evaluation revealed three key observations: (1) prediction accuracy remained stable regardless of which time point was reserved for testing, (2) GRNvelo consistently achieved the highest accuracy with minimal variation across five different random seeds (coefficient of variation <5%), and (3) all methods demonstrated time-invariant performance on their training distributions. More importantly, test set analysis uncovered distinct temporal dependency patterns. GRNvelo showed linearly improving prediction accuracy for later time points, with performance strongly correlated to the number of available preceding time points—a unique feature indicating its ability to effectively accumulate temporal context. In contrast, TIGON exhibited an inverse relationship, where prediction accuracy decreased as more preceding time points were included. The remaining methods (i.e., PI-SDE, PRESCIENT, MIOFlow, and TrajectoryNet) displayed nonlinear temporal patterns, with optimal performance occurring at D3 when the Wasserstein distance reached its minimum.

### Validation using lineage tracing data

**Figure 4:**
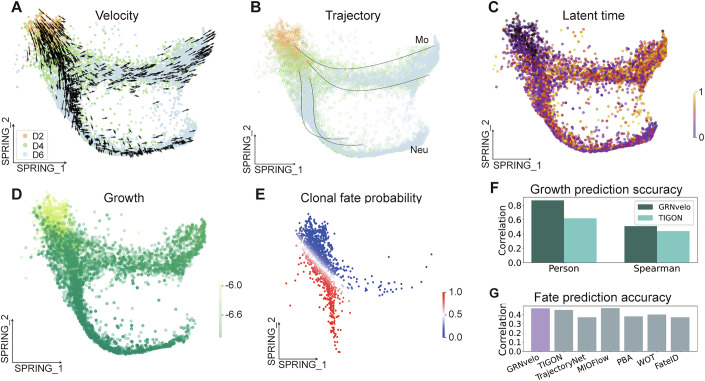
Validation and comparison of GRNvelo using lineage tracing data. (**A**–**D**) Model predictions. (**A**) Velocity field. (**B**) Reconstructed trajectory. (**C**) Latent time. (**D**) Growth. (**E**) The differentiation probabilities predicted by GRNvelo indicate that cells will commit to the Neu fate on D2. (**F**) Correlation between predicted growth rates and experimental ground truth. (**G**) Comparison of d2 cell differentiation probabilities predicted by seven methods with ground truth. Source data are available online for this figure.

To further validate the accuracy of our method in trajectory inference and growth prediction, we applied GRNvelo to a time-series scRNA-seq dataset of murine hematopoiesis with lineage tracing (Weinreb et al, 2020a; Data ref: Weinreb et al, 2020b). This dataset comprises cells collected at three time points (D2, D4, and D6; Fig. 4), where additional barcodes were used to track clones over time, ensuring that cells sharing the same clone originated from a common progenitor at D0. The dataset provides both cellular trajectory and growth information, enabling quantitative validation of our method’s effectiveness. In the lineage tracing data, we compared different computational methods by evaluating their performance in predicting the probability of D2 progenitor cells differentiating into Mo cells. This comparison was based on models trained using data from three discrete time points (D2, D4, and D6).

Consistent with the original study (Weinreb et al, 2020a), we selected clonal cells of neutrophils (Neu) and monocytes (Mo) from D2, D4, and D6 for validation. After batch correction across experiments, the data were projected onto a simplified two-dimensional force-directed layout (SPRING plot). The differentiation process was clearly observable as early progenitors developed into Neu and Mo cells (Fig. 4). In terms of differentiation bias, the reconstructed instantaneous cell transition velocity (Fig. 4A) revealed that a differentiation trend was already detectable at D2 and became more pronounced at later stages. By D6, most cells had progressed toward the distal ends of their respective bifurcating branches, ultimately committing to their fates. Trajectory analysis (Fig. 4B) further confirmed the bifurcation by tracking the differentiation paths of individual cells. The latent time (Fig. 4C) aligned with both the velocity and trajectory directions, increasing as the Neu and Mo trajectories extended. We employed the ground-truth growth profile constructed by (Sha et al, 2024) using shared clonal barcodes for comparison. The inferred growth (Fig. 4D) exhibited Pearson and Spearman correlation coefficients of 0.87 and 0.51, respectively, with the ground truth, outperforming the TIGON method (Fig. 4F).

Next, we quantitatively compared GRNvelo with other six trajectory inference methods: TIGON (Sha et al, 2024), TrajectoryNet (Tong et al, 2020), MIOFlow (Huguet et al, 2022), PBA (Weinreb et al, 2018), WOT (Schiebinger et al, 2019), and FateID (Herman and Sagar 2018). While lineage tracing data track the trajectories of cell clones, computational methods infer trajectories at the single-cell level. Therefore, the experimental data cannot directly provide ground truth for the computationally inferred trajectories. Instead, we compared the fate probabilities of individual cells between experimental and computational results. For each clone, we calculated the proportion of descendant cells adopting new fates based on the fate probabilities assigned to individual cells at D2 using the method described in a previous study (Sha et al, 2024) (see details in Appendix Text S3). The ground-truth fate probabilities at D2 revealed that cells in the upper-right region of the Spring plot predominantly differentiated into Mo cells, those in the lower-left region into Neu cells, while cells in the intermediate zone showed no clear boundary along the bifurcating trajectory (Weinreb et al, 2020a). The GRNvelo -inferred results (Fig. 4E) demonstrated that cells in the upper-right region had a 1.0 probability of differentiating into Mo cells, whereas those in the lower-left region had a 1.0 probability of differentiating into Neu cells. At the transition zone between these two populations, the clonal fate probabilities exhibited uncertainty, which aligned with the experimental observations. Quantitative comparison between the predictions by different methods and ground truth (Fig. 4G) showed that our method achieved a Pearson correlation of 0.467 with the true differentiation outcomes, outperforming all other methods except MIOFlow (0.470). It should be noted, however, that MIOFlow is incapable of predicting growth dynamics. Although the lineage tracing data does not provide strictly quantitative benchmarks, these results collectively validate the multi-task capability of GRNvelo and its robust performance in predicting cell differentiation fate and growth dynamics.

### Delineating CD8 + T cell fate switches during perturbed acute LCMV infection with GRNvelo

**Figure 5:**
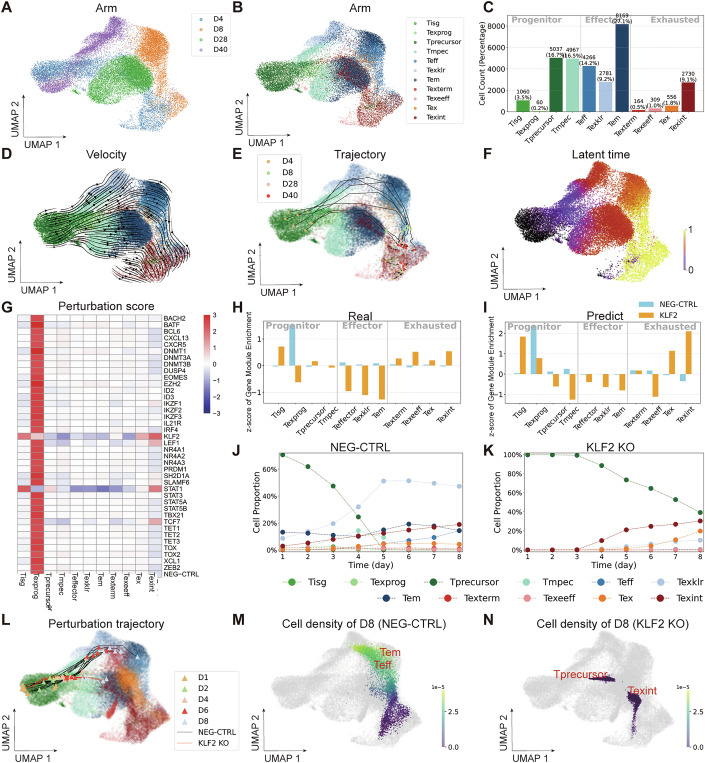
CD8+ T cell differentiation analysis with GRNvelo. (**A**) Experimental timeline. (**B**) Cell-type annotations. (**C**) Population proportions. (**D**, **E**) Model outputs. (**D**) Velocity field. (**E**) Differentiation trajectory. (**F**) Latent time inference. (**G**) D8 gene perturbation z-scores. Model-predicted gene perturbation z-scores for gene modules on D8. The predicted and actual values show a strong Pearson correlation (*r* = 0.80, *p* <  0.01). (**H**) Experimental KLF2 perturbation effects (z-scores by cell type). (**I**) Predicted KLF2 perturbation effects. (**J**, **K**) Temporal population dynamics. (**J**) Unperturbed model. (**K**) KLF2-perturbed model. (**L**) Trajectory comparisons: Black: unperturbed (*n* = 5); Red: KLF2 KO from D1. (**M**, **N**) D8 density predictions: (**M**) Unperturbed. (**N**) Post-perturbation. Source data are available online for this figure.

To further validate GRNvelo effectiveness from a perturbation perspective, we applied it to a set of temporal scRNA-seq data tracking CD8 + T cell differentiation during acute lymphocytic choriomeningitis virus (LCMV) infection (Data ref: Fagerberg, 2024; Fagerberg et al, 2025). The dataset captures a linear differentiation trajectory across four post-infection time points (D4, D8, D28, and D40; Fig. 5A), representing a well-characterized model of antiviral immune responses. Naive CD8 + T cells typically differentiate into distinct effector and memory subsets during acute infections, with their fate determining critical functional outcomes including pathogen clearance, therapeutic responses, and immunopathology. This contrasts with chronic antigen exposure scenarios (e.g., tumors or persistent infections) that promote terminal exhaustion. Original annotations (Fagerberg et al, 2025) classified the CD8 + T cells into 12 subtypes (Fig. 5B), which we categorized into three functional groups: precursor populations (Tisg, Texprog, Tprecursor, Tmpec; 37.0% of cells), effector subsets (Teff, Texklr, Tem; 50.6%), and exhausted populations (Texterm, Texeeff, Tex, Texint; 12.4%) (Fig. 5C). The minimal presence of exhausted cells (12.4%) confirmed the dataset’s representation of acute rather than chronic infection dynamics, preserving the linear differentiation trajectory essential for our analysis. This experimental system provided an ideal platform to test our model’s ability to reconstruct immune cell fate decisions under well-defined biological conditions.

After training on time-resolved transcriptional states, GRNvelo successfully predicted velocity, trajectory, and latent time patterns (Fig. 5D–F) that were consistent with experimentally observed effector lineage progression. The model robustly recapitulates the established effector differentiation pathway, initiating from Tprecursor cells, progressing through Tmpec intermediates, and differentiating into Teff effector cells—a trajectory that matched the DSM pathway described in the original study (Fagerberg et al, 2025). Notably, in the context of acute LCMV infection, the differentiation terminus converges on Texint cell state. This finding is consistent with Fagerberg et al. (Fagerberg et al, 2025), who identified a Tex-like cluster in acute infection, implying a preserved cellular competence for exhaustion-associated differentiation despite antigen clearance. Nonetheless, in contrast to the stable exhaustion in chronic settings, these acute Texint cells are presumably transient, and their definitive fate remains an open question for subsequent studies. Single-cell velocity predictions (Appendix Fig. S10) revealed dynamic changes in differentiation speed, with CD8 + T cells exhibiting accelerated movement during initial differentiation followed by gradual deceleration in later stages. This temporal pattern aligns with the known biological response to acute LCMV infection, where CD8 + T cell expansion peaks around D8 before subsequent contraction and stabilization after 30 days (Wherry et al, 2003). Based on this established kinetics, we focused our subsequent perturbation analyses specifically on the acute differentiation trajectory during the first 8 days post-infection, when the most dynamic cellular transitions occur.

In our perturbation analysis, we rigorously replicated the original experimental design: using our trained model, we performed in silico gene KO of 39 selected genes (including 25 transcription factors (TFs), seven effector molecules (Ems), and seven other genes of interest) at D1 post-infection, with unperturbed cells serving as negative controls (NEG-CTRL) (see details in Methods section). We then examined the resulting differentiation phenotypes at D8 by deriving gene modules from the “differentiation space map” (DSM) and scoring them using Seurat’s AddModuleScore function (Fig. 5G). The model’s KO predictions showed strong correlation with experimental data (Appendix Fig. S11), achieving a Pearson coefficient of 0.80 (*p* < 0.01). Knockout effects revealed distinct TF groups that either promoted effector (e.g., BATF and DNMT1) or memory (e.g., BCL6 and CXCL13) differentiation modules. Notably, KLF2 knockout cells (Fig. 5I) exhibited significant downregulation of effector modules (Teff: z-score = −0.39, *P* < 0.001) accompanied by upregulation of exhaustion-related modules (Texint: z-score = +2.11, *P* < 0.001), suggesting its unique role in suppressing exhaustion during acute infection. Comparative analysis between model predictions and experimental data for KLF2 KO (Fig. 5H,I) demonstrated consistent directional changes: precursor cells (Tisg) and exhausted populations (Tex, Texint) showed increasing z-scores, while effector subsets (Teff, Texklr, Tem) displayed decreasing trends. These results indicate that our model accurately captures CD8 + T cell differentiation trajectories during acute infection and possesses robust generalization capability under gene perturbation conditions.

We subsequently analyzed the model’s predictions in greater detail to examine population size changes for each cell type during D1-D8 differentiation under both NEG-CTRL and KLF2 KO conditions (see details in Appendix Text S4). In unperturbed conditions (Fig. 5J), Tprecursor populations showed a marked decline over time, decreasing from 70.69% at D1 to complete disappearance by D6. Conversely, Texklr populations exhibited significant expansion, increasing from 8.65 to 47.29% over the same period. Other cell types demonstrated more subtle fluctuations. Following KLF2 perturbation (Fig. 5K), differentiation kinetics were substantially slowed, with all cell populations remaining relatively stable during the first three days. This observation aligns with experimental findings that demonstrate impaired effector differentiation (as evidenced by reduced KLRG1 and GzmA expression) leading to blocked differentiation progression (Fagerberg et al, 2025). From D3 onward, we observed pronounced accumulation of exhausted cell populations. Tprecursor populations declined steadily (99.29% at D3 to 39.27% at D8), while Texint exhausted populations increased dramatically (0.28 to 30.45% over the same period). These population shifts correlated well with gene module enrichment results, collectively demonstrating that KLF2 knockout redirects differentiation trajectories from effector to exhausted fates. To further validate these findings, we visualized differentiation trajectory changes for selected cell populations under both conditions from D1 to D8 (Fig. 5L), and compared the distribution and density of all cells at D8 between control and KLF2 KO conditions (Fig. 5M,N). These analyses provide additional evidence that KLF2 knockout effectively switches cellular differentiation from effector to exhaustion pathways.

**Figure 6:**
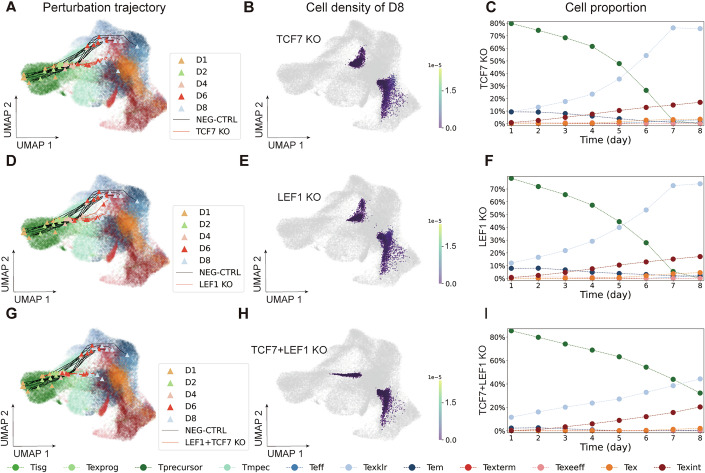
Altered differentiation analysis of CD8+ T cells following multi-gene KO in the GRNvelo model. (**A**–**C**) Effects of TCF7 KO on T cell differentiation: (**A**) Differentiation trajectories of T cell differentiation following TCF7 KO. The differentiation trajectory of TCF7 KO cells (red) is shown relative to the NEG-CTRL (black). (**B**) Density prediction at D8 of TCF7 KO cells. (**C**) Temporal population dynamics of double KO cells. (**D**–**F**) Effects of LEF1 KO on T cell differentiation: (**D**) Differentiation trajectories of T cell differentiation following LEF1 KO. The differentiation trajectory of LEF1 KO cells (red) is shown relative to the NEG-CTRL (black). (**E**) Density prediction at D8 of LEF1 KO cells. (**F**) Temporal population dynamics of double KO cells. (**G**–**I**) Effects of TCF7 and LEF1 double KO on T cell differentiation: (**G**) Differentiation trajectories of T cell differentiation following TCF7 and LEF1 double KO. The differentiation trajectory of double KO cells (red) is shown relative to the NEG-CTRL (black). (**H**) Density prediction at D8 of double KO cells. (**I**) Temporal population dynamics of double KO cells. Source data are available online for this figure.

Building upon the established model, we further investigate the synergistic regulatory roles of TCF7 (which encodes the transcription factor TCF1) and LEF1 in CD8 + T cell differentiation. Compared to the NEG-CTRL, single KO of either TCF7 or LEF1 decelerates the differentiation kinetics of effector cells, although the effector population does not fully abate until D8 (Fig. 6A–F). In contrast, the double KO of TCF7 and LEF1 produces a more pronounced phenotype: memory T cells “Tem” are nearly absent from D4 onward, and effector differentiation progresses even more slowly (Fig. 6G–I). Notably, unlike KLF2 KO, which redirects cellular fate toward exhaustion, TCF7 and LEF1 double KO leads to a substantial increase in terminal effector cells “Texklr” by D8, accompanied by a conversion of the memory cell pool into this terminal effector population. This genetic evidence indicates that the combined loss of TCF7 and LEF1 does not reprogram cellular fate towards exhaustion but specifically compromises memory cell maintenance, skewing it toward a terminal effector fate. This finding strongly aligns with the experimental studies of “LEF1/TCF1” synergy, wherein these two transcription factors act cooperatively to ensure a portion of cells adopt a memory fate rather than fully committing to an effector lineage (Shan et al, 2021; Zhao et al, 2024).

### Deciphering cell-state transitions of NSCLC tumor cells during drug treatments with GRNvelo

**Figure 7:**
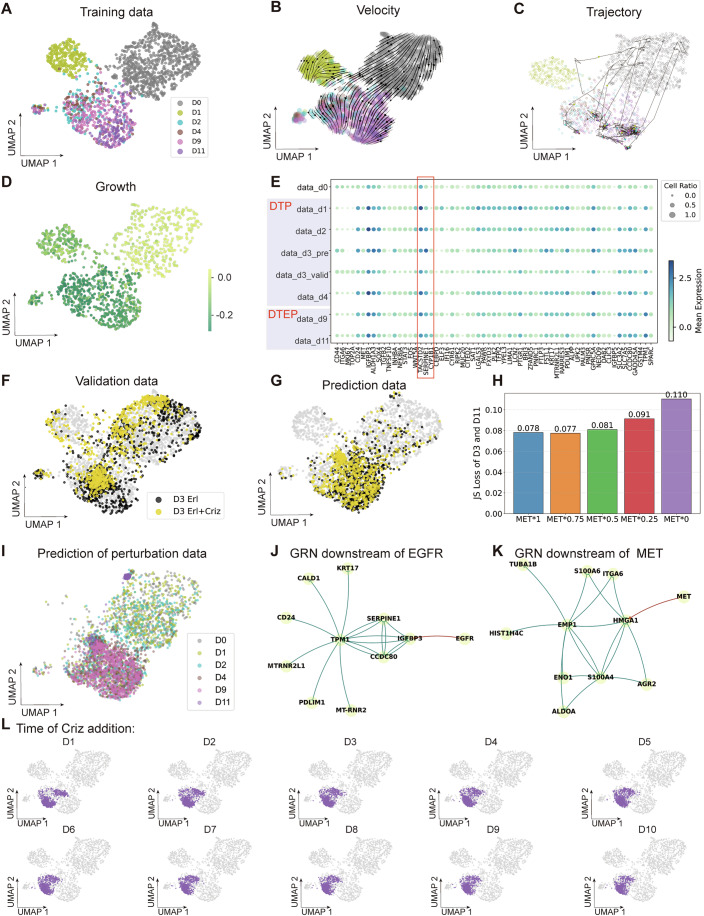
Analysis of drug-resistant PC9 tumor cells using GRNvelo. (**A**) Experimental timeline. (**B**–**D**) Model predictions. (**B**) Velocity field. (**C**) Differentiation trajectory. (**D**) Growth dynamics. (**E**) Gene expression profiles of tumor cells in the training, validation, and prediction sets. Cells from D1 to D4 are in the drug-tolerant persister (DTP) state, characterized by cell cycle exit and quiescence. In contrast, cells from D9 to D11 have transitioned to the drug-tolerant expanded persister (DTEP) state, with partially restored proliferative capacity. The profile represents the broader drug-tolerant (DT) state (including both DTP and DTEP) marker gene set identified through scRNA-seq clustering. TACSTD2, SERPINE1, and CYP1B1 (within the red box) serve as representative validated markers of this set, exemplifying the distinct expression patterns in DT cells. (**F**) Experimental data of D3 cells under Erl treatment alone versus combined Erl and Criz treatment. (**G**) Prediction results of the GRNvelo under Erl treatment and under combined Erl and Criz treatment, with Criz perturbing its target gene MET. (**H**) Therapeutic prediction: distance between perturbed d3 cells and late-stage tumors (D11) under varying Criz doses. (**I**) UMAP visualization post-perturbation. (**J**, **K**) Downstream GRNs: (**J**) EGFR-regulated network. (**K**) MET-regulated network. (**L**) Cell states at D11 following treatment with dynamic combinations of Criz and Erl. Criz is administrated at different time points (D1–D10), while Erl is administrated continuously from D0. Source data are available online for this figure.

To further test GRNvelo’s performance in more challenging scenarios with perturbation conditions, we adopted an alternative validation approach by assessing the effectiveness of the model’s trajectory prediction for cells following gene perturbations. We applied GRNvelo to infer the cell state transition trajectory of drug-resistant PC9 tumor cells in non-small cell lung cancer (NSCLC). Alexandre et al. (Data ref: Aissa et al, 2020; Aissa et al, 2021) demonstrated that the drug erlotinib (Erl) influences the drug-resistant state of PC9 cells by targeting EGFR. Additionally, when combined with Erl, the drug crizotinib (Criz) further modulates the tumor cells’ drug resistance by acting on its target MET (Aissa et al, 2021). We analyzed the tumor cell data from the aforementioned study, which includes two datasets: (1) cell states at D0, D1, D2, D4, D6, D9, and D11 after Erl treatment (Fig. 7A), and (2) cell states at D3 under both Erl monotherapy and Erl&Criz combination treatment (Fig. 7F). The first dataset served as the training set, while the Erl-treatment data from the second dataset were used as the validation set to evaluate model’s predictions. The data with the Erl&Criz combination treatment were designated as the perturbation test set. Both datasets were preprocessed and visualized using UMAP.

The GRNvelo model was trained on single-cell transcriptomic data collected at seven discrete time points following Erl treatment, successfully reconstructing the dynamic changes in cancer cells post-exposure, including velocity, trajectory, and growth patterns (Fig. 7B–D). Velocity analysis (Fig. 7B; Appendix Fig. S12) demonstrated that cell-state transition directions across all time points consistently progressed toward D11, with a notable reduction in transition speed by D11. The trajectory visualization (Fig. 7C) further elucidated the differentiation pathway. Moreover, growth analysis (Fig. 7D) revealed that proliferation rates of both drug-sensitive and drug-resistant cells remained unchanged at D0 prior to drug treatment. However, upon Erl exposure, drug-sensitive cells underwent cell death, resulting in progressively decreasing proliferation rates over time, a finding consistent with experimental data showing cells from D1 to D11 existed in a drug-tolerant (DT) state (Aissa et al, 2021). Cell cycle scoring analysis (Aissa et al, 2021) revealed that cells from D1–D4 were in a drug-tolerant persister (DTP) state, characterized by cell cycle exit and entry into quiescence, while cells from D9 to D11 transitioned to a drug-tolerant expanded persister (DTEP) state with partial recovery of proliferative capacity.

To directly asses the consistency of the established DT markers across independent datasets, we tracked the expression of these marker genes, previously identified by Aissa et al. (Aissa et al, 2021), across our composite dataset including the training set, prediction set, and validation set (Fig. 7E). We focused our targeted analysis on a critical subset of the DT marker gene set: TACSTD2 (tumor-associated calcium signal transducer 2), SERPINE1 (plasminogen activator inhibitor-1), and CYP1B1 (cytochrome P450 family 1 subfamily B member 1). These genes were selected as top candidates for experimental validation in the original study due to their significant differential expression between drug-tolerant populations and drug-naive, sensitive cells and their established roles in cancer biology and drug metabolism. Specifically, TACSTD2, an EpCAM paralog involved in cell adhesion and TGF-β signaling, was highly expressed in DTP cells. SERPINE1, a protease with multiple oncogenic potentials, was upregulated in both DTP and DTEP states. CYP1B1, an enzyme capable of metabolizing erlotinib, showed elevated expression in DTEP cells. The consistent expression patterns of these functionally important markers observed in our prediction set strongly aligned with those in the validation set. These results collectively demonstrate that our model accurately recapitulates the key cellular state transitions during drug treatment, as defined by these established biological landmarks.

Building upon the trained model, we further investigated the combined perturbation effects of Erl and Criz by performing additional perturbations on MET, the target gene of Criz, to examine subsequent changes in cellular transcriptomes. We compared the predicted cell states (Fig. 7G) under single-drug (Erl) and dual-drug (Erl+Criz) treatments at D3 against the experimentally observed states (Fig. 7F). It is worth noting that the experimental data contained a small population of cells mapped to D0, while the majority were distributed in either the DTP (D1–D4) or DTEP (D9–D11) states. Notably, the predicted data showed that nearly all cells localized to these two dominant states, closely matching the primary distribution of the experimental observations. To validate the perturbation results, we systematically modulated MET expression levels and measured the resulting distances between drug-resistant cells and D11 DTEP cells (Fig. 7H). The analysis revealed an inverse relationship: as MET expression decreased, cellular states progressively diverged from the D11 reference, confirming MET’s role in suppressing drug-resistant cell growth. Subsequent prediction of state transitions under combined treatment (MET*0.25 perturbation, Fig. 7I) demonstrated that even at D11, cells predominantly remained in a state transcriptionally resembling early DTP (D1–D4) states, as inferred from their high similarity in gene expression profiles to the training reference, rather than progressing to a state resembling the proliferative DTEP (D9–D11) state. This prediction suggests a novel mechanistic insight that co-inhibition of MET alongside EGFR could not only suppress the overall emergence of resistance but also fundamentally lock surviving cells in a more vulnerable, quiescent DTP state, thereby preventing the acquisition of a more aggressive, proliferative DTEP phenotype. Furthermore, the model reconstructed the downstream gene regulatory networks for both EGFR and MET (Fig. 7J,K). In the reconstructed EGFR-regulated network, Tropomyosin 1 (TPM1) was identified as a topologically central hub (Appendix Table S1), occupying a critical position downstream of EGFR. This computational finding aligns with established literature where TPM1 is a known mediator of mechanisms that inhibit lung cancer proliferation and invasion while promoting apoptosis (Xu et al, 2025). Likewise, analysis of the MET-regulated network pinpointed EMP1 as a key nodal point (Appendix Table S2). While the role of EMP1 in NSCLC remains uncharacterized, its central position in our MET network is intriguing, given that existing studies in other cancers (e.g., pancreatic cancer and gliomas) demonstrate its function in activating PI3K/AKT signaling to drive proliferation, migration, and stemness (Ru et al, 2025; Wang et al, 2019). Collectively, these results illustrate the utility of GRNvelo in GRN analysis for deciphering key players and regulatory mechanisms underlying the effect of the Erl+Criz combination treatment.

Building upon the above findings, we further evaluate the impact of a dynamic combination therapy regimen, where Criz is introduced at various time points (D1–D10) against a background of Erl treatment, on the cellular states at D11 (Fig. 7L). Compared to the dual-drug combination initiated at D0, the D11 cell states resulting from any delayed administration (D1–D10) are consistently less favorable, demonstrating a closer resemblance to the DTEP state (referencing the D9–D11 state from the original data). This underscores the superior efficacy of early combinatorial treatment. Notably, regardless of the specific delay (D1–D10), the D11 cell population was consistently partitioned into two spatially segregated clusters on UMAP: one merging with the DTEP state and the other aligning with the early DTP state (reference D1–D4). Furthermore, as the initiation of combination treatment is progressively delayed, the latter cell subset progressively diverges from the naive D0 state and displays increasingly concentrated state distributions with reduced heterogeneity. These observations collectively reinforce that early co-inhibition more effectively prevents the acquisition of a proliferative DTEP phenotype and instead locks cells into a more vulnerable, quiescent DTP state.

## Discussion

This study developed a multiscale computational framework that integrates time-series single-cell transcriptomic data, nonlinear gene regulatory networks, and cell population dynamics to decipher the dynamic processes underlying cell fate determination. Our approach not only infers gene regulatory dynamics and cellular latent time, but also incorporates phenotype-structured growth dynamics to optimize velocity fields, thereby achieving more accurate characterization of cell trajectories and growth patterns. Validation using both simulated data and experimental datasets (including bifurcation and perturbation scenarios) demonstrates the framework’s robust applicability for studying complex biological processes. This work provides a novel computational tool for single-cell data analysis and offers important insights into the regulatory mechanisms governing cellular behavior during tissue development, immune response, and disease progression and treatment.

Our study offers distinct advantages over existing single-cell trajectory inference methods through its integrated modeling of nonlinear gene regulation and cell population dynamics. Unlike conventional approaches, such as RNA velocity that rely on decoupled dynamics and linearity assumptions, which often fail to accurately capture complex processes like bifurcations or feedback regulation, our model employs Hill functions and neural networks to characterize gene–gene interactions, thereby better representing the high-dimensional and nonlinear characteristics of biological systems. Furthermore, differing from Markov chain-based methods like CellRank (Lange et al, 2022), our method explicitly incorporates cellular proliferation and competition mechanisms, making the inferred cellular dynamics more biologically realistic. These methodological advances enable superior performance in analyzing time-series and perturbation single-cell data, particularly in uncovering dynamic switching of cell fates and reprogramming patterns of regulatory networks, thereby providing novel insights into how external interventions influence cell fate decisions.

GRNvelo models branching trajectories by fitting lineage-specific GRN parameters, which differs from classical multistability frameworks where a single fixed-parameter system gives rise to multiple attractors and stochastic transitions between them (Kang and Li, 2021; Zhang et al, 2014). This design reflects GRNvelo’s focus on predicting cell fate outcomes from time-series snapshot data, particularly in non-stationary developmental processes where the regulatory landscape evolves over time due to external signals or epigenetic remodeling. In such contexts, branch-specific parameterization offers a flexible and data-driven approach for reconstructing trajectory dynamics and inferring terminal fates. We note that fate prediction and mechanistic understanding of stochastic decision-making are complementary goals; integrating data-driven trajectory inference with energy landscape models represents a promising direction for future investigation.

Beyond trajectory inference, GRNvelo offers predictive capabilities for experimental design. It enables biologists to computationally test perturbation hypotheses, predict responses to multi-gene or dynamic interventions, and optimize sampling intervals for time-series experiments. For the datasets analyzed here, GRNvelo identifies key regulators in β-cell differentiation, reveals in CD8 + T cells that higher proliferation rates correlate with a preferential commitment to effector fate over memory fate, and predicts effective drug combinations in resistant tumors, providing testable hypotheses for future investigations.

However, several limitations of the current study warrant further improvements. First, our method partially relies on time-series data and may underperform when applied to static single-cell transcriptomic datasets. Although the ODEs module of the model does not heavily rely on the realistic temporal information, the PDE module requires time-series data for training to infer population density dynamics. Extending GRNvelo to static datasets would require incorporating additional information to compensate for the absence of temporal labels. For the TC‑PINN module, without time‑stamped observations, one could incorporate prior temporal information using genes that are known to correlate with time (e.g., cell‑cycle or differentiation‑associated genes) to constrain the latent time. This approach has precedent in methods that use “clock” genes to order cells, e.g., Revelio (Schwabe et al, 2020) and DeepCycle (Riba et al, 2022). Alternatively, another line of work (Zhu and Wang, 2024; Zhu et al, 2024) reconstructs continuous vector fields directly from RNA velocity without requiring predefined clock genes, which may also inspire this direction. For the MP‑PINN module, which infers cell growth from density changes over time, such dynamics could be estimated in static data by leveraging static spatial snapshots as demonstrated in OSDR (Somer et al, 2026): OSDR uses cell division markers (e.g., Ki67) and the composition of local cellular neighborhoods to infer proliferation rates, effectively reconstructing population dynamics without temporal sequences. Future studies could explore extending the model to non-temporal data through pseudotime analysis or transfer learning techniques.

Second, while we incorporated the consideration of spatial constraints for cell competition in an implicit way, the model does not integrate actual spatial dimension into the model, potentially limiting the accuracy of cellular dynamics inference. Incorporating spatial transcriptomics technologies (e.g., Visium (Stahl et al, 2016) or Stereo-seq (Chen et al, 2022)) could enable more realistic simulations of spatiotemporal dynamics of cells within tissues. Furthermore, although our method integrates intracellular gene regulatory networks with cell population dynamics, it does not explicitly incorporate intercellular interactions, which are recognized as important modulators of cell fate dynamics. Incorporating cell-cell communication and interaction into models of cell population dynamics using single-cell or spatial transcriptomic data remains a significant and challenging goal. While GRNvelo currently focuses on intracellular dynamics from temporal scRNA-seq data, its multiscale PINN-based framework can be extended to spatiotemporal data for inferring cell-cell interactions (CCIs). Existing methods like DeepTalk (Yang et al, 2024) and stMLnet (Yan et al, 2025) incorporate spatial information into cell-cell communication (CCC) inference but fail to connect CCC with phenotypic dynamics. CCCvelo (Yan et al, 2026) couples CCC with single-cell velocity but does not consider the constraint of cell population dynamics (e.g., proliferation, density feedback). In the future, an extended GRNvelo framework could incorporate the modulation of ligand-receptor-mediated CCC on gene expression velocity in TC-PINN, while capturing more general (e.g., nonlocal) CCIs through the MP-PINN module’s explicit modeling of density-dependent dynamics. Such an extension would improve CCI inference accuracy and enable predictive simulations of CCI perturbations. In future work, we aim to develop new mathematical models that combine intercellular signaling, intracellular regulation, and population dynamics, integrated with advanced deep learning algorithms, to infer principles and mechanisms governing MCFD from single-cell and spatial omics data.

In addition, we also evaluated the scalability of GRNvelo on multiple synthetic and real datasets of varying sizes, including a 100,000-cell synthetic dataset and the ~90,000-cell *Caenorhabditis elegans* (*C. elegans*) dataset (Packer et al, 2019; Data ref: Ullrich, 2022). The runtime and memory usage for all tested datasets are summarized in Appendix Tables S3 and S4. Nevertheless, for most existing and available time-series scRNA-seq datasets (which typically contain fewer than one hundred thousand cells), GRNvelo remains computationally feasible on standard workstations. In future studies, improving the computational efficiency of GRNvelo on large-scale datasets will increase its practical value.

Beyond the developmental and disease-relevant timescales considered here, GRNvelo also holds potential for modeling longer-term evolutionary processes. The framework already captures two key drivers of evolution: growth rate differences among cell states, which generate selective pressure, and GRN-driven phenotypic transitions, which provide heritable variation in the broad sense. Extending GRNvelo to model Darwinian evolution would require incorporating additional components such as mutation accumulation, strict genetic heritability, and population bottlenecks. Such an extension could enable prediction of clonal selection dynamics in cancer progression, adaptive resistance to therapy, and microbial evolution under environmental stress. We envision this as a promising direction for future research.

Collectively, the computational framework developed in this study offers a versatile tool for analyzing single-cell dynamics by bridging gene regulatory networks with cell phenotypic dynamics. The future integration of the GRNvelo framework with spatiotemporal single-cell omics data and cell-cell communication mechanisms (Yan et al, 2026) will position it as a powerful platform for investigating cell fate decisions and perturbation responses, thereby contributing to and enriching the emerging paradigm of AI virtual cells (Bunne et al, 2024) from a multiscale, dynamic perspective.

## Methods

**Table:** Reagents and tools table

Reagent/resource	Reference or source	Identifier or catalog number
**Experimental models**		
**Recombinant DNA**		
**Antibodies**		
**Oligonucleotides and other sequence-based reagents**		
**Chemicals, enzymes and other reagents**		
**Software**		
R-software v4.4.1	https://www.r-project.org/ (R Core Team 2024)	
Python v3.8.19	https://www.python.org/ (Python Software Foundation 2024)	
**Other**		

### GRNvelo method

GRNvelo is built upon a multiscale model that bridges single-cell GRN dynamics with cell population dynamics. Through rigorous mathematical derivation, we demonstrate that under certain conditions, the single-cell GRN dynamics are formally equivalent to the phenotypic transition velocity at the population level. This theoretical foundation motivates the integration of these two concepts under the term “GRN velocity” and the design of a two-phase cooperative optimization algorithm for its inference. In the first stage, we develop the TC-PINN framework, which integrates nonlinear ordinary differential equations describing gene dynamics with a neural network and incorporates structured prior constraints on latent time to infer GRN velocity. In the second stage, we introduce the MP-PINN framework, which couples the cell density dynamics equation with a neural network and embeds multiple physical quantities as regularization terms within the loss function to simultaneously refine the GRN velocity and infer the density evolution and growth dynamics. In the following sections, we describe details of mathematical modeling and computational algorithms in the GRNvelo method.

#### GRN dynamics modeling

In the model, with discrete time-series $\{{t}_{1},{t}_{2},...,{t}_{T}\}$, single-cell gene expression data $\{x\left({t}_{s}\right)\}$ contains *d* genes $\{{x}_{i}({t}_{s})\}$, where $i\in \{1,2,...,d\}$ and *d* is the gene expression embedding dimension after dimensionality reduction. For convenience, it is referred to as a gene in the following description. Let $x\left(t\right):= \left({x}_{1}\left(t\right),{x}_{2}\left(t\right),\cdots ,{x}_{d}\left(t\right)\right)\in {{\mathbb{R}}}^{d}$ denote continuous gene expression dynamics. Considering that gene expression states are regulated by other genes while simultaneously undergoing self-decay, we established an interpretable nonlinear gene expression dynamics model (Eq. (1)) to quantify expression changes for each gene ${x}_{i}\left(t\right),{i}\in \{1,\,2,...,{d}\}$.

Experimental measurements in time-series single-cell transcriptomic data typically occur on a slow timescale (e.g., measured in days), whereas the gene expression and regulation we describe are microscopic phenomena occurring on a fast timescale (e.g., measured in minutes or seconds). Let *t* denote the slow timescale variable and $\widetilde{t}$ the fast timescale variable, with $\widetilde{t}=\frac{t}{{{{\rm{\varepsilon }}}}^{2}}$, where *ε* is a small parameter. Consequently, ${x}_{i}={x}_{i}\left(t\right)={x}_{i}\left({{{\rm{\varepsilon }}}}^{2}\widetilde{t}\right)$, and the kinetic model for gene expression regulation can be described as:

1 $$\frac{1}{{{{\rm{\varepsilon }}}}^{2}}\frac{d{x}_{i}}{d\widetilde{t}}={\sum }_{j}\left({a}_{{ij}}\frac{{{x}_{j}}^{{h}_{{ij}}}}{{K}_{{ij}}^{{h}_{{ij}}}+{{x}_{j}}^{{h}_{{ij}}}}+{b}_{{ij}}\frac{{K}_{{ij}}^{{h}_{{ij}}}}{{K}_{{ij}}^{{h}_{{ij}}}+{{x}_{j}}^{{h}_{{ij}}}}\right)-{d}_{i}{x}_{i}.$$

The dynamics of gene ${x}_{i}$ in the above equation are determined by three distinct regulatory components: (1) the activating regulation from other genes ${x}_{j}$ represented by the first term on the right-hand side, (2) the inhibitory regulation from other genes ${x}_{j}$ captured in the second term, and (3) the intrinsic degradation of gene ${x}_{i}$ described by the third term. The activation coefficient ${a}_{{ij}}$ takes a non-zero value when gene ${x}_{j}$ promotes the expression of ${x}_{i}$, and 0 otherwise; similarly, the inhibition coefficient ${b}_{{ij}}$ equals non-zero if gene ${x}_{j}$ suppresses ${x}_{i}$, and 0 otherwise. The parameter ${K}_{{ij}}$ denotes the expression level of gene ${x}_{j}$ required to achieve 50% of maximal regulatory effect, while ${h}_{{ij}}$ characterizes the cooperative multi-molecular interactions of gene ${x}_{j}$. The parameter ${d}_{i}$ represents the degradation rate constant of gene ${x}_{i}$.

#### Coupling GRN with phenotypic dynamics

Let$$\,V\left(x\right){{\rm{:= }}}\left({V}_{1}\left(x\right),\,{V}_{2}\left(x\right),\,\ldots ,{V}_{d}\left(x\right)\right)\in {{\mathbb{R}}}^{d}$$, where

$${V}_{i}\left(x\right)={\sum }_{j}\left({a}_{{ij}}\frac{{{x}_{j}}^{{h}_{{ij}}}}{{K}_{{ij}}^{{h}_{{ij}}}+{{x}_{j}}^{{h}_{{ij}}}}+{b}_{{ij}}\frac{{K}_{{ij}}^{{h}_{{ij}}}}{{K}_{{ij}}^{{h}_{{ij}}}+{{x}_{j}}^{{h}_{{ij}}}}\right)-{d}_{i}{x}_{i},\,i\in \{1,2,\cdots ,d\}.$$

The system of equations in Eq. (1) can be expressed as $\frac{1}{{{{\rm{\varepsilon }}}}^{2}}\frac{{dx}}{d\widetilde{t}}=V\left(x\right)$, which describes the rate of change in the cellular gene expression state driven by GRN dynamics.

Next, we integrate the single-cell GRN dynamics into the phenotypic dynamics of the cell population. Here, the phenotypic distribution of cells is described by a time-dependent function $\widetilde{\rho }\left(x,\widetilde{t}\right)$, interpreted as the probability density of being in phenotypic state *x* at time $\widetilde{t}$. By the law of mass conservation, we use the following equation to describe the temporal change of the cellular state probability density:

2 $$\frac{\partial \widetilde{\rho }}{\partial \widetilde{t}}\left(x,\widetilde{t}\right)=	-k\left(x\right)\widetilde{\rho }\left(x,\widetilde{t}\right)+\int _{{{\mathbb{R}}}^{d}}K\left(y,x-y\right)\widetilde{\rho }\left(y,\widetilde{t}\right){dy}\\ 	 +\left(1-\frac{{\int }_{{{\mathbb{R}}}^{d}}\widetilde{\rho }\left(x,\widetilde{t}\right){dx}}{M}\right)\widetilde{g}\left(x,\widetilde{t}\right)\widetilde{\rho }\left(x,\widetilde{t}\right).$$

This equation can be viewed as modifying the general form of the master equation (Gardiner, 1986) by incorporating a growth term $\left(1-\frac{{\int }_{{{\mathbb{R}}}^{d}}\widetilde{\rho }{dx}}{M}\right)\widetilde{g}\widetilde{\rho }$, where $\widetilde{g}$ is a function related to cell growth and *M* represents the maximal carrying capacity. The kernel function $K\left(y,\widetilde{u}\right)$ in the above equation describes the transition rate probability density from phenotypic state *y* to phenotypic state $y+\widetilde{u}$ per unit time through processes such as cell differentiation, where $\widetilde{u}$ represents the phenotypic displacement per unit time and has physical dimensions of velocity. The function $k\left(x\right){{\rm{:= }}}{\int }_{{{\mathbb{R}}}^{d}}K\left(x,\widetilde{u}\right)d\widetilde{u}$ denotes the total probability that a cell transitions from phenotype *x* to any other phenotypic state per unit time.

To quantify the phenotypic dynamics dominated by changes in the GRN dynamics $V\left(x\right)$, the kernel $K\left(\cdot ,\cdot \right)$ is defined as:

3 $$K\left(y,\widetilde{u}\right){{\rm{:= }}}{det \left(\widetilde{B}\left(y\right)\right)}^{-1/2}{{\mathscr{K}}}\left({\left|\widetilde{B}{\left(y\right)}^{-1/2}\left(\widetilde{u}-\widetilde{V}\left(y\right)\right)\right|}^{2}\right),$$

where $\widetilde{V}\left(x\right)=\frac{{dx}}{d\widetilde{t}}$. Using the timescale transformation from *t* to $\widetilde{t}$, it follows naturally that $\widetilde{V}={{{\rm{\varepsilon }}}}^{2}V$.

Here, ${{\mathscr{K}}}{{\mathscr{:}}}{{\mathbb{R}}}^{+}\to {{\mathbb{R}}}^{+}$ is a chosen smooth probability density function. Specifically, we use a Gaussian function to construct the phenotypic transition kernel:

$${{\mathscr{K}}}\left({\left|w\right|}^{2}\right)=\frac{1}{{\left(2\pi \right)}^{d/2}}{e}^{-{\left|w\right|}^{2}/2}.$$

In the above Eq. (3), $\widetilde{B}\left(y\right)=\left({\widetilde{B}}_{{ij}}\right)$ is a *d*-dimensional positive definite matrix, for which there exists a unique positive definite matrix $\widetilde{B}{\left(y\right)}^{-1/2}$ such that $\widetilde{B}{\left(y\right)}^{-1/2}\cdot \widetilde{B}{\left(y\right)}^{-1/2}=\widetilde{B}{\left(y\right)}^{-1}$. Each element ${\widetilde{B}}_{{ij}}(i,j\in \{1,2,\ldots ,d\})$ is continuous and bounded. Specifically, we take $({\widetilde{B}}_{{ij}})=2{\varepsilon }^{2}D\cdot \left({{{\rm{\delta }}}}_{{ij}}\right)$, where *D* relates to the intensity of random fluctuations in the cellular phenotypic state, and ${{{\rm{\delta }}}}_{{ij}}$ is the Kronecker symbol, which equals 1 if i=j otherwise 0.

To capture the behavior of the cell population at the macroscopic scale, considering small-amplitude, high-frequency transitions between phenotypic states, we rescale Eq. (2) (see details in Appendix Text S1), yielding the following equation:

4 $$\frac{\partial {\rho }_{\varepsilon }}{\partial t}\left(x,t\right)=	-\frac{1}{{\varepsilon }^{2}}\left[{k}_{\varepsilon }\left(x\right){\rho }_{\varepsilon }\left(x,t\right)-\int _{{{\mathbb{R}}}^{d}}\frac{1}{{\varepsilon }^{d}}{K}_{\varepsilon }\left(y,\frac{x-y}{\varepsilon }\right){\rho }_{\varepsilon }\left(y,t\right){dy}\right] \\ 	 +\frac{1}{{\varepsilon }^{2}}\left(1-\frac{\int _{{{\mathbb{R}}}^{d}}{\rho }_{{{\rm{\varepsilon }}}}\left(x,t\right){dx}}{M}\right){g}_{{{\rm{\varepsilon }}}}\left(x,t\right){\rho }_{\varepsilon }\left(x,t\right),$$

where the functions ${K}_{{{\rm{\varepsilon }}}}\left(\cdot ,\cdot \right)$ and ${k}_{{{\rm{\varepsilon }}}}\left(\cdot \right)$ are defined as:

5 $${K}_{\varepsilon }\left(y,u\right)	 ={\det \left(B\left(y\right)\right)}^{-1/2}{{\mathscr{K}}}\left({\left|B{\left(y\right)}^{-1/2}\left(u-{{\rm{\varepsilon }}}V\left(y\right)\right)\right|}^{2}\right) > \,0,\,{k}_{\varepsilon }\left(y\right)\\ 	=\int _{{{\mathbb{R}}}^{d}}{K}_{\varepsilon }\left(y,u\right){du},$$

with $$\left({B}_{{ij}}\right)=2D\cdot \left({\delta }_{{ij}}\right)$$. Consequently,

6 $${K}_{\varepsilon }\left(y,u\right)={(2D)}^{-d/2}{{\mathscr{K}}}\left({\left|{(2D)}^{-d/2}\left(u-{{\rm{\varepsilon }}}V\left(y\right)\right)\right|}^{2}\right) > \,0.$$

The function ${g}_{{{\rm{\varepsilon }}}}$ is defined as ${g}_{{{\rm{\varepsilon }}}}\left(x,t\right)=\widetilde{g}\left(x,\frac{t}{{{{\rm{\varepsilon }}}}^{2}}\right)=\widetilde{g}\left(x,\widetilde{t}\right)$, still representing cell growth, and we assume that ${{\mathrm{lim}}}_{\widetilde{t}\to \infty }\widetilde{t}\widetilde{g}\left(x,\widetilde{t}\right)$ exists.

We can rigorously prove that as $\varepsilon \to 0$, the solution ${\rho }_{\varepsilon }$ of Eq. (4) converges in the weak sense to the solution *ρ* of the following equation (theorem and proof detailed in Appendix Text S2):

7 $${\partial }_{t}\rho +{\nabla }_{x}\cdot (V\rho )=D{\Delta }_{x}\rho \left(x,t\right)+\left(1-\frac{\int _{{{\mathbb{R}}}^{d}}\rho \left(x,t\right){dx}}{M}\right)g\rho .$$

Equation (7) describes cell population dynamics and captures several key biological processes through its different terms. On the left-hand side, the first term quantifies the rate of cell density change over time, while the second term models the convective flow resulting from phenotypic transition with the velocity *V* that leads to density redistribution. The right-hand side components include: (1) a growth term modulated by population size constraints, representing nonlocal density-dependent proliferation and (2) a diffusion term accounting for random cellular epimutation, where *D* denotes the diffusion coefficient of cell states in the phenotype space, quantifying how readily intrinsic cellular or environmental noise drives phenotypic transitions. Together, these components form a biologically meaningful framework for analyzing population-level cellular behaviors.

#### Multiscale model

The above derivation has connected single-cell GRN dynamics with cell population phenotypic dynamics, resulting in a unified ODE-PDE system:

8 $$\left\{\begin{array}{c}\frac{{dx}}{{dt}}=V\left(x\right),\\ {\partial }_{t}\rho +{\nabla }_{x}\cdot \left(V\rho \right)=D{\Delta }_{x}\rho +\left(1-\frac{{\int }_{{{\mathbb{R}}}^{d}}\rho \left(x,t\right){dx}}{M}\right)g\rho .\end{array}\right.$$

This multiscale modeling approach maps sing-cell GRN velocity to cell fate dynamics in the context of cell growth and random epimutation, building a theoretical foundation of the following two-phase cooperative optimization algorithm that integrates TC-PINN and MP-PINN.

#### GRN velocity inference via TC-PINN

To address the challenges posed by the nonlinearity of the model and the sparse temporal sampling of the data in the inverse problem of GRN dynamics, we develop the TC-PINN by integrating the governing equations of GRN dynamics into a PINN architecture. Moreover, beyond the conventional physics-informed and data fidelity loss terms, we introduce a novel structured temporal constraint derived from the network’s internal learning process. This constraint explicitly enforces temporal consistency across discretely observed time points, thereby significantly enhancing the physical plausibility and accuracy of the inferred dynamical models. The discrete time-series data were represented as $\left({t}_{1},{X}\left({t}_{1}\right)\right),\,\left({t}_{2},X\left({t}_{2}\right)\right),\,\ldots ,({t}_{T},{X}({t}_{T}))$, where $X({t}_{i})={\left\{{x}_{j}({t}_{i})\right\}}_{j=1}^{{N}_{i}}\in {{\mathbb{R}}}^{{N}_{i}\times d}$, where ${N}_{i}$ denotes the number of cells at the discrete time point ${t}_{i}$ and *d* denotes the number of genes in each cell. For the discrete data, we first classify all cells into *m* cluster-level lineages, with each cell assigned to a specific lineage $({t}_{1},\,{X}^{({J}_{k})}\left({t}_{1}\right)),({t}_{2},{X}^{({J}_{k})}\left({t}_{2}\right)),\ldots ,({t}_{T},\,{X}^{({J}_{k})}\left({t}_{T}\right))$, where${X}^{({J}_{k})}\left({t}_{i}\right)={\left\{{x}_{j}^{({J}_{k})}({t}_{i})\right\}}_{j=1}^{{N}_{i}}\in {{\mathbb{R}}}^{{N}_{i}^{({J}_{k})}\times d}$, $k\in \{1,\,2,\ldots ,{m}\}$ (see the following section for details). For each trajectory ${J}_{k}$, we select a root cell $\{{x}_{0}^{({J}_{k})}\}$ at the initial time point ${t}_{1}$ and use its gene expression profile as initial conditions. We then construct a multilayer neural network $N{N}_{1}(\theta )$ that takes the gene expression values of the root cell and time *t* as inputs, and outputs the predicted gene expression $\hat{x}(t)$ at time *t*. We define the composite loss function as specified in the following Eqs. (9–12).

9 $${Loss}={\lambda }_{1}{Los}{s}_{{data}}+{\lambda }_{2}{Los}{s}_{f}+{\lambda }_{3}{Los}{s}_{{lt}},$$

10 $${Los}{s}_{{data}}=\frac{1}{T}{\sum }_{i=1}^{T}\left(\frac{1}{{N}_{i}^{({J}_{k})}}{\sum }_{j=1}^{{N}_{i}^{({J}_{k})}}{\left|{x}_{j}^{\left({J}_{k}\right)}\left({t}_{i},{s}_{l}\right)-\hat{x}\left({s}_{l}\right)\right|}^{2}\right),$$

11 $${Los}{s}_{f}=\frac{1}{d}{\sum }_{i=1}^{d}{\left|\frac{d{\hat{x}}_{i}}{{dt}}-{\sum }_{j}\left({a}_{{ij}}\frac{{\hat{x}}_{j}^{{h}_{{ij}}}}{{K}_{{ij}}^{{h}_{{ij}}}+{\hat{x}}_{j}^{{h}_{{ij}}}}+{b}_{{ij}}\frac{{K}_{{ij}}^{{h}_{{ij}}}}{{K}_{{ij}}^{{h}_{{ij}}}+{\hat{x}}_{j}^{{h}_{{ij}}}}\right)+{d}_{i}{\hat{x}}_{i}\right|}^{2},$$

12 $${Los}{s}_{{lt}}={\sum }_{i=1}^{T-1}\varphi \left({\mu }_{{t}_{i+1}}-{\mu }_{{t}_{i}}\right),\,\varphi \left(\omega \right)=\left\{\begin{array}{c}0,\,\omega \ge 0,\,\\ 1,\hfill\,\omega < 0.\end{array}\right.$$

The loss function in our ODE training framework consists of three key components. The data discrepancy term (${{Loss}}_{{data}}$) quantifies differences between model predictions $\hat{x}\left(s\right)$ and experimental measurements ${x}_{j}^{\left({J}_{k}\right)}\left({t}_{i},\,{s}_{l}\right)$ at discrete time points ${t}_{i}$, where $i\in \left\{1,\,2,\ldots ,{T}\right\},j\in \left\{1,\,2,\ldots ,\,{N}_{i}^{({J}_{k})}\right\}$. Here, ${s}_{l}$ denotes the dense discrete time point of the model prediction that is closest to ${x}_{j}^{\left({J}_{k}\right)}\left({t}_{i}\right)$ (see the following section for details). The physics-based regularization term (${{Loss}}_{f}$) enforces consistency with the governing equation (Eq. (1)), that is, the left-end term minus the right-end term of the GRN dynamics equation, where the left-hand term $\frac{d{\hat{x}}_{i}}{{dt}}$ is obtained through automatic differentiation of the neural network output with respect to time *t*, while the right-hand terms are computed from model parameters. The time constraint term ${{\mathrm{Loss}}}_{{lt}}$ imposes temporal constraints on the inferred latent time. ${\mu }_{{t}_{i}}$ denotes the maximum value of the latent time among all cells at discrete time point ${t}_{i}$, i.e., ${\mu }_{{t}_{i}}={\max }_{{IT}}{\left\{{IT}\left({x}_{j}^{\left({J}_{k}\right)}\left({t}_{i}\right)\right)\right\}}_{j=1}^{{N}_{i}^{({J}_{k})}}$, where ${IT}\left({x}_{j}^{\left({J}_{k}\right)}\left({t}_{i}\right)\right)={\min }_{{s}_{l}}{||}{x}_{j}^{\left({J}_{k}\right)}\left({t}_{i},\,{s}_{l}\right)-\hat{x}\left({s}_{l}\right){||}$. The loss weights ${\lambda }_{1}-{\lambda }_{3}$ were set to 1 by default and optimized via grid search within [0,1] using a validation set. Sensitivity analysis on the synthetic 3-gene dataset (varying each weight by ±20 and ±40%) showed minimal impact on velocity directions and only slight fluctuations in magnitudes, confirming the robustness of the model with respect to these loss weights (Appendix Fig. S13A).

In Eq. (11), ${a}_{{ij}}$ and ${b}_{{ij}}$ denote the maximum transcriptional activation and repression rates, respectively, from regulator gene *j* to target gene *i*. Specifically, ${a}_{{ij}}$ scales the Hill activation term, while ${b}_{{ij}}$ scales the Hill repression term. These parameters collectively define the strength and type (activation or repression) of each regulatory interaction. Biologically, a regulatory interaction is either activation or repression, but not both. Consequently, during training, we enforce that for each gene pair (i,j), at most one of ${a}_{{ij}}$ and ${b}_{{ij}}$ is non-zero (i.e., either ${a}_{{ij}} > \,0$ and ${b}_{{ij}}=0$, or ${a}_{{ij}}=0$ and ${b}_{{ij}} > \,0$, or both zero if no regulation exists). Within the TC‑PINN module, ${a}_{{ij}}$ and ${b}_{{ij}}$ are treated as trainable variables and are jointly estimated with the neural network weights by minimizing the composite loss function (including ${{Loss}}_{{data}}$, ${{Loss}}_{f}$ and ${{Loss}}_{{lt}}$). The specific combination of ${a}_{{ij}}$ and ${b}_{{ij}}$ directly determines the predicted velocity. Different combinations yield distinct velocity fields, which govern the dynamical evolution of gene expression. Incorporating ${a}_{{ij}}$ and ${b}_{{ij}}$ increases the model interpretability.

The model training consists of two sequential steps (Fig. 1B). In the initial training step (Step 1), we set$\,{\lambda }_{3}=0$, initialize (see the following section for details) and fix the latent times of all cells based on available temporal information while optimizing the TC-PINN parameters to capture global dynamical patterns. In the fine training step (Step 2), we jointly optimize both the model parameters and individual cell latent times through an alternating procedure that enforces mutual constraints between the learned dynamics and temporal assignments. Specifically, each iteration of this alternating optimization consists of two sub-steps: first, the parameters of the neural network are held fixed, and the optimal latent time assignment is estimated for each observed data point (See the following section for details); second, the latent time assignments are fixed, and the neural network parameters are updated by minimizing the loss function via the Adam algorithm. This alternating process is repeated iteratively, progressively refining the estimates until convergence to a consistent solution is achieved. The GRN velocity V(x,t) for each cell is computed via automatic differentiation of the neural network’s output with respect to the input time variable *t*, where *t* corresponds to the specific latent time of the cell. Note that this velocity incorporates gene–gene interactions, and is therefore referred to as GRN velocity to distinguish it from existing RNA velocity inference based on splicing dynamics.

#### Cell fate dynamics inference via MP-PINN

We develop the MP-PINN framework that combines cellular density dynamics with neural networks to model cell-state transitions and growth. The model architecture consists of a multilayer neural network ${{\mathrm{NN}}}_{2}(\theta )$ that takes cellular expression profiles x(t) and their corresponding time points *t* as inputs, and outputs three key biological quantities: local cell density ($\hat{\rho }$), velocity ($\hat{V}$), and growth rate ($\hat{g}$). We customized a composite loss function with multiple physical constraints, defined as Eqs. (13–17).

13 $${Loss}={\gamma }_{1}{{Loss}}_{\rho }+{\gamma }_{2}{{Loss}}_{{V}_{{size}}}+{\gamma }_{3}{{Loss}}_{{V}_{{direction}}}+{\gamma }_{4}{{Loss}}_{F},$$

14 $${{Loss}}_{\rho }=\frac{1}{{TK}}{\sum }_{j=1}^{T}{\sum }_{i=1}^{K}{\left|\hat{\rho }\left({x}_{i},\,{t}_{j}\right)-\rho \left({x}_{i},\,{t}_{j}\right)\right|}^{2},$$

15 $${{Loss}}_{{V}_{{\mathrm{size}}}}=\frac{1}{{TK}}{\sum }_{j=1}^{T}{\sum }_{i=1}^{K}{\left|\hat{V}\left({x}_{i},\,{t}_{j}\right)-\kappa V\left({x}_{i},\,{t}_{j}\right)\right|}^{2},$$

16 $${{Loss}}_{{V}_{{d}{i}{r}{e}{c}{t}{i}{o}{n}}}=\frac{1}{{TK}}{\sum }_{j=1}^{T}{\sum }_{i=1}^{K}\left(1-\frac{\hat{V}({x}_{i},\,{t}_{j})V({x}_{i},\,{t}_{j})}{\left|\hat{V}({x}_{i},\,{t}_{j})\right|\left|V({x}_{i},\,{t}_{j})\right|}\right),$$

17 $${{Loss}}_{F}={\partial }_{t}\hat{\rho }+{\nabla }_{x}\cdot \left(\hat{V}\hat{\rho }\right)-D{\Delta }_{x}\hat{\rho }-\left(1-\frac{{\int }_{x}\hat{\rho }\left(x,t\right){dx}}{M}\right)\hat{g}\hat{\rho }.$$

The composite loss function integrates four critical components to ensure both biological fidelity and physical consistency in our model. First, ${{\mathrm{Loss}}}_{\rho }$ quantifies discrepancies in cell density estimation between model predictions (($\hat{\rho }\left({x}_{i},\,{t}_{j}\right)$) and cell density estimation data ($\rho \left({x}_{i},\,{t}_{j}\right)$) (see the following section for details). Second, ${{\mathrm{Loss}}}_{{V}_{{size}}}$ penalizes deviations in the magnitude of predicted cellular velocities ($\hat{V}\left({x}_{i},\,{t}_{j}\right)$) from the GRN velocity of TC-PINN ($V\left({x}_{i},\,{t}_{j}\right)$). Third, ${{\mathrm{Loss}}}_{{V}_{{direction}}}$ serves as a key regularization term that maintains directional alignment between the PDE-derived velocity field ($\hat{V}({x}_{i},\,{t}_{j})$) and TC-PINN solutions ($V({x}_{i},\,{t}_{j})$), ensuring coherent cellular trajectories. Fourth, ${Los}{s}_{F}$ enforces fundamental physical constraints by minimizing residuals in the governing PDE equation (Eq. (8)). The GRN velocity derived from TC-PINN is defined on a latent timescale, whereas MP-PINN outputs velocity on the real timescale. To account for this difference in scales, the hyperparameter *κ* is used to scale the velocity discrepancy. The parameter *T* denotes the number of discrete time points. During training, for each time point $j\in \{1,\,\ldots ,{T}\}$, a subset of *K* cells are randomly selected from the total population in the dataset to compute the loss. Thus, *K* defines the number of randomly chosen cells used per time point for training. The loss weights ${\gamma }_{1}-{\gamma }_{4}$ were set to 1 by default and optimized via grid search within [0,1]. Sensitivity analysis on the synthetic 3-gene dataset (varying each weight by ±20 and ±40%) confirmed model robustness, with negligible effects on velocity directions and minor fluctuations in magnitudes (Appendix Fig. S13B). This multi-faceted loss structure achieves three essential objectives: (1) preserving data-consistency through ${{Loss}}_{\rho }$ and ${{Loss}}_{{V}_{{size}}}$, (2) maintaining mathematical rigor via ${{\mathrm{Loss}}}_{F}$, and (3) ensuring dynamical coherence through ${{Loss}}_{{V}_{{direction}}}$. The balanced integration of these components enables robust inference of cellular dynamics that simultaneously respects experimental observations and fundamental biophysical principles. The model optimizes the loss function through the Adam algorithm and updates the parameters of the network until the model converges. This approach enables five key analytical capabilities at the population level: (i) refinement of GRN velocity under environmental constraints, (ii) reconstructing continuous trajectories through temporal integration $({\int }_{0}^{t}V(x,t){dt})$ to, (iii) prediction of growth dynamics, (iv) derivation of environment-constrained GRN via velocity-gene derivatives ${\left\{\frac{\partial {V}_{i}}{\partial {x}_{j}}\right\}}_{i,j=1}^{d}$, and (v) simulation of perturbation trajectories through in silico genetic manipulations.

#### Identification of cluster-level lineages

The TC-PINN framework first determines the cluster-level lineages to which each cell belongs, as different lineages correspond to distinct model parameters. Given that some cells may bifurcate during differentiation, each lineage is associated with a unique set of ODEs that needs to be parameterized. The number of cluster-level lineages *m* is set to the number of categories of cell type annotations at the final time point ${t}_{T}$, which can be derived either from prior knowledge or by performing clustering analysis on the data at ${t}_{T}$. Based on cluster-level lineages $\{{J}_{1},\,{J}_{2},\ldots ,\,{J}_{m}\}$, cells at each discrete time point are partitioned accordingly. To be specific, for time-series data spanning $\{{t}_{1},\,{t}_{2},\ldots ,\,{t}_{T}\}$, we employ K-means clustering or Gaussian mixture clustering to divide the cells at each time point into *m* subsets, simultaneously obtaining the centroid cell for each cluster. Each of the *m* lineages is assigned a unique starting cluster from the *m* clusters identified at the initial time point ${t}_{1}$. For cells at subsequent time points, the Euclidean distance between each cluster centroid and the centroids of the preceding time point is computed, and the cluster with the minimal distance is assigned to the same lineage. This process yields the complete set of cells belonging to each lineage. Finally, the cells of the *k*-th lineage are denoted as $({t}^{1},\,{X}^{({J}_{k})}({t}_{1})),\,({t}^{2},{X}^{({J}_{k})}({t}_{2})),\ldots ,\,({t}_{T},\,{X}^{({J}_{k})}({t}_{T}))$, where ${X}^{({J}_{k})}({t}_{i})={\{{x}_{j}^{({J}_{k})}({t}_{i})\}}_{j=1}^{{N}_{i}}\in {{\mathbb{R}}}^{{N}_{i}^{({J}_{k})}\times d},{k}\in \{1,\,2,,{m}\}$, ${N}_{i}^{({J}_{1})}+\ldots +{N}_{i}^{\left({J}_{m}\right)}={N}_{i}$.

#### Identification of root cells

For each cluster-level lineage obtained by the TC-PINN, determining the root cell is important for deriving subsequent velocity values using ODEs. Given a cluster-level lineage with time-series data across *T* time points $\{{t}_{1},\ldots ,\,{t}_{T}\}$, where each time point ${t}_{i}$ contains ${N}_{i}^{({J}_{k})}$ cells, the root cell must be selected from the ${N}_{1}^{({J}_{k})}$ cells at the initial time point ${t}_{1}$. Theoretically, the root cell should exhibit the greatest relative distance to all cells at the next time point (*t*_2_). To identify the root cell, we leverage the ${N}_{2}^{({J}_{k})}$ cells at ${t}_{2}$ for computation. For each of the ${N}_{1}^{({J}_{k})}$ cells at ${t}_{1}$, we compute its distance to every cell at ${t}_{2}$ and retain the smallest distance as the representative distance between the cell of ${t}_{1}$ and the population of ${t}_{2}$. Among these minimal distances, the cell of ${t}_{1}$ associated with the largest minimal distance (i.e., the most distant from the population of *t*_2_) is designated as the root cell (Eq. (18)).

18 $${x}_{{root}}^{\left({J}_{k}\right)}\leftarrow d\left({x}_{{root}}^{\left({J}_{k}\right)},\,{x}_{{t}_{2}}^{\left({J}_{k}\right)}\right)={\max }_{i}\left\{{\min }_{j}\left\{{{||}{x}_{i}^{\left({J}_{k}\right)}\left({t}_{1}\right)-{x}_{j}^{\left({J}_{k}\right)}\left({t}_{2}\right){||}}_{{L}^{2}}\right\}\right\},$$

where $$i=1,\,\ldots ,\,{N}_{1}^{({J}_{k})},\,j=1,\,\ldots ,\,{N}_{2}^{({J}_{k})}.$$

#### Initial temporal assignment of cells

Once the root cell is identified, we assign initial latent time values to all time points to facilitate subsequent velocity training. For each cluster-level lineage spanning *T* time points ${t}_{1},\ldots ,\,{t}_{T}$}{, where each time point ${t}_{i}$ contains ${N}_{i}^{({J}_{k})}$ cells, we define the latent time on the interval [0,1]. The [0,1] time range is evenly divided into *P* segments. For cells at time point ${t}_{i}$, their latent times are sampled from a normal distribution with a mean of $\left(\frac{i-1}{P}+\frac{i}{P}\right)/2$ and a standard deviation of 0.05. This stochastic assignment ensures that all cells are assigned an initial latent time while maintaining temporal coherence with measured time points. The root cell is explicitly assigned a latent time of 0.

#### Iterative refinement of gene dynamics and cellular latent time

In the TC-PINN framework, after establishing the ability of the model to capture global cell trends, the potential time allocation of individual cells is continued to be refined. The refinement process involves the following steps: First, we discretize the [0,1] interval into *L* (e.g., 1000 or 2000) equally spaced dense time points ${s}_{l}$, where ${s}_{l}=\frac{1}{L}* l,(l=1,\,2,\ldots ,{L})$. Using our trained model, we then compute the predicted gene expression values $\hat{x}\left({s}_{l}\right)$ at each of these time points.

Following the model prediction step, we calculate the Euclidean distance between the gene expression profile of each cell and the model-predicted values across all sampled time points. The total distance for each cell is obtained by aggregating the individual gene-wise distances. The optimal latent time for each cell ${x}_{j}^{\left({J}_{k}\right)}\left({t}_{i},{s}_{l}\right)$ is then determined by selecting the time point corresponding to the minimum aggregate distance (Eq. (19)).

19 $${x}_{j}^{\left({J}_{k}\right)}\left({t}_{i},{s}_{l}\right)\leftarrow {\min }_{{s}_{l}}{{||}{x}_{j}^{\left({J}_{k}\right)}\left({t}_{i}\right)-\hat{x}\left({s}_{l}\right){||}}_{{L}^{2}},(l=1,\,2,\ldots ,\,L).$$

After assigning updated latent times to all cells, we incorporate the mean squared error (MSE) between observed and predicted gene expression values, ${Loss}={MSE}\left({x}_{j}^{\left({J}_{k}\right)}\left({t}_{i},{s}_{l}\right),\,\hat{x}\left({s}_{l}\right)\right)$, as a component of the total loss function (Eq. (10)). This metric drives the iterative refinement process using the Adam optimization algorithm.

#### Cell density estimation

We estimate the cell density from time-series data in the reduced-dimensional space. The discrete time-series data is represented as $({t}_{1},\,{C}_{1}),({t}_{2},\,{C}_{2}),\ldots ,({t}_{T},\,{C}_{T})$, where ${C}_{i}={\left\{c{\left(j\right)}_{{t}_{i}}\right\}}_{j=1}^{{N}_{i}}\in {{\mathbb{R}}}^{{N}_{i}\times d}$ denotes ${N}_{i}$ independent and identically distributed samples drawn from a *d*-dimensional space at time ${t}_{i}$. In the absence of prior mass information, the sample size ${N}_{i}$ is proportional to the relative cell abundance. The density ${\rho }_{{t}_{i}}$ is estimated using a Gaussian mixture model (Sha et al, 2024; Shewhart et al, 2000) incorporating ${N}_{i}$ equally-weighted Gaussian distributions, each corresponding to a sample point. Each distribution is centered at its respective sample point with a covariance matrix $\Sigma =\sigma I\in {{\mathbb{R}}}^{d\times d}$, where *σ* represents a constant standard deviation for all sample points. The final density ${\rho }_{{t}_{i}}$ is obtained by multiplying the Gaussian mixture distribution by the relative population size ${N}_{i}/{N}_{1}$, normalized to the initial time point ${t}_{1}$.

##### Pseudo code of GRNvelo algorithm


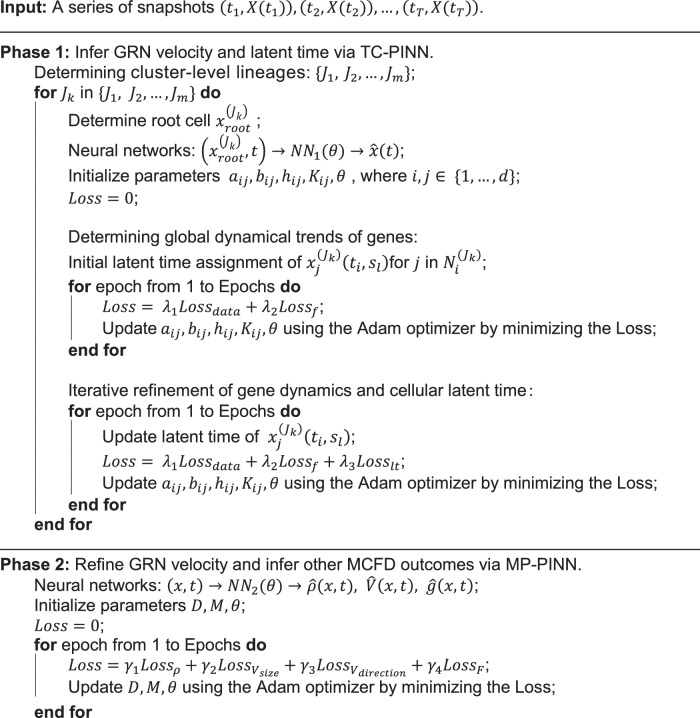


### Simulated data generation

In this study, we employ stochastic differential equations (SDEs) to generate two synthetic datasets: one comprising a 3-gene regulatory network (Fig. 2A) and another with a 10-gene network (Fig. 2I). The gene–gene regulatory relationships are modeled by Hill kinetics (Hill et al, 1910; Weiss, 1997). The dynamical behavior of each gene is described by the following equation:

20 $$\frac{d{x}_{i}}{{dt}}=S+{\sum }_{j}\left({a}_{{ij}}\frac{{x}_{j}^{{h}_{{ij}}}}{{K}_{{ij}}^{{h}_{{ij}}}+{x}_{j}^{{h}_{{ij}}}}+{b}_{{ij}}\frac{{K}_{{ij}}^{{h}_{{ij}}}}{{K}_{{ij}}^{{h}_{{ij}}}+{x}_{j}^{{h}_{{ij}}}}\right)-{d}_{i}{x}_{i}+{\delta }_{i}{\xi }_{t},$$

In the aforementioned equations, *S* represents an external input signal as described below, where ${a}_{{ij}}$ denotes the activation strength of other gene ${x}_{j}$ on gene ${x}_{i}$, and ${b}_{{ij}}$ represents its repression strength on ${x}_{i}$. The parameter ${K}_{{ij}}$ corresponds to the substrate ${x}_{j}$ concentration at which the production ${x}_{i}$ rate reaches half of its maximum value, while ${d}_{i}$ indicates the degradation rate of gene ${x}_{i}$. The term ${\delta }_{i}{\xi }_{t}$ captures additive white noise accounting for stochastic effects in gene expression. For both simulated datasets, the cell division probability is positively correlated with the expression level of a specific gene (*x*), following the relationship: $g \sim \frac{{x}^{2}}{1+{x}^{2}} \%$.

We simulate two distinct cellular differentiation trajectories within a 3-gene regulatory network by implementing two parameter sets under identical external signaling conditions ($S=0.1* \frac{{0.1}^{2}}{1+{0.1}^{2}}$). For the first trajectory, initial cell states were sampled from a three-dimensional normal distribution $N([0.5,\,0.1,\,0.4],\,0.01)$, with degradation rates set as ${d}_{1}={d}_{2}={d}_{3}=0.1$. The network dynamics incorporate activation strengths (${a}_{21}=0.3,\,{a}_{31}={a}_{22}=0.1$), inhibition (${b}_{12}=0.2$), and Hill function parameters (${K}_{21}={K}_{31}={K}_{22}=1;{h}_{21}={h}_{31}={h}_{22}=2$), along with stochastic noise (${\delta }_{1}={\delta }_{2}={\delta }_{3}=0.01,\,{\xi }_{{{\rm{t}}}}\sim N(0,1)$). The second trajectory initialize cells from $N([0.6,\,0.2,\,0.3],\,0.01)$ with modified parameters including ${d}_{1}=0.2$ and distinct regulatory strengths (${b}_{21}=0.03,{a}_{21}=0.1,\,{a}_{31}=0.05,\,{a}_{22}=0.01,\,{b}_{12}=0.1$), while maintaining identical Hill and noise parameters. Both trajectories share a proliferation mechanism where division probability scaled with ${x}_{1}$ expression ($g=0.5* \frac{{x}_{1}^{2}}{1+{x}_{1}^{2}} \%$).

We implement a 10-gene regulatory network using the established NET1 architecture from the DREAM4 benchmark (Greenfield et al, 2010) to simulate two distinct cellular differentiation trajectories. There are no external signals in this network, that is, S=0. Both trajectories are initialized with cells sampled from identical ten-dimensional normal distributions $N([0.3,\,0.8,\,0.4,\,1,\,0.2,\,0.7,\,0.2,\,0.3,\,0.2,\,0.9],\,0.01)$ and shared common baseline parameters: no external signaling input (S=0), uniform degradation rates (${d}_{i}=0.1$ for all genes), stochastic noise (${\xi }_{t}\sim N(0,1)$ with amplitude ${\delta }_{1}=0.01$), and consistent Hill function parameters (${h}_{{ij}}=2,\,{K}_{{ij}}=1$ for all regulatory interactions). The network topology incorporates experimentally-derived inhibitory edges (${b}_{{ij}}=0.2$) and activating edges, with the key distinction between trajectories being their activation strengths–the first trajectory used ${a}_{{ij}}=0.1$ while the second employed weaker activations (${a}_{{ij}}=0.05$). Both systems maintained the critical biological feature of proliferation probability being positively correlated with ${x}_{2}$ expression levels ($g=0.2* \frac{{x}_{2}^{2}}{1+{x}_{2}^{2}} \%$).

### Real datasets

For the Verses2019 dataset (pancreatic β-cell data) (Veres et al, 2019a; Data ref: Veres et al, 2019b), the data (GSE114412) comprises transcriptomic profiles of 51,274 cells across 12 distinct cell types. The Verses dataset includes cells from D0 to D7, capturing the detailed progression of stage 5 pancreatic β-cell differentiation. In the raw data, genes expressed in fewer than ten cells are filtered out from an initial set of 16,225 genes. From the remaining genes, the top 2500 highly variable genes (those exhibiting substantial expression variability across cells) are selected for downstream analysis. During dimensionality reduction, following the original study’s approach, we remove the cell cycle-related gene TOP2A and genes showing a Pearson correlation greater than 0.15 with TOP2A. After this preprocessing, the final dataset contains 51,274 cells and 2500 genes for analysis.

For the lineage tracing data (Weinreb et al, 2020a; Data ref: Weinreb et al, 2020b), following the original study, we select a total of 15,007 cells from D2, D4, and D6, which belong to clones that differentiate into neutrophils (Neu) and monocytes (Mo). We perform validation using the two-dimensional force-directed layout (SPRING) space derived from simplified metadata.

For the CD8 + T cell differentiation data (Data ref: Fagerberg, 2024; Fagerberg et al, 2025), the original data contains data on differentiation trajectories following acute and chronic infections. Here we focus on cells involved in the acute infection differentiation pathway for analysis. We directly utilize the preprocessed single-cell data from the original study, which includes samples from D4, D8, D28, and D40, comprising 50,577 cells and 16,710 genes.

For the NSCLC drug treatment dataset (Data ref: Aissa et al, 2020; Aissa et al, 2021), we use the seven discrete-time datasets under Erl treatment of the original study as the training set, and the datasets on D3 under Erl and Erl+Criz treatment as the validation set (GSE: GSE149383). The dataset comprises two components: (1) samples collected at D0, D1, D2, D4, D6, D9, and D11 following Erl treatment, which undergo cell/gene filtering and normalization, retaining 1,723 cells and 8,437 genes; (2) samples from D3 post-Erl treatment and D3 post-combination treatment with Erl and Criz, which are preprocessed with cell/gene filtering and normalization, resulting in 2008 cells and 8437 genes.

For each dataset, the hyperparameters used in the TC-PINN and MP-PINN networks are provided in Appendix Tables S5 and S6, respectively.

### Dimensionality reduction and visualization

For datasets with gene dimensions exceeding 10, we first reduce the dimensionality to ten using an autoencoder (AE). The resulting reduced-dimensional representations are termed gene expression embeddings. We adopted the default AE architecture following that used in TIGON (Sha et al, 2024), which consists of a fully connected encoder and decoder with a single hidden layer of 300 units, ReLU activation, and a latent dimension of 10. The model is trained to minimize the mean squared error (MSE) reconstruction loss using the Adam optimizer (learning rate = 1e-3, weight decay = 1e-4, batch size = 128) for up to 500 epochs with early stopping (patience = 30). We then apply the GRNvelo method to process these embeddings, obtaining velocity fields, trajectory predictions, growth patterns, and pseudotime estimates derived from the velocity analysis. Finally, we project the results into a two-dimensional space using UMAP for visualization purposes.

### Velocity metric evaluation

#### Velocity metric evaluation across simulated datasets

We evaluate the accuracy of velocity estimation in synthetic datasets through two complementary metrics assessing distinct aspects of the predicted vector field. For magnitude validation, we compute the mean squared error (MSE) between the predicted and ground truth velocity magnitudes, which quantitatively measures the precision of estimated cellular state transition rates. Directional accuracy is assessed through cosine similarity analysis between the true (*V*) and predicted ($\hat{V}$) velocity vectors, calculated as $\cos (V,\hat{V})=({V\cdot }\hat{V})/({||V||\cdot ||}\hat{V}{||})$. This directional metric ranges from −1 to 1, where values approaching 1 indicate perfect alignment between predicted and reference directions, while values near 0 suggest orthogonal vectors. The cosine metric’s sign additionally distinguishes between correctly predicted developmental progression (positive values) and erroneous regression predictions (negative values).

#### Velocity metric evaluation for real datasets

To quantitatively evaluate the biological validity of estimated velocity fields in real datasets, we implement a dual-metric approach combining cross-boundary direction correctness (CBDir) and intra-cluster coherence (ICCoh) (Qiao and Huang, 2021). The CBDir metric quantitatively assesses whether velocity vectors correctly predict known cell-state transitions by measuring the angular alignment between predicted directions and biologically validated differentiation pathways across cluster boundaries. This directional accuracy metric is computed as

$${CBDir}\left(c\right)=\frac{1}{{Norm}}{\sum }_{{c}^{{\prime} }\in {C}_{A}\cap N(c)}\frac{{V}_{c}\cdot ({x}_{{c}^{{\prime} }}-{x}_{c})}{\left|{V}_{c}\right|\left|{x}_{{c}^{{\prime} }}-{x}_{c}\right|},$$

where ${Norm}=\left|{c}^{{\prime} }\in {C}_{A}\cap N(c)\right|$, ${C}_{A}$ is sets of cells in target cluster *A*, *N(c)* stands for the neighboring cells of specified cell *c* and *c*′. ${V}_{c},\,{x}_{c{\prime} },\,{x}_{c}$ are the low-dimensional vectors representing computed velocity and positions of cell *c* and *c*′. Higher values indicating stronger agreement with experimental observations of cellular progression. Complementarily, ICCoh evaluates the local consistency of velocity patterns within annotated cell states by analyzing the variance of vector directions among neighboring cells that share the same cell type classification, where lower variance indicates more coherent developmental trajectories. The ICCoh metric is computed as follows:

$${ICCoh}\left(c\right)=\frac{1}{{Norm}}{\sum }_{{c}^{{\prime} }\in {C}_{A}\cap N(c)}\frac{{V}_{c}\cdot ({x}_{{c}^{{\prime} }})}{\left|{V}_{c}\right|\left|{x}_{{c}^{{\prime} }}\right|}.$$

### Trajectory metric evaluation

#### Wasserstein distance

We quantify the distributional differences between predicted trajectories and trajectories using the Wasserstein distance (Panaretos and Zemel, 2019; Schmidt and Duell, 2025), a metric that fundamentally represents the minimum energy required to transform one probability distribution into another. Suppose there are two discrete probability distributions *P* and *Q*, where the first distribution contains *m* sample points and the second contains *n* sample points. Given two discrete probability distributions, each defined by its characteristic representation matrix:

$$P={\sum }_{i=1}^{m}{p}_{i}{\delta }_{{x}_{i}},Q={\sum }_{j=1}^{n}{q}_{i}{\delta }_{{y}_{j}},$$

where each ${x}_{i}\in {{\mathbb{R}}}^{d}$ and ${y}_{j}\,\in \,{{\mathbb{R}}}^{d}$ represent a sample point distributed in the *d*-dimensional feature space. ${p}_{i}$ and ${q}_{j}$ are the probability weights corresponding to the sample points ${x}_{i}$ and ${y}_{j}$, respectively. *δ* is the Dirac function. Wasserstein distance is defined as:

$${W}_{2}\left(P,Q\right)={\left({\inf }_{\gamma \in \Gamma \left(P,Q\right)}{\sum }_{i=1}^{m}{\sum }_{j=1}^{n}{\gamma }_{{ij}}\cdot d{\left({x}_{i},{y}_{j}\right)}^{2}\right)}^{1/2},$$

where $d{\left({x}_{i},{y}_{j}\right)}^{2}={\left|{x}_{i}-{y}_{j}\right|}_{2}^{2}$ is the square Euclidean distance between the point pairs. $\Gamma \left(P,Q\right)$ represents the set of all coupled matrices that satisfy marginal constraints:

$$\Gamma \left(P,\,Q\right)=\left\{\gamma \in {{\mathbb{R}}}_{+}^{m\times n}\left|{\sum }_{j=1}^{n}{\gamma }_{{ij}}={p}_{i},{\sum }_{i=1}^{m}{\gamma }_{{ij}}={q}_{j}\right.\right\}.$$

#### Jensen-Shannon divergence

The Jensen-Shannon (JS) Divergence (Fuglede et al, 2004) is a symmetric and bounded measure of the dissimilarity between two probability distributions *P* and *Q*. It is derived from the Kullback–Leibler (KL) divergence but addresses its asymmetry by introducing a mixture distribution M=1/2(P+Q). The JS divergence computes the average KL divergence from each distribution to *M*, ensuring symmetry and avoiding infinite values that may arise in the KL divergence when Q(x)=0 but P(x)>0. Mathematically, JS divergence is defined as:

$${D}_{{JS}}\left(P{||Q}\right)=\frac{1}{2}{D}_{{KL}}\left(P{||M}\right)+\frac{1}{2}{D}_{{KL}}\left(Q{||M}\right),$$

where ${D}_{{KL}}$ denotes the KL divergence.

### In silico perturbation

#### Perturbation of single-gene KO in the T cell dataset

To simulate the effects of a genetic perturbation in the CD8 T cell dataset (Fagerberg et al, 2025), we model the instantaneous changes in gene expression following a targeted gene knockout. Consider a system with *n* cells and *d* genes, where the baseline gene expression is denoted by the matrix ${x}_{0}=\{{{x}_{0}}_{{gene}}\}\in {{\mathbb{R}}}^{n \times d}$. To simulate the knockout of a specific gene (e.g., gene A), we compute the post-perturbation expression ${x}_{0}^{{A}_{{KO}}\,}\in {{\mathbb{R}}}^{n \times d}$. This computation accounts for both the direct ablation of gene A’s expression and the subsequent indirect effects on the expression levels of other genes propagated through the interconnected regulatory network. The formula for ${x}_{0}^{{A}_{{KO}}\,}$ is given by:

$${x}_{0}^{{A}_{{KO}}}={x}_{0}+\Delta {x}_{0}+\Delta {{x}_{0}}_{A}\cdot {NGR}{N}_{* ,A}\cdot \Delta t,$$

where $\Delta {x}_{0}\in {{\mathbb{R}}}^{n \times d}=\{\Delta {{x}_{0}}_{{gene}}\}$ is the direct effect of the knockout, which specifies a change $\Delta {{x}_{0}}_{A}={{-x}_{0}}_{A}$ for gene A and zero change for all other genes (${\left\{\Delta {{x}_{0}}_{{gene}}\right\}}_{{gene}\ne A}$). The matrix ${GRN}\in {{\mathbb{R}}}^{d\times d}$ is derived from the GRNvelo model (see the following section for details) and then normalized to the range [−1,1] to represent the normalized GRN (${{NGRN}}\in {{\mathbb{R}}}^{d\times d}$). Here, ${NGRN}_{* ,A}$ represents the regulatory strength of A on all other genes. The parameter Δt denotes the time interval required for the cellular system to reach a new steady state following the perturbation. This parameter is set as a hyperparameter with a value of 3. The hyperparameter Δt represents the time required for detectable state changes of the system after perturbation. Since no ground-truth value is available, we selected Δt=3 based on two considerations: (i) biological prior: in a study of mitochondrial unfolded protein response (UPRmt) during somatic cell reprogramming, perturbation applied on D1 led to detectable phenotypic changes (e.g., MET markers) on D3 (Ying et al, 2025), suggesting that a characteristic response time of approximately 3 days is plausible for gene regulatory network propagation; (ii) sensitivity analysis: over $\Delta t\in \{1,\ldots ,7\}$, the key metrics (precursor proportion at D8 for CD8 + T cells; transcriptional distance to D11 for PC9 cells) changed monotonically and approximately linearly with Δt (Appendix Fig. S14). We therefore chose the middle value Δt=3 as a representative default. We tested and found that all qualitative conclusions remain consistent across ${\Delta} t\in \{2,\ldots ,5\}$.

#### Perturbation of multi-gene KO in the T cell dataset

Following the method for single-gene perturbation, we further extend our in silico perturbation framework to simulate the concurrent knockout of multiple genes. Consider the same system with *n* cells and *d* genes, and the baseline gene expression matrix ${x}_{0}\in {{\mathbb{R}}}^{n \times d}$. To simulate the knockout of a set of *k* target genes *M*, where $\left|M\right|=k$, we compute the post-perturbation expression ${x}_{0}^{M}\in {{\mathbb{R}}}^{n \times d}$.

For multi-gene knockout, the direct effect $\Delta {x}_{0}$ is defined such that the expressions of all *k* target genes are ablated to zero. Formally, for each gene *g* in the set of target genes *M*, the direct change is $\Delta {{x}_{0}}_{g}={{-x}_{0}}_{g}$, where g∈M. For all other genes not in *M*, the direct change remains zero. The subsequent indirect effects on the entire transcriptome are propagated through the GRN. In this multi-gene perturbation, the set *M* is treated as a unified source of regulatory disruption. The collective indirect effect is calculated by applying the aggregated regulatory influence of the set *M* onto all other genes. The formula for the multi-gene knockout is given by:

$${x}_{0}^{{{Multi}}_{{KO}}}={x}_{0}+\Delta {x}_{0}+\Delta {{x}_{0}}_{M}\cdot {NGR}{N}_{* ,M}\cdot \Delta t.$$

Here, $\Delta {x}_{0}$ is the direct effect matrix, containing the instantaneous ablation of the *k* target genes. $\Delta {{x}_{0}}_{M}\in {{\mathbb{R}}}^{n\times k}$ represents the direct ablation values for the gene set *M*. ${NGRN}\in {{\mathbb{R}}}^{d\times d}$ is the same normalized gene regulatory network matrix derived from the GRNvelo model. ${NGR}{N}_{* ,M}\in {{\mathbb{R}}}^{k\times d}$ denotes the sub-matrix of *NGRN* composed of the columns corresponding to the gene set *M*, representing the regulatory strengths of these genes on all others in the network. The hyperparameter Δt was set as described above (Appendix Fig. S14A).

#### Perturbation with combination treatments in the PC9 dataset

We simulate combination drug effects in the PC9 (Aissa et al, 2021) by systematically modifying gene expression patterns according to known pharmacological interactions. Let ${x}_{0}^{{Erl}}\in {{\mathbb{R}}}^{n\times d}$ denote the initial gene expression matrix for *n* cells and *d* genes under Erl monotherapy. The objective is to compute the initial expression profile ${x}_{0}^{{Erl}+{Criz}}\in {{\mathbb{R}}}^{n \times d}$ under the combined action of Erl and Criz. The perturbation from ${x}_{0}^{{Erl}}$ to ${x}_{0}^{{Erl}+{Criz}}$ is modeled by considering the mechanistic impact of Criz: it primarily targets the MET gene, directly modulating its expression level. This perturbation then propagates through the gene–gene regulatory network, leading to adaptive changes in the expression of other genes. Integrating these direct and network-mediated effects, ${x}_{0}^{{Erl}+{Criz}}$ is given by:

$${x}_{0}^{{Erl}+{Criz}}={x}_{0}^{{Erl}}+\Delta {x}_{0}+\Delta {{x}_{0}}_{{\mathrm{MET}}}\cdot {{\mathrm{NGRN}}}_{* ,{{\mathrm{MET}}}}\cdot \Delta t,$$

where $\Delta {x}_{0}\in {{{{\mathbb{R}}}}}^{n \times d}=\{\Delta {{x}_{0}}_{{\mathrm{gene}}}\}$ represents the direct initial perturbation induced by Criz across all genes in all cells. $\Delta {x}_{0}$ is defined such that the change is confined to the target gene MET ($\Delta {{x}_{0}}_{{\mathrm{MET}}}$), while the changes for all other genes (${\left\{\Delta {{x}_{0}}_{{\mathrm{gene}}}\right\}}_{{\mathrm{gene}}\ne {{\mathrm{MET}}}}$) are set to zero. The matrix ${{GRN}}\in {{{{\mathbb{R}}}}}^{d\times d}$ is derived from the GRNvelo model (see the following section for details) and then normalized to the range [−1,1] to represent the normalized GRN (${{NGRN}}\in {{{{\mathbb{R}}}}}^{d \times d}$). Specifically, the element ${{\mathrm{NGRN}}}_{A,B}$ denotes the regulatory strength of gene *B* on gene *A*. Here, ${{\mathrm{NGRN}}}_{* ,{\mathrm{MET}}}$ (the MET column of *NGRN*), thus represents the regulatory strength of MET on all other genes. The hyperparameter ∆t was set as described above (Appendix Fig. S14B).

#### Dynamic combination treatment perturbation in the PC9 dataset

To model the temporal dynamics of combination therapy, we designed a dynamic perturbation experiment that simulates the effect of adding a second drug, Criz, at various time points during an ongoing first-line treatment, Erl. The core of this simulation lies in executing the perturbation within the original high-dimensional gene expression space, thereby ensuring the biological fidelity of the drug’s mechanistic impact. The specific procedure for simulating the addition of Criz at Day *T* (where *T* ranges from 1 to 10) is as follows:

1. Initial state and low-dimensional projection. The baseline gene expression profile ${x}_{0}^{{Erl}}\in {{\mathbb{R}}}^{n \times d}$ (with *n* cells and *d* genes) is first projected into a 10-dimensional latent space using a pre-trained encoder. This low-dimensional representation ${z}_{0}^{{Erl}}\in {{\mathbb{R}}}^{n \times 10}$ serves as the initial state for the GRNvelo.
2. Evolution under monotherapy: The latent state ${z}_{0}^{{Erl}}$ is evolved forward in time for *T* days under the influence of Erl monotherapy, using the GRNvelo model. This yields the latent state ${z}_{T}^{{Erl}}$, which represents the cellular state after *T* days of Erl treatment.
3. Decoding to high-dimensional space for perturbation: To apply the Criz perturbation, the latent state ${z}_{T}^{{Erl}}$ is decoded back to the full, high-dimensional gene expression space, reconstructing an approximation of the gene expression matrix ${x}_{T}^{{Erl}}\in {\mathbb{R}}^{n \times d}$.
4. Application of combination perturbation: The effect of adding Criz to the Erl-treated cells at day *T* is simulated on ${x}_{T}^{{Erl}}$. As Criz directly targets the MET gene, we model this as a perturbation to the MET expression level, which then propagates through the gene regulatory network. The post-Criz expression profile ${x}_{T}^{{Erl}+{Criz}}$ is computed as:
  $${x}_{T}^{{Erl}+{Criz}}={x}_{T}^{{Erl}}+\Delta {x}_{T}+\Delta {{x}_{T}}_{{MET}}\cdot {NGR}{N}_{* ,{MET}}\cdot \Delta t.$$
  Here, $\Delta {x}_{T}$ is the direct effect matrix at time *T*, where only the MET gene column is non-zero ($\Delta {{x}_{T}}_{{MET}}$), representing the immediate downregulation of MET by Criz. NGRN is the normalized gene regulatory network, and Δt is the network propagation time hyperparameter set to 3, as described above.
5. Re-projection and final evolution: The perturbed high-dimensional expression ${x}_{T}^{{Erl}+{Criz}}$ is then projected back into the ten-dimensional latent space, obtaining a new latent state ${x}_{T}^{{Erl}+{Criz}}$, which represents the system’s state immediately after the combination perturbation at Day *T*. This combined state is subsequently evolved forward in the model from Day *T* until the common endpoint of D11. The final output is the predicted cellular state at D11 for the specific scenario where Criz was introduced at Day *T*.

By executing this workflow for each *T* from 1 to 10, we generate a set of comparable predictions, all reflecting the transcriptional outcome at the unified endpoint of D11, but each conditioned on a different timing of Criz addition.

### GRN inference

To handle high-dimensional gene expression data, GRNvelo is trained using the low-dimensional cell embeddings obtained from the encoder. Consequently, the GRN governing interactions in the original high-dimensional gene space is recovered through the following formulation:

$${GR}N=\frac{{{\rm{d}}}{V}_{h}}{{{\rm{d}}}{x}_{h}}=\frac{{{\rm{d}}}\left(\frac{{{\rm{d}}}{x}_{h}}{{{\rm{d}}}t}\right)}{{{\rm{d}}}{x}_{h}}=\frac{{{\rm{d}}}\left(\frac{{{\rm{d}}}{x}_{h}}{{{\rm{d}}}{x}_{l}}\cdot \frac{{{\rm{d}}}{x}_{l}}{{{\rm{d}}}t}\right)}{{{\rm{d}}}{x}_{h}}=\frac{{{\rm{d}}}\left({\left(\frac{{{\rm{d}}}{x}_{l}}{{{\rm{d}}}{x}_{h}}\right)}^{-1}\cdot {V}_{l}\right)}{{{\rm{d}}}{x}_{h}},$$

where ${V}_{h}$ and ${x}_{h}$ are the velocity vector and expression state defined in the original high-dimensional gene space, respectively. Their counterparts in the low-dimensional embedding space are ${V}_{l}$ and ${x}_{l}$. In the computational implementation, the pseudo-inverse ${\left(\frac{d{x}_{l}}{d{x}_{h}}\right)}^{-1}$ is calculated using the “torch.linalg.pinv” function from the PyTorch library.

### Reconstruction of downstream GRNs

We construct GRNs downstream of a given perturbed gene (e.g., drug target gene) by combining statistical selection criteria with biological plausibility constraints. The gene selection process retains the top ten most strongly regulated downstream genes based on comprehensive regulatory strength scoring, ensuring focus on the most significant transcriptional relationships. For edge selection, we implement a dual-threshold system that preserves both the top 20% of all potential regulatory interactions by connection strength while guaranteeing minimum network connectivity through an essential requirement: each target gene must maintain at least one regulatory edge to downstream genes, thus preserving core biological relationships.

## Supplementary information

Appendix

Peer Review File

Source data Fig. 2

Source data Fig. 3

Source data Fig. 4

Source data Fig. 5

Source data Fig. 6

Source data Fig. 7

## Data Availability

The datasets and computer code produced in this study are available in the following databases: Modeling computer scripts: GitHub (https://github.com/SunXQlab/GRNvelo).The source data of this paper are collected in the following database record: biostudies:S-SCDT-10_1038-S44320-026-00220-x.
