# Supplementary material for: Multiscale learning of gene network-driven phenotypic dynamics of single cells
Source: Mol Syst Biol. 2026 Jun 5;22(8):1333–59. doi: 10.1038/s44320-026-00220-x (PMC13434742; doi:10.1038/s44320-026-00220-x)
Supplement: Supplementary file 1 [file 44320_2026_220_MOESM1_ESM.pdf]

Appendix for

Multiscale learning of gene network-driven phenotypic dynamics of single cells

Table of Contents

Appendix Texts 3
Appendix Text S1. Scaling transformation of the non-local phenotypic dynamics equation 3
Appendix Text S2. Convergence of the scaled equation to the limit equation 5
Appendix Text S3. Calculating probabilities of progenitor cells differentiating to the Neu fate in mouse hematopoiesis 10
Appendix Text S4. Calculation of cellular cluster population proportions of CD8+ T cells 11
Appendix Figures 12
Appendix Figure S1. Gillespie-based simulation and model validation of a positive autoregulation system 12
Appendix Figure S2. Supplementary information for 3-gene network 13
Appendix Figure S3. Performance of GRNvelo on simulated data under different noise levels 14
Appendix Figure S4. Supplementary information for 10-gene network 15
Appendix Figure S5. The prediction results of GRNvelo on the synthetic data in TIGON ...16
Appendix Figure S6. Gillespie-based simulation and model validation of a 3-gene regulatory network 17
Appendix Figure S7. Gillespie-based simulation and model validation of a 10-gene regulatory network 18
Appendix Figure S8. Visualization of the velocity predicted by GRNvelo in the pancreatic β-cell differentiation data 19
Appendix Figure S9. Visualization of the velocity and pseudotime predicted by the other 5 methods in the pancreatic β-cell differentiation data 21
Appendix Figure S10. Visualization of the velocity predicted by GRNvelo in the acute LCMV infection data 22
Appendix Figure S11. The ground truth for the enrichment of cluster gene modules in the acute LCMV infection data on D8 23
Appendix Figure S12. Visualization of the velocity predicted by GRNvelo in the drug-resistant PC9 tumor cells 24
Appendix Figure S13. Sensitivity analysis of hyperparameters for the TC-PINN and MP-PINN frameworks 25
Appendix Figure S14. Sensitivity analysis of hyperparameter Δt in perturbation simulations 26
Appendix Tables 27
Appendix Table S1. Network centrality scores for genes in the EGFR downstream GRN ..27
Appendix Table S2. Network centrality scores for genes in the MET downstream GRN 27
Appendix Table S3. Computational cost of the GRNvelo model on synthetic datasets 28
Appendix Table S4. Computational cost of the GRNvelo model on real datasets 28
Appendix Table S5. Hyperparameters in neural network of TC-PINN 29
Appendix Table S6. Hyperparameters in neural network of MP-PINN 30

Appendix Texts

Appendix Text S1. Scaling transformation of the non-local phenotypic dynamics equation.

Assuming that the cell state transition is characterized by small amplitude and high frequency, we perform the following scaling transformations on space and time to derive Equation (4) in the main text from Equation (2) in the main text.

Let $K(y,\tilde{u}) = \det\left(\tilde{B}(y)\right)^{-1/2} \mathcal{K}\left(\left|\tilde{B}(y)^{-1/2}\left(\tilde{u} - \tilde{V}(y)\right)\right|^2\right)$ be the kernel function of the Equation (2) in the main text, and assume:

$$\int_{\mathbb{R}^d} \mathcal{K}(|w|^2)\, dw = 1, \quad \int_{\mathbb{R}^d} w\, \mathcal{K}(|w|^2)\, dw = 0, \quad \int_{\mathbb{R}^d} w_i w_j\, \mathcal{K}(|w|^2)\, dw = \delta_{ij}. \tag{S1}$$

These conditions are satisfied, particularly, when $\mathcal{K}(|w|^2) = \frac{1}{(2\pi)^{d/2}} e^{-|w|^2/2}$.

The term $K(y, x-y)$ represents the probability density of transitioning from phenotypic state y to state x, where $\tilde{u} = x - y$ denotes the displacement vector per unit time at the microscopic scale.

For the time scale, switching to a macroscopic perspective, since the transition amplitude is very small, the transition frequency must significantly increase to observe macroscopic effects (such as diffusion) through cumulative behavior. Let $\tilde{t}$ be the time scale of Equation (2) in the main text and t be the time scale of Equation (4) in the main text. The temporal scaling relation is $\tilde{t} = \frac{t}{\varepsilon^2}$, meaning one macroscopic time unit corresponds to $O(1/\varepsilon^2)$ microscopic transition events. The phenotypic transition velocity in the t-scale is $\frac{dx}{dt} = V(x)$, and we have:

$$\frac{dx}{d\tilde{t}} = \tilde{V}(x) = \varepsilon^2 \frac{dx}{dt} = \varepsilon^2 V(x).$$

Define $\rho_\varepsilon(x,t) = \tilde{\rho}\left(x, \frac{t}{\varepsilon^2}\right) = \tilde{\rho}(x,\tilde{t})$, and $g_\varepsilon(x,t) = \tilde{g}\left(x, \frac{t}{\varepsilon^2}\right) = \tilde{g}(x,\tilde{t})$. Then

$$\frac{\partial \rho_\varepsilon(x,t)}{\partial t} = \frac{1}{\varepsilon^2} \frac{\partial \tilde{\rho}(x,\tilde{t})}{\partial \tilde{t}}.$$

Let u be the macroscopic displacement vector per unit time in phenotype space. We assume an ε-scaling between microscopic and macroscopic displacements, leading to the relation $\tilde{u} = \varepsilon u$. This implies $x - y = \varepsilon u$, and thus $u = \frac{x-y}{\varepsilon}$.

The kernel function transforms as:

$$K_\varepsilon(y,u) = \varepsilon^d K(y,\varepsilon u) = \varepsilon^d K(y,\tilde{u}) = \varepsilon^d \, det\left(\tilde{B}(y)\right)^{-1/2} \mathcal{K}\left(\left|\tilde{B}(y)^{-1/2}\left(\tilde{u}-\tilde{V}(y)\right)\right|^2\right)$$

$$= det\left(\varepsilon^{-2}\tilde{B}(y)\right)^{-1/2} \mathcal{K}\left(\left|\tilde{B}(y)^{-1/2}\left(\varepsilon u-\varepsilon^2 V(y)\right)\right|^2\right)$$

$$:= det\left(B(y)\right)^{-1/2} \mathcal{K}\left(\left|B(y)^{-1/2}\left(u-\varepsilon V(y)\right)\right|^2\right),$$

where we define $\tilde{B}(y) = \varepsilon^2 B(y)$. The elements $B_{ij}(y)(i,j \in \{1,2,\dots,d\})$ are continuous and bounded, and since $\tilde{B}(y)$ is positive definite, so is $B(y)$.

Substituting the above into Equation (2) in the main text, we obtain:

$$\varepsilon^2 \frac{\partial \rho_\varepsilon(x,t)}{\partial t} + k_\varepsilon(x)\rho_\varepsilon(x,t)$$

$$= \frac{1}{\varepsilon^d}\int_{\mathbb{R}^d} K_\varepsilon\left(y,\frac{x-y}{\varepsilon}\right)\rho_\varepsilon(y,t)\ dy + \left(1-\frac{\int_{\mathbb{R}^d}\rho_\varepsilon(x,t)\ dx}{M}\right) g_\varepsilon(x,t)\rho_\varepsilon(x,t),$$

which is precisely the form of Equation (4) in the main text.

Appendix Text S2. Convergence of the scaled equation to the limit equation.

Some assumptions and properties

We assume that $V(x)$ is smooth and bounded. The total rescaled growth is constrained: $\int_{\mathbb{R}^d} |g_\varepsilon(x,t)|dx \le M$ for each t. We next set

$$K_\varepsilon(y,u) = \det B^{-1/2}\, \mathcal{K}\left(\left|B(y)^{-1/2}\big(u - \varepsilon V(y)\big)\right|^2\right).$$

Here, we take $\mathcal{K}(|w|^2) = \frac{1}{(2\pi)^{d/2}} e^{-|w|^2/2}$, which indeed satisfies condition (S1).

We now verify that the transformed kernel K_ε satisfies the following conditions for applying Theorem.

Lemma With the help of condition (S1) we can prove:

$$k_\varepsilon(y) = \int_{\mathbb{R}^d} K_\varepsilon(y,u)du = \int_{\mathbb{R}^d} \mathcal{K}(|\eta|^2)d\eta = 1,$$

$$\int_{\mathbb{R}^d} uK_\varepsilon(y,u)du = \varepsilon V(y), \quad \int_{\mathbb{R}^d} u_i\, u_j K_\varepsilon(y,u)du = B_{ij}(y) + \varepsilon^2 V_i(y)V_j(y), \tag{S2}$$

$$\int_{\mathbb{R}^d} |u|^3 K_\varepsilon(y,u)du \in L^\infty(\mathbb{R}^d) \tag{S3}$$

Proof of the Lemma Using the conditions in (S1) and applying the change of variables:

$$\eta = B(y)^{-1/2} \cdot \big(u - \varepsilon V(y)\big), \quad d\eta = \big(det\, B\,(y)\big)^{-1/2}\, du,$$

we obtain:

$$\int_{\mathbb{R}^d} \big(u - \varepsilon V(y)\big) K_\varepsilon(y,u)\ du = \int_{\mathbb{R}^d} B(y)^{1/2} \cdot \eta\, \mathcal{K}(|\eta|^2)\, d\eta = 0.$$

Thus:

$$\int_{\mathbb{R}^d} u\, K_\varepsilon(y,u)\, du = \varepsilon V(y) \int_{\mathbb{R}^d} K_\varepsilon(y,u)\ du + \int_{\mathbb{R}^d} \big(u - \varepsilon V(y)\big) K_\varepsilon(y,u)\ du = \varepsilon V(y),$$

where $V(y) = \frac{1}{\varepsilon^2} \tilde{V}(y)$ is smooth and bounded.

Furthermore:

$$\begin{aligned}
\int_{\mathbb{R}^d} u_i u_j K_\varepsilon(y,u)\, du &= \int_{\mathbb{R}^d} \big(u_i - \varepsilon V_i(y)\big)\left(u_j - \varepsilon V_j(y)\right) K_\varepsilon(y,u)\ du + \varepsilon^2 V_i(y)V_j(y) \\
&= \int_{\mathbb{R}^d} (B(y)^{1/2} \cdot \eta)_i (B(y)^{1/2} \cdot \eta)_j\ \mathcal{K}(|\eta|^2)\, d\eta + \varepsilon^2 V_i(y)V_j(y) \\
&= \sum_{k,l=1}^{d} \int_{\mathbb{R}^d} \big(B(y)_{ik}^{1/2}\eta_k\big)\big(B(y)_{lj}^{1/2}\eta_l\big) \mathcal{K}(|\eta|^2)\ d\eta + \varepsilon^2 V_i(y)V_j(y) \\
&= \sum_{k=1}^{d} B(y)_{ik}^{1/2} B(y)_{kj}^{1/2} + \varepsilon^2 V_i(y)V_j(y) = B_{ij}(y) + \varepsilon^2 V_i(y)V_j(y).
\end{aligned}$$

The matrix $\left[B_{ij}(y) + \varepsilon^2 V_i(y)V_j(y)\right]_{ij}$ remains positive definite, and its elements are continuous and bounded. Since $\mathcal{K}$ is a Gaussian density function, $K_\varepsilon(y,u)$ exhibits

exponential decay, ensuring that $\int_{\mathbb{R}^d} |u|^3 K_\varepsilon(y,u)\ du$ is bounded.

In summary, we have shown that the transformed kernel K_ε satisfies the conditions in the Lemma.

Similarly, we can prove the following conclusion. For

$$K(y,u) = \det B^{-1/2}\, \mathcal{K}\left(\left|B(y)^{-1/2}\big(u - V(y)\big)\right|^2\right),$$

where $\mathcal{K}(|w|^2) = \frac{1}{(2\pi)^{d/2}} e^{-|w|^2/2}$, we have:

$$k(y) = \int_{\mathbb{R}^d} K(y,u) du = \int_{\mathbb{R}^d} \mathcal{K}(|\eta|^2) d\eta = 1,$$

$$\int_{\mathbb{R}^d} uK(y,u) du = V(y), \quad \int_{\mathbb{R}^d} u_i u_j K(y,u) du = B_{ij}(y) + V_i(y) V_j(y),$$

$$\int_{\mathbb{R}^d} |u|^3 K(y,u) du \in L^\infty(\mathbb{R}^d).$$

Convergence relations of the equations

Before proving the convergence relations of the equations in the main text, we present the following conclusion, so that by substituting $(B_{ij}) = 2D \cdot (\delta_{ij})$ we can prove that Equation (4) in the main text converges in the weak sense to Equation (7) in the main text:

Theorem Assume that the initial values of both equations are $\rho^0(x)$, that for each x, the limit $\lim_{\tilde{t}\to\infty} \tilde{t}\tilde{g}(x,\tilde{t})$ exists, and that Equation (4) satisfies conditions (3) and (5) in the main text. Then, as ε approaches 0, the solution of the equation

$$\begin{aligned}\frac{\partial \rho_\varepsilon}{\partial t} = -\frac{1}{\varepsilon^2}\left[k(x)\rho_\varepsilon(x,t) - \int_{\mathbb{R}^d} \frac{1}{\varepsilon^d} K_\varepsilon\left(y, \frac{x-y}{\varepsilon}\right) \rho_\varepsilon(x,t)\ dy\right] \\ + \frac{1}{\varepsilon^2}\left(1 - \frac{\int_{\mathbb{R}^d} \rho_\varepsilon(x,t)\ dx}{M}\right) g_\varepsilon(x,t) \rho_\varepsilon(x,t)\end{aligned} \tag{S4}$$

converges in the weak sense to the solution of the equation:

$$\partial_t \rho + \nabla_x \cdot (V\rho) = \frac{1}{2}\sum_{i,j=1}^{d} \frac{\partial^2}{\partial_{x_i}\partial_{x_j}}\left[B_{ij}(x)\rho(x,t)\right] + \left(1 - \frac{\int_{\mathbb{R}^d} \rho(x,t)\ dx}{M}\right) g\rho. \tag{S5}$$

The meaning of weak sense convergence, for the solution of Equation (S4) to the solution of the limit equation (S5), is as follows: there exists a subsequence of the solution with respect to ε such that, after multiplying both sides of (S4) by a test function and integrating, the limit as $\varepsilon \to 0$ can be taken; this limit equals the result of the same operation applied to the limit Equation (S5).

Before proceeding with the proof, we first present a lemma (Brezis 2010) that will be used.

Lemma (Corollary 3.30 in (Brezis 2010))

Let X be a separable Banach space, $\{f_n\} \subset X^*$ be a bounded sequence, then $\{f_n\}$ has a

weakly * convergent subsequence.

We restrict the solutions of Equation (S4) and Equation (S5) to the L^2 space. Combining the reflexive property of the L^2 space, ρ_ε can extract a subsequence, still denoted as ρ_ε, and there exists ρ such that:

$$\rho_\varepsilon \to \rho \quad \text{weakly in } L^2(\mathbb{R}^d \times \mathbb{R}^+).$$

This means that for $\Phi \in L^2(\mathbb{R}^+ \times \mathbb{R}^d)$,

$$\int_0^\infty \int_{\mathbb{R}^d} \rho_\varepsilon(x,t)\Phi(t,x)dxdt \to \int_0^\infty \int_{\mathbb{R}^d} \rho(x,t)\Phi(t,x)dxdt,$$

i.e., there exists a subsequence of ρ_ε that weakly converges to ρ in $L^2(\mathbb{R}^d \times \mathbb{R}^+)$.

Proof of Theorem We assume that the solution of (S4) exists in the space L^2.

Change the variable in equation (S4) from y to $u = \frac{x-y}{\varepsilon}$. We obtain the following formula:

$$\frac{\partial}{\partial t}\rho_\varepsilon(x,t) + \int_{\mathbb{R}^d} \frac{1}{\varepsilon^2}[K_\varepsilon(x,u)\rho_\varepsilon(x,t) - K_\varepsilon(x-\varepsilon u,u)\rho_\varepsilon(x-\varepsilon u,t)]du$$
$$= \frac{1}{\varepsilon^2}\left(1 - \frac{\int_{\mathbb{R}^d} \rho_\varepsilon(x,t)\ dx}{M}\right) g_\varepsilon(x,t)\rho_\varepsilon(x,t).$$

We introduce a test function $\Phi(t,x) \in C^3(\mathbb{R}^+ \times \mathbb{R}^d)$ with compact support, i.e., for some $T, R > 0$, $\Phi(t,x) = 0$ when $t \geq T$ or $|x| > R$. Using integration by parts and Fubini's theorem, we obtain:

$$-\int_0^T \int_{\mathbb{R}^d} \frac{\partial}{\partial t}\Phi(t,x)\rho_\varepsilon(x,t)dxdt - \int_{\mathbb{R}^d} \Phi(0,x)\rho^0(x)dx$$
$$= \int_0^T \int_{\mathbb{R}^d} \rho_\varepsilon(x,t) \int_{\mathbb{R}^d} K_\varepsilon(x,u) \frac{\Phi(t,x+\varepsilon u) - \Phi(t,x)}{\varepsilon^2} dxdudt$$
$$+ \int_0^T \int_{\mathbb{R}^d} \frac{1}{\varepsilon^2}\left(1 - \frac{\int_{\mathbb{R}^d} \rho_\varepsilon(x,t)\ dx}{M}\right) g_\varepsilon(x,t)\rho_\varepsilon(x,t)\Phi(t,x)dxdt.$$

Now we handle the integral on the right:

$$I_\varepsilon = \int_0^T \int_{\mathbb{R}^d} \rho_\varepsilon(x,t) \int_{\mathbb{R}^d} K_\varepsilon(x,u) \frac{\Phi(t,x+\varepsilon u) - \Phi(t,x)}{\varepsilon^2} dxdudt,$$
$$J_\varepsilon = \int_0^T \int_{\mathbb{R}^d} \frac{1}{\varepsilon^2}\left(1 - \frac{\int_{\mathbb{R}^d} \rho_\varepsilon(x,t)\ dx}{M}\right) g_\varepsilon(x,t)\rho_\varepsilon(x,t)\Phi(t,x)dxdt$$

Perform a third-order Taylor expansion of $\Phi(t, x + \varepsilon u)$:

$$\Phi(t,x+\varepsilon u) = \Phi(t,x) + \varepsilon u \nabla_x \Phi(t,x) + \frac{\varepsilon^2}{2} u^T D_x^2 \Phi(t,x) u + O(\varepsilon^3 |u|^3),$$

Therefore, due to (S3), $\int_{\mathbb{R}^d} |u|^3 K_\varepsilon(y,u)du$ is almost everywhere bounded, we have

$$I_\varepsilon = \frac{1}{\varepsilon^2}\int_0^T \int_{\mathbb{R}^d} \rho_\varepsilon(x,t) \int_{\mathbb{R}^d} K_\varepsilon(x,u) \left[\varepsilon u \cdot \nabla_x \Phi(t,x) + \frac{\varepsilon^2}{2}\sum_{i,j=1}^d u_i u_j D_{x_i x_j}^2 \Phi(t,x)\right] dudx\, dt + O(\varepsilon)$$

$$= \frac{1}{\varepsilon}\int_0^T \int_{\mathbb{R}^d} \rho_\varepsilon(x,t) \int_{\mathbb{R}^d} K_\varepsilon(x,u)u\,du \cdot \nabla_x \Phi(t,x)dxdt$$

$$+ \frac{1}{2}\int_0^T \int_{\mathbb{R}^d} \rho_\varepsilon(x,t) \sum_{i,j=1}^{d} \left(\int_{\mathbb{R}^d} K_\varepsilon(x,u)u_i u_j du\right) D^2_{x_i x_j}\Phi(t,x)dxdt + O(\varepsilon)$$

$$= I_\varepsilon^{(1)} + I_\varepsilon^{(2)} + O(\varepsilon)$$

With the help of (S2) and the boundedness of $V(x)$, we can write:

$$I_\varepsilon^{(1)} = \frac{1}{\varepsilon}\int_0^T \int_{\mathbb{R}^d} \rho_\varepsilon(x,t) \int_{\mathbb{R}^d} K_\varepsilon(x,u)u\,du \cdot \nabla_x \Phi(t,x)dxdt = \int_0^T \int_{\mathbb{R}^d} \rho_\varepsilon(x,t)V(x) \cdot \nabla_x \Phi(t,x)dxdt$$

$$I_\varepsilon^{(2)} = \frac{1}{2}\int_0^T \int_{\mathbb{R}^d} \rho_\varepsilon(x,t) \sum_{i,j=1}^{d} \left[\int_{\mathbb{R}^d} K_\varepsilon(x,u)u_i u_j du\right] D^2_{x_i x_j}\Phi(t,x)dxdt$$

$$= \frac{1}{2}\int_0^T \int_{\mathbb{R}^d} \rho_\varepsilon(x,t) \sum_{i,j=1}^{d} \left[B_{ij}(x) + \varepsilon^2 V_i(x)V_j(x)\right] D^2_{x_i x_j}\Phi(t,x)dxdt$$

$$= \frac{1}{2}\int_0^T \int_{\mathbb{R}^d} \rho_\varepsilon(x,t) \sum_{i,j=1}^{d} B_{ij}(x) D^2_{x_i x_j}\Phi(t,x)dxdt + O(\varepsilon^2).$$

So when $\varepsilon \to 0$, we have

$$I_\varepsilon = \int_0^T \int_{\mathbb{R}^d} \rho_\varepsilon(x,t) \int_{\mathbb{R}^d} K_\varepsilon(x,u) \frac{\Phi(t,x+\varepsilon u) - \Phi(t,x)}{\varepsilon^2} dudxdt$$

$$= \int_0^T \int_{\mathbb{R}^d} \rho_\varepsilon(x,t) \left[V(x) \cdot \nabla_x \Phi(t,x) + \frac{1}{2} B_{ij}(x) D^2_{x_i x_j}\Phi(t,x)\right] dxdt + O(\varepsilon).$$

Since we assume $\lim_{\tilde{t}\to\infty} \tilde{t}\tilde{g}(x,\tilde{t})$ exists for each (x,t), we define

$$g(x,t) := \lim_{\varepsilon\to 0} \frac{1}{\varepsilon^2} g_\varepsilon(x,t) = \lim_{\varepsilon\to 0} \frac{1}{\varepsilon^2} \tilde{g}\left(x, \frac{t}{\varepsilon^2}\right) = \frac{1}{t} \lim_{s\to\infty} s\tilde{g}(x,s)$$

By the Lebesgue dominated convergence theorem, it is easy to prove:

$$J_\varepsilon = \int_0^T \int_{\mathbb{R}^d} \left(1 - \frac{\int_{\mathbb{R}^d} \rho_\varepsilon(x,t)\,dx}{M}\right) g(x,t)\rho_\varepsilon(x,t)\Phi(t,x)dxdt + O(\varepsilon).$$

That is, when $\varepsilon \to 0$,

$$-\int_0^T \int_{\mathbb{R}^d} \frac{\partial}{\partial t}\Phi(t,x)\rho_\varepsilon(x,t)dxdt - \int_0^T \int_{\mathbb{R}^d} \Phi(0,x)\rho^0(x)dxdt$$

$$= \int_0^T \int_{\mathbb{R}^d} \rho_\varepsilon(x,t) \left[V(x) \cdot \nabla_x \Phi(t,x) + \frac{1}{2} B_{ij}(x) D^2_{ij}\Phi(t,x)\right] dxdt$$

$$+ \int_0^T \int_{\mathbb{R}^d} \left(1 - \frac{\int_{\mathbb{R}^d} \rho_\varepsilon(x,t)\ dx}{M}\right) g(x,t)\rho_\varepsilon(x,t)\Phi(t,x)dxdt + O(\varepsilon).$$

In fact, combining the density of $\Phi(t,x) \in C^3(\mathbb{R}^+ \times \mathbb{R}^d)$ in $L^2(\mathbb{R}^+ \times \mathbb{R}^d)$, as long as we show that there exists a weakly convergent subsequence of ρ_ε, and define the weak limit as ρ, then the conclusion is proved.

By assumption, ρ_ε is bounded in L^2, by the preceding Lemma (Brezis 2010), we can extract

a weakly convergent subsequence from ρ_ε in L^2, and assume the weak limit is ρ. Therefore, we easily prove, when $\varepsilon \to 0$:

$$\int_0^T \int_{\mathbb{R}^d} \rho_\varepsilon(x,t) \left[V(x) \cdot \nabla_x \Phi(t,x) + \frac{1}{2} B_{ij}(x) D_{ij}^2 \Phi(t,x)\right] dxdt$$

$$\to \int_0^T \int_{\mathbb{R}^d} \rho(x,t) \left[V(x) \cdot \nabla_x \Phi(t,x) + \frac{1}{2} B_{ij}(x) D_{ij}^2 \Phi(t,x)\right] dxdt,$$

$$\int_0^T \int_{\mathbb{R}^d} \left(1 - \frac{\int_{\mathbb{R}^d} \rho_\varepsilon(x,t)\ dx}{M}\right) g(x,t) \rho_\varepsilon(x,t) \Phi(t,x) dxdt$$

$$\to \int_0^T \int_{\mathbb{R}^d} \left(1 - \frac{\int_{\mathbb{R}^d} \rho(x,t)\ dx}{M}\right) g(x,t) \rho(x,t) \Phi(t,x) dxdt$$

So, we have proven that from ρ_ε we can extract a subsequence that weakly converges to ρ in $L^2\left(\mathbb{R}^d \times (0,T)\right)$, and ρ satisfies Equation (S5) in the weak sense, i.e., the solution of Equation (S4) converges in the weak sense to the solution of Equation (S5).

Appendix Text S3. Calculating probabilities of progenitor cells differentiating to the Neu fate in mouse hematopoiesis.

Using the unique trajectory inferred by GRNvelo for each progenitor cell at day 2 within the mouse hematopoiesis dataset, we computed the probability of its descendants differentiating into the Neu fate by day 6. The calculation follows the procedure below. First, using the cell fate annotations provided in reference (Weinreb, et al. 2020), we construct kernel density-estimated fate distributions for Neu and Mo cells at day 6 in the two-dimensional SPRING space (Equations (S6) and (S7), where $\sigma = 0.025$).

$$\rho_6^{\mathrm{Neu}}(x) = \frac{1}{N}\sum_{j=1}^{N} \frac{\exp\left(-\frac{1}{2}\left(x - d_{6,j}^{\mathrm{Neu}}\right)^T \Sigma^{-1}\left(x - d_{6,j}^{\mathrm{Neu}}\right)\right)}{\sqrt{(2\pi)^2|\Sigma|}}, \tag{S6}$$

$$\rho_6^{\mathrm{Mo}}(x) = \frac{1}{M}\sum_{j=1}^{M} \frac{\exp\left(-\frac{1}{2}\left(x - d_{6,j}^{\mathrm{Mo}}\right)^T \Sigma^{-1}\left(x - d_{6,j}^{\mathrm{Mo}}\right)\right)}{\sqrt{(2\pi)^2|\Sigma|}}. \tag{S7}$$

Then, for each cell $d_{2,j}$ at day 2, its state at day 6 (denoted as $\hat{d}_{6,j}$) is predicted by forward-integrating its inferred velocity v from day 2 to day 6.

$$\hat{d}_{6,j} = d_{2,\mathrm{j}} + \int_2^6 v(x(t), t)\, \mathrm{d}t.$$

Finally, the Neu fate probability of the j-th cell (NFP_j) is determined by calculating the relative likelihood of its predicted state under the Neu fate density relative to the total (Neu + Mo) fate density (Equation (S8)).

$$\mathrm{NFP}_j = \frac{\rho_6^{\mathrm{Neu}}(\hat{d}_{6,j})}{\rho_6^{\mathrm{Neu}}(\hat{d}_{6,j}) + \rho_6^{\mathrm{Mo}}(\hat{d}_{6,j})}. \tag{S8}$$

Appendix Text S4. Calculation of cellular cluster population proportions of CD8+ T cells.

To systematically analyze population dynamics during late-stage CD8+ T cell differentiation, we develop a density-based computational framework that operates through two integrated phases. First, individual cells are assigned to specific populations by evaluating their relative positions within each predefined population's density distribution in the transcriptional space. This assignment process employs Gaussian kernel density estimation with cluster-specific covariance matrices, where each cell is classified into the population for which it demonstrates the highest probability density value (Cell Population = *argmax*(population density values)).

The density of cells with phenotype x in population $\{d_j|_{j=1}^{N}\}$ is calculated as follows:

$$\rho(x) = \frac{1}{N}\sum_{j=1}^{N} \frac{\exp\left(-\frac{1}{2}\left(x-d_j\right)^T \Sigma^{-1}(x-d_j)\right)}{\sqrt{(2\pi)^2|\Sigma|}}.$$

Following single-cell classification, we calculate population proportions by aggregating assignments across all cells (Population Proportion = (Number of Cells Assigned to Population) / (Total Cells)), implementing several quality control measures including density thresholding to exclude low-confidence assignments and batch correction to enable cross-sample comparisons.

Reference

Brezis H. 2010. Functional Analysis, Sobolev Spaces and Partial Differential Equations: Springer New York.

Weinreb C, Rodriguez-Fraticelli A, Camargo FD, Klein AM. 2020. Lineage tracing on transcriptional landscapes links state to fate during differentiation. Science 367:eaaw3381.

Appendix Figures

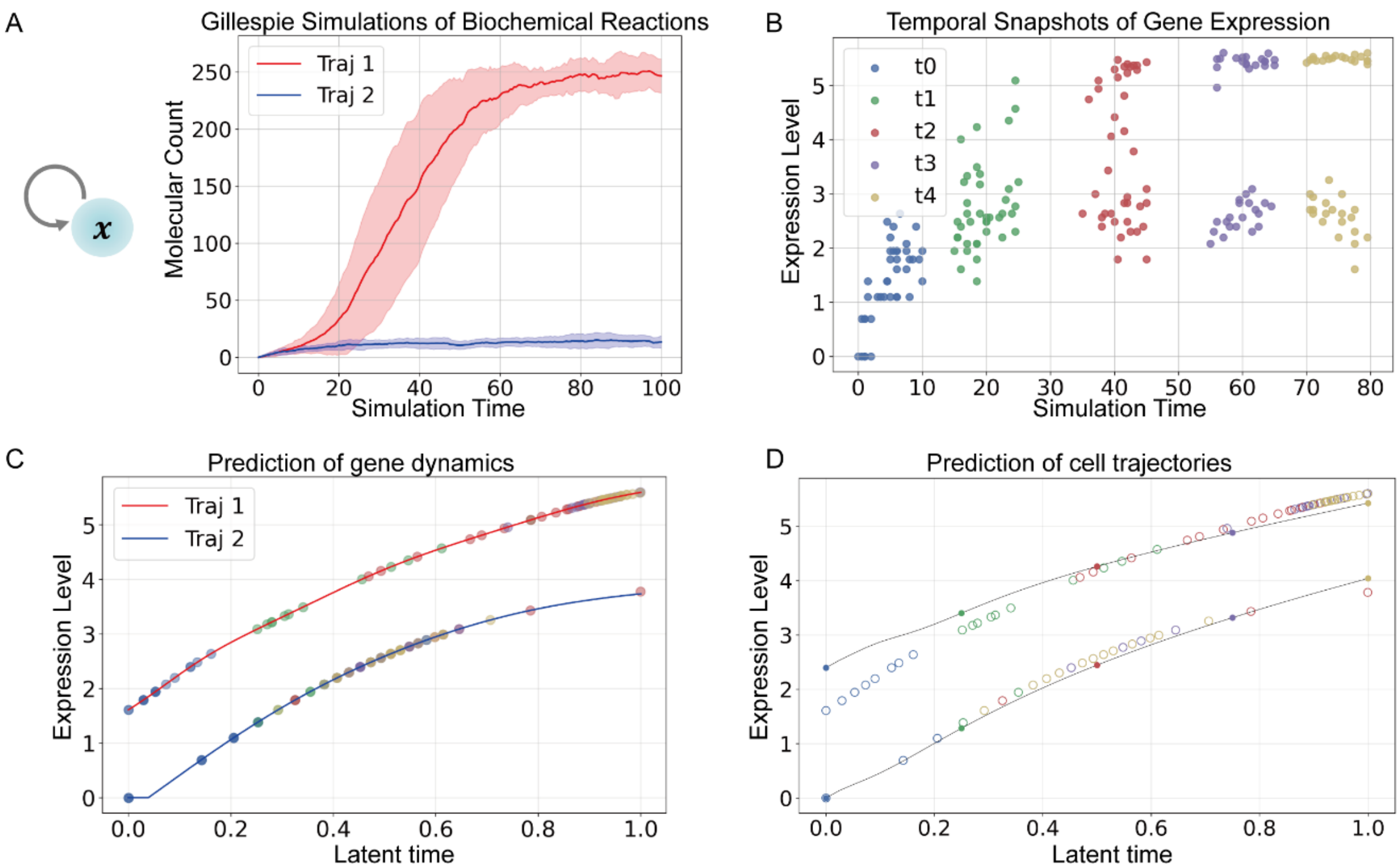

Appendix Figure S1. Gillespie-based simulation and model validation of a positive autoregulation system. (A) Topology of a single-gene autoregulatory circuit and two representative trajectory groups (mean ± SD) from Gillespie simulations. (B) Selected cells for model training and validation, annotated with five discrete time points. (C) Visualization of gene dynamics predicted by the model. (D) Predicted cell trajectories. For (B)-(D), the 'Expression Level' is defined as the log1p-transformed molecular count, i.e., $\boldsymbol{ln}(\textbf{Molecular Count} + \textbf{1})$.

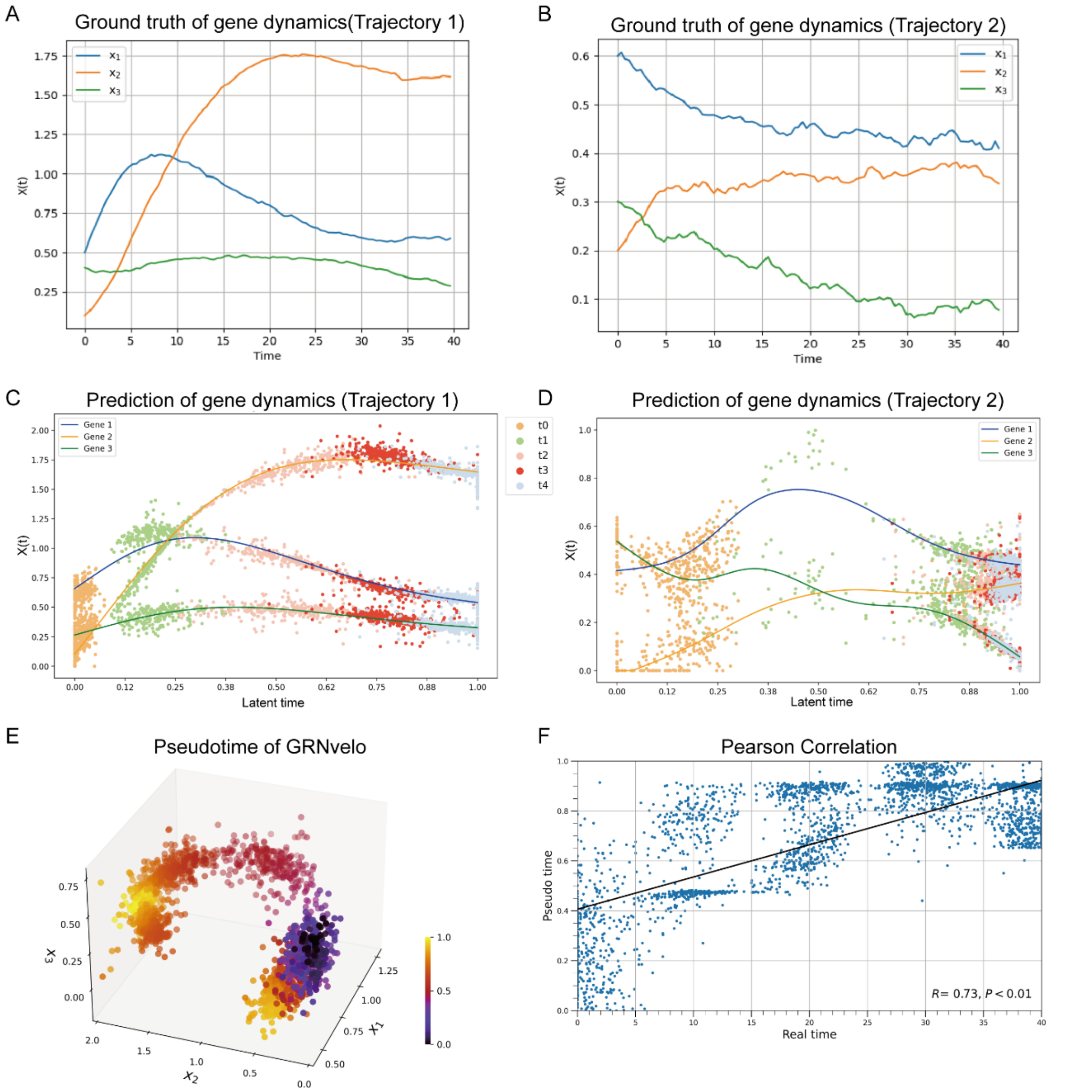

Appendix Figure S2. Supplementary information for 3-gene network. (A-B) The kinetic curves of synthetic data under the network of 3 genes. (C-D) The kinetic curve of model fitting. (E) Pseudotime. (F) Pearson correlation between conventional velocity-based pseudotime and real time.

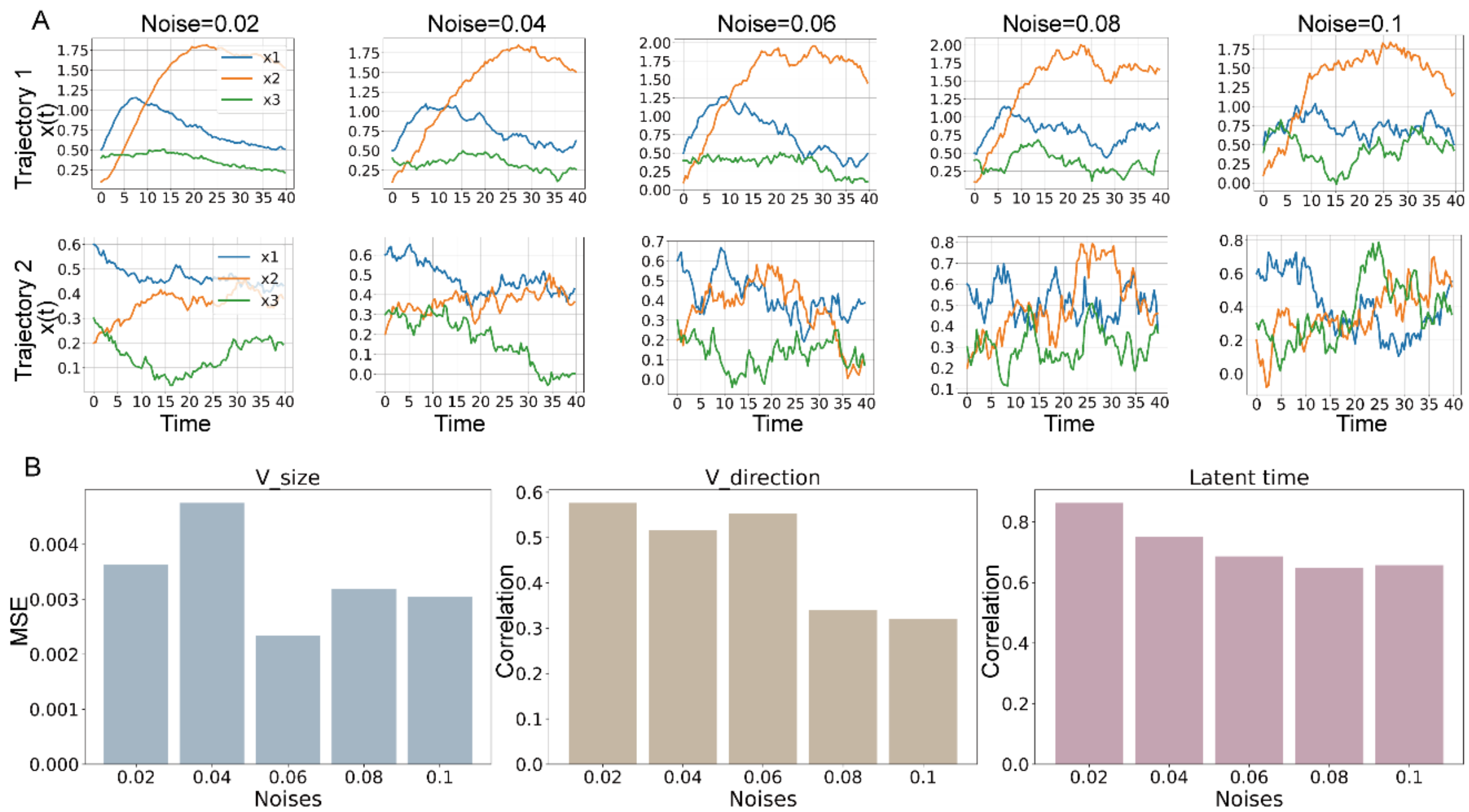

Appendix Figure S3. Performance of GRNvelo on simulated data under different noise levels. (A) Examples of gene dynamics for simulated data under different noise levels (noise standard deviation = 0.02, 0.04, 0.06, 0.08, 0.1). (B) Performance evaluation of GRNvelo on simulated data under different noise levels, including MSE of predicted velocity magnitudes (left panel), Pearson correlation of velocity directions (middle panel), and Pearson correlation of latent time (right panel).

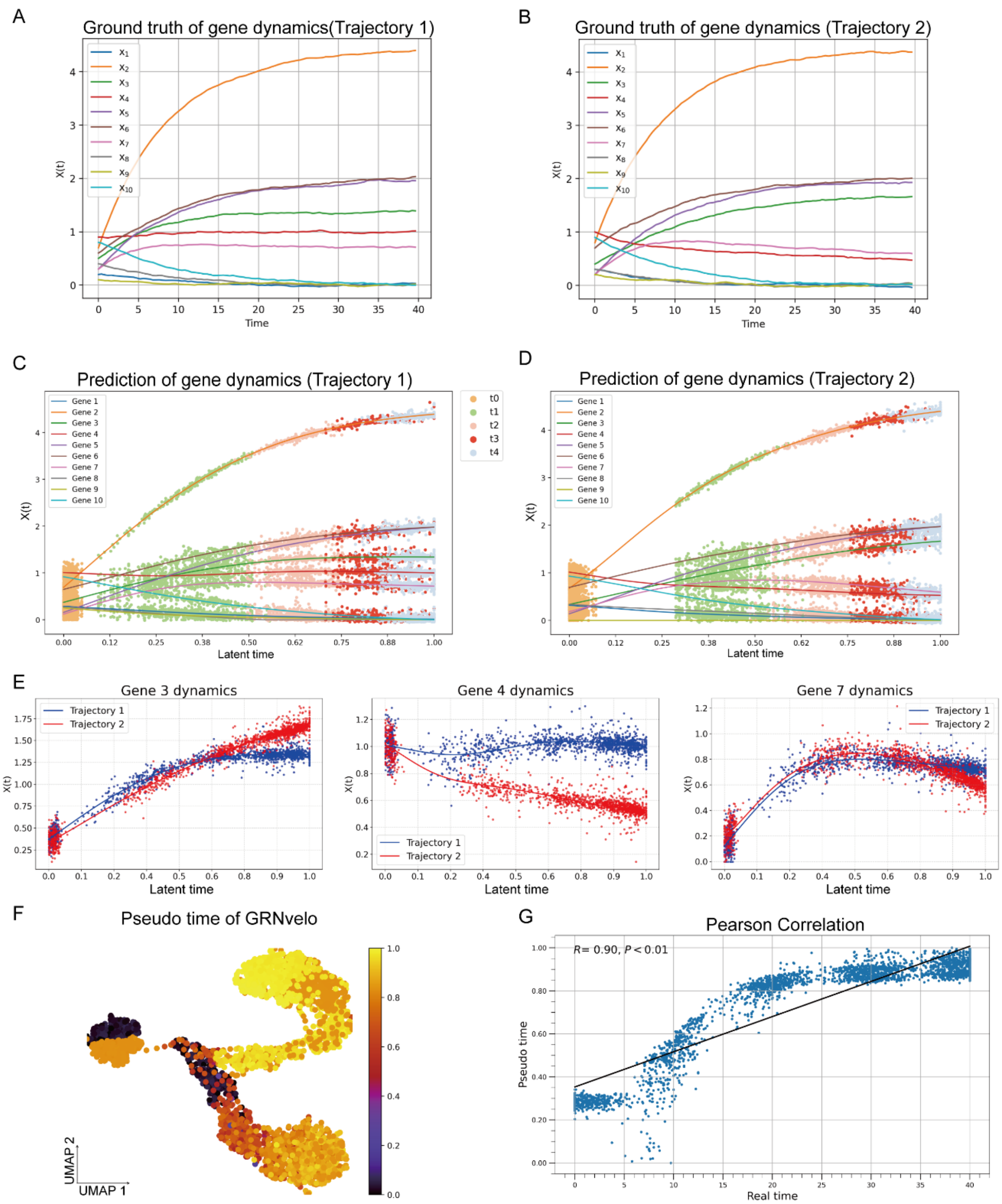

Appendix Figure S4. Supplementary information for 10-gene network. (A-B) The kinetic curves of synthetic data under the network of 10 genes. (C-D) The kinetic curves of model fitting for two branches, where the differential expression of (E) three key genes (Gene 3, Gene 4 and Gene 7) defines the bifurcation. (F) UMAP visualization of cells from different time points, showing the branching manifold driven by the dynamics in (C) and (D). (G) Pearson correlation between pseudotime and real time.

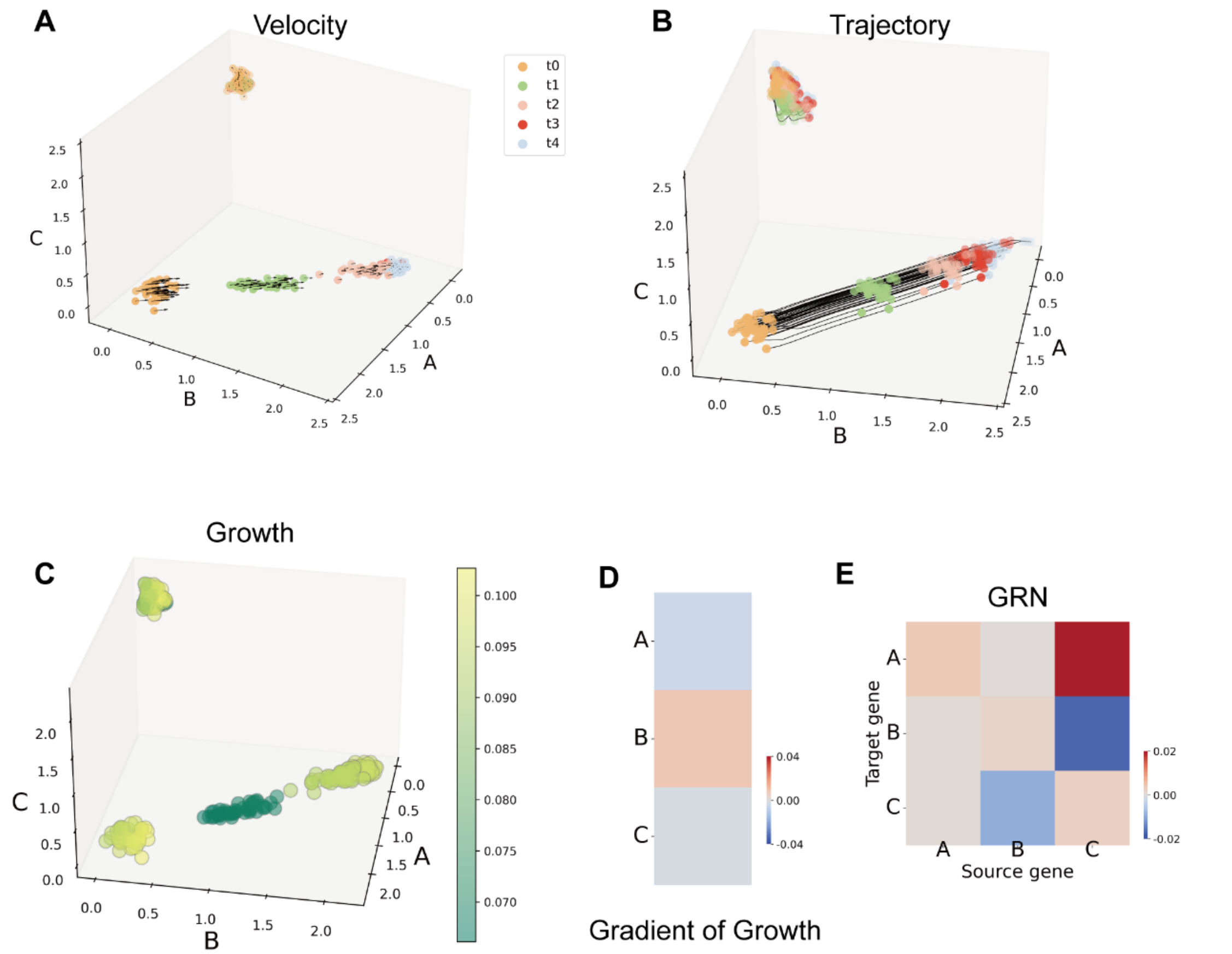

Appendix Figure S5. The prediction results of GRNvelo on the synthetic data in TIGON.
(A) Velocity. (B) Trajectory. (C) Growth. (D) Gradient of growth. (E) GRN.

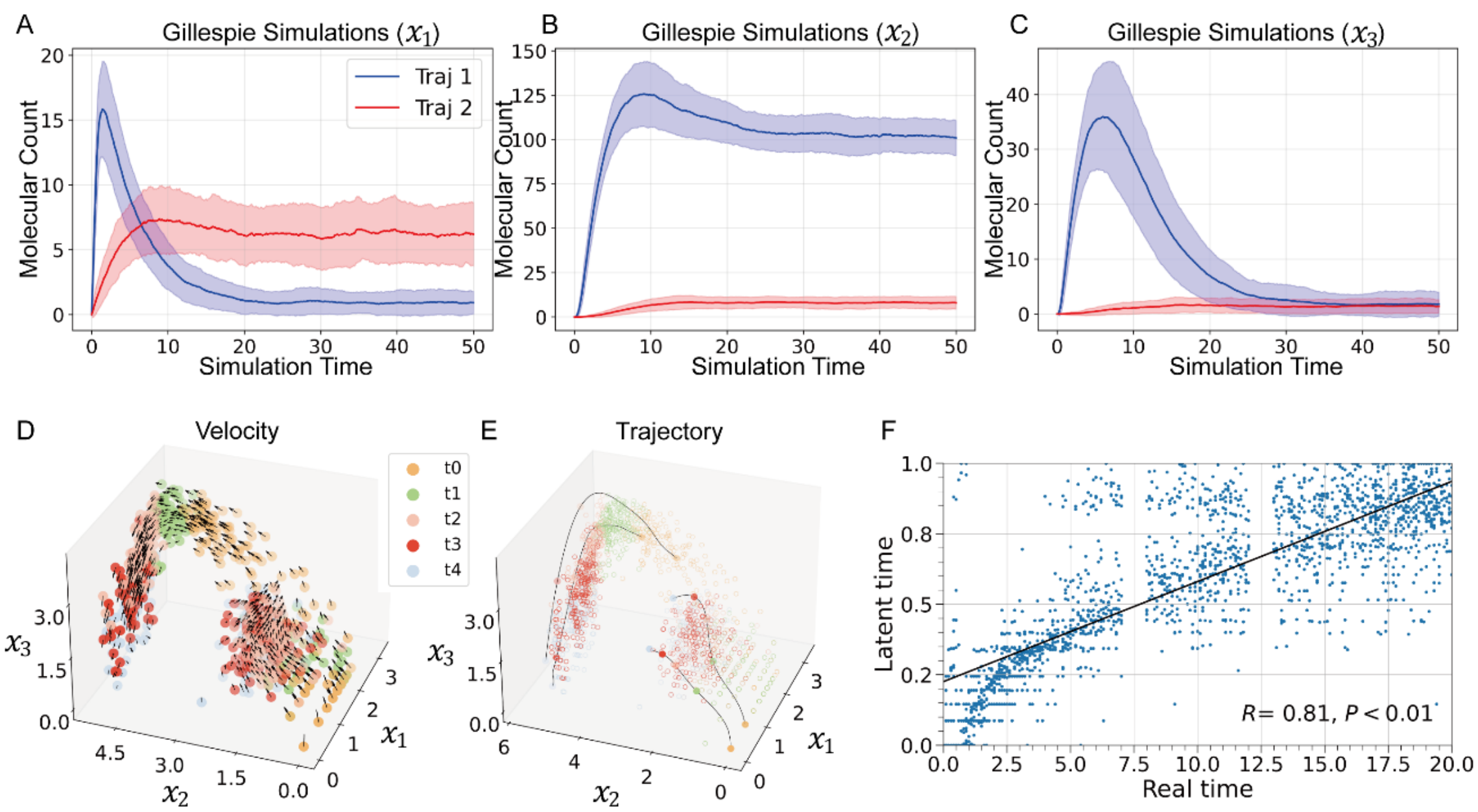

Appendix Figure S6. Gillespie-based simulation and model validation of a 3-gene regulatory network. (A-C) Two representative trajectory groups (mean ± SD) for gene (A) $\boldsymbol{x_1}$, (B) $\boldsymbol{x_2}$, (C) $\boldsymbol{x_3}$ from Gillespie simulations. (D) Predicted cell velocities. (E) Predicted cell trajectories. (F) Pearson correlation between predicted latent time and true simulation time.

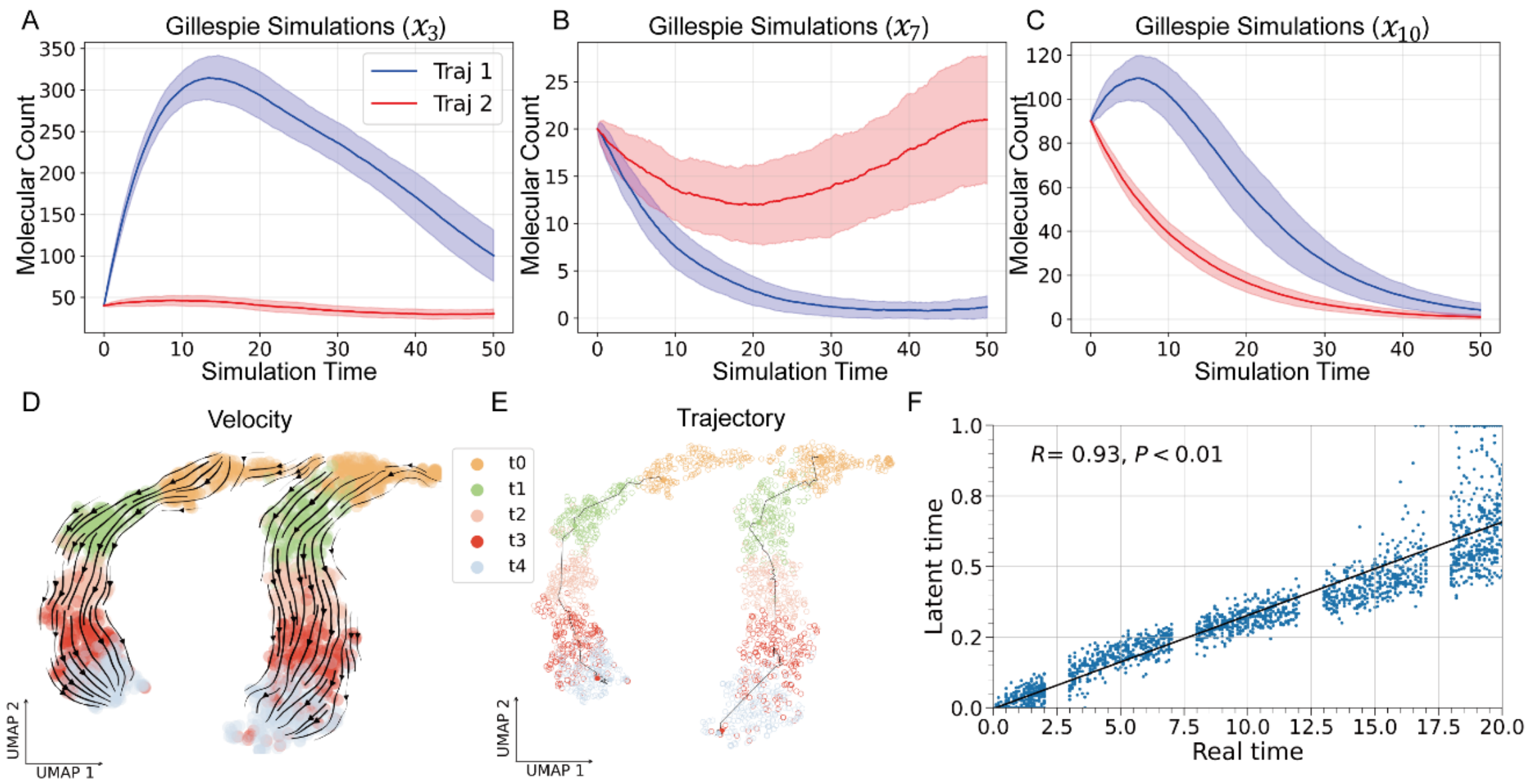

Appendix Figure S7. Gillespie-based simulation and model validation of a 10-gene regulatory network. (A-C) Two representative trajectory groups (mean ± SD) for gene (A) x_3, (B) x_7, (C) x_{10} , which shows large expression differences between the two trajectories. (D) Predicted cell velocities. (E) Predicted cell trajectories. (F) Pearson correlation between predicted latent time and true simulation time.

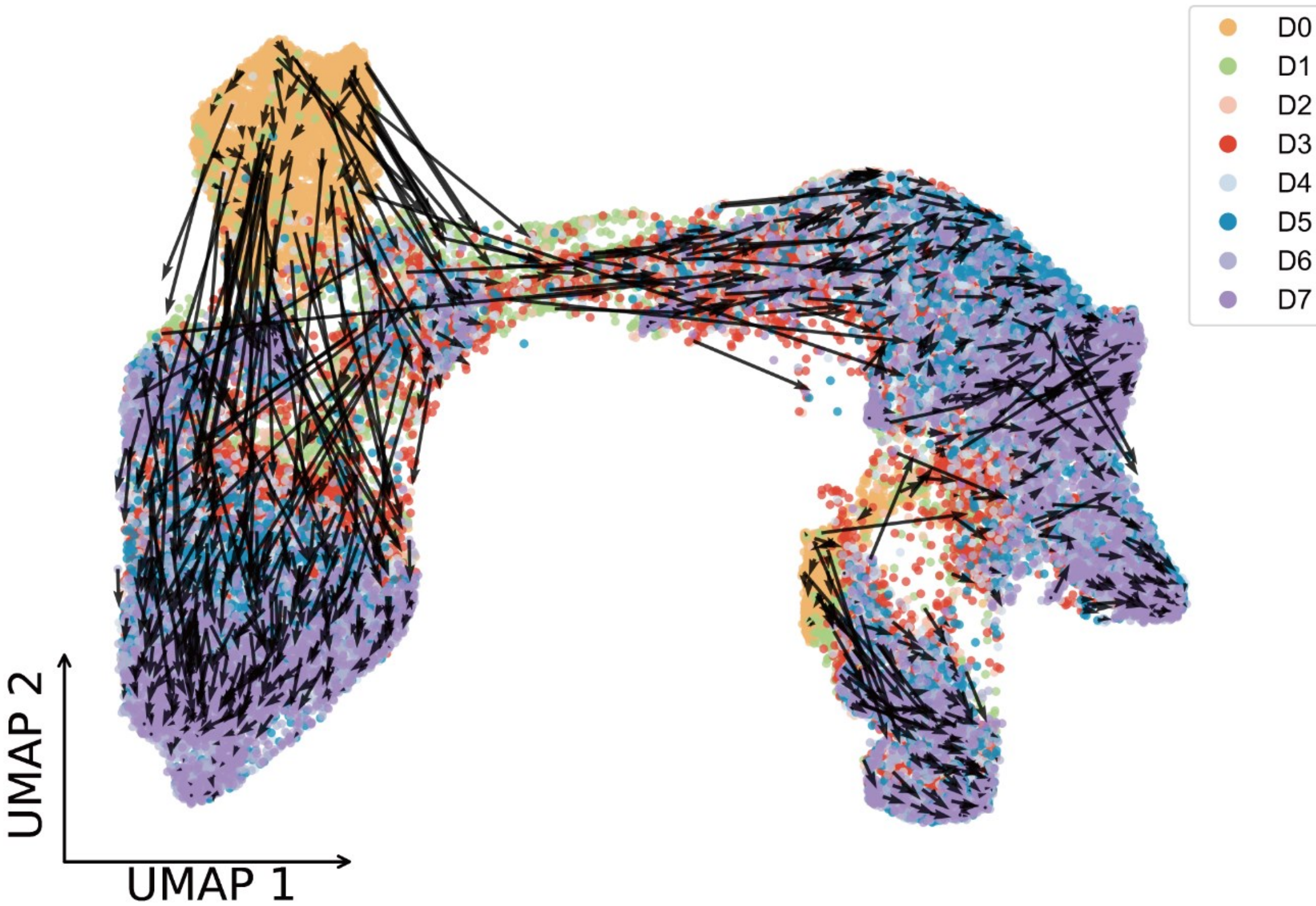

Appendix Figure S8. Visualization of the velocity predicted by GRNvelo in the pancreatic β-cell differentiation data. The arrows indicate the direction of cell state transition, and their length is proportional to the magnitude of velocity.

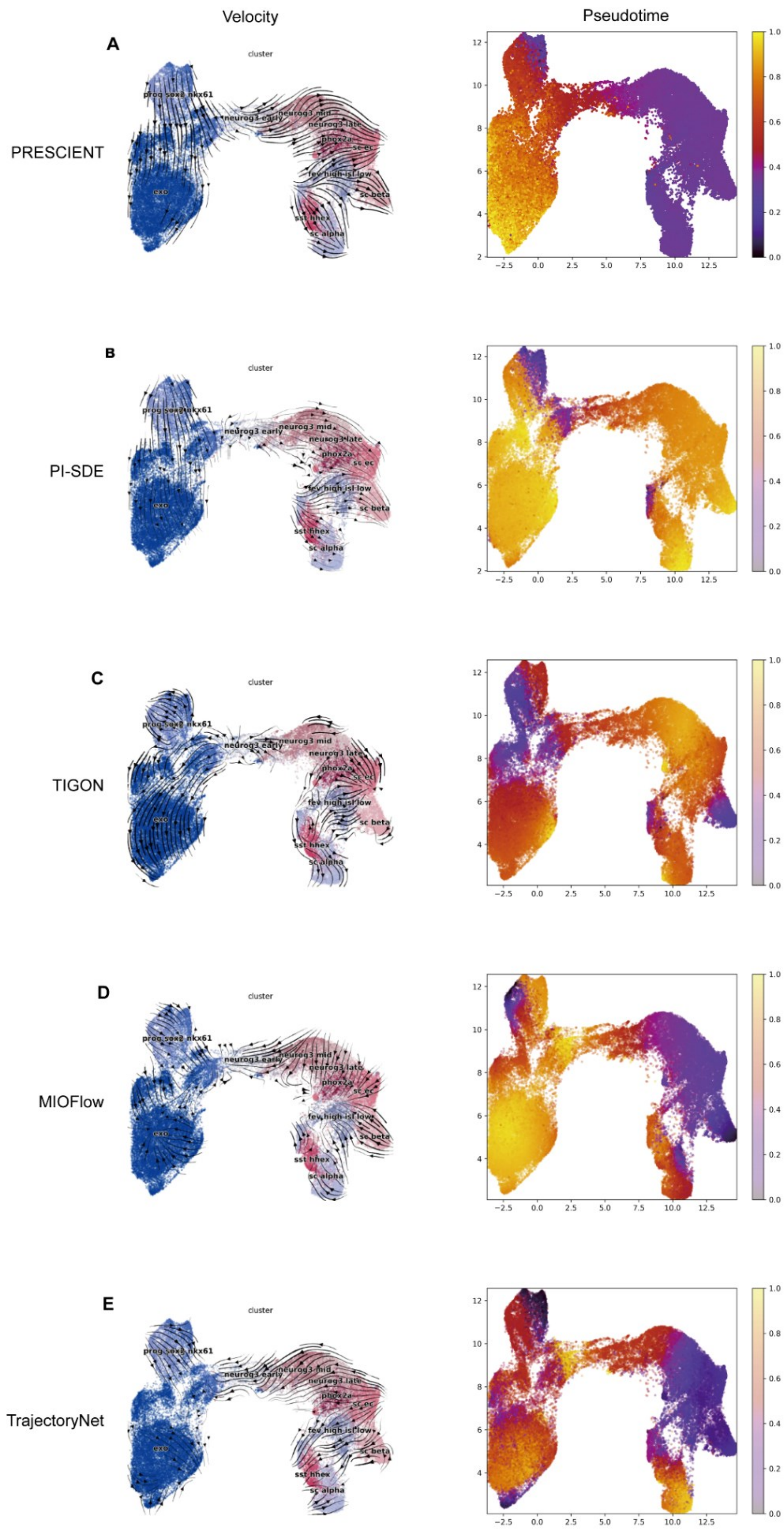

Velocity
Pseudotime
A
PRESCIENT
B
PI-SDE
C
TIGON
D
MIOFlow
E
TrajectoryNet
cluster

Appendix Figure S9. Visualization of the velocity and pseudotime predicted by the other 5 methods in the pancreatic β-cell differentiation data. (A) PRESCIENT. (B) PI-SDE. (C) TIGON. (D) MIOFlow. (E) TrajectoryNet.

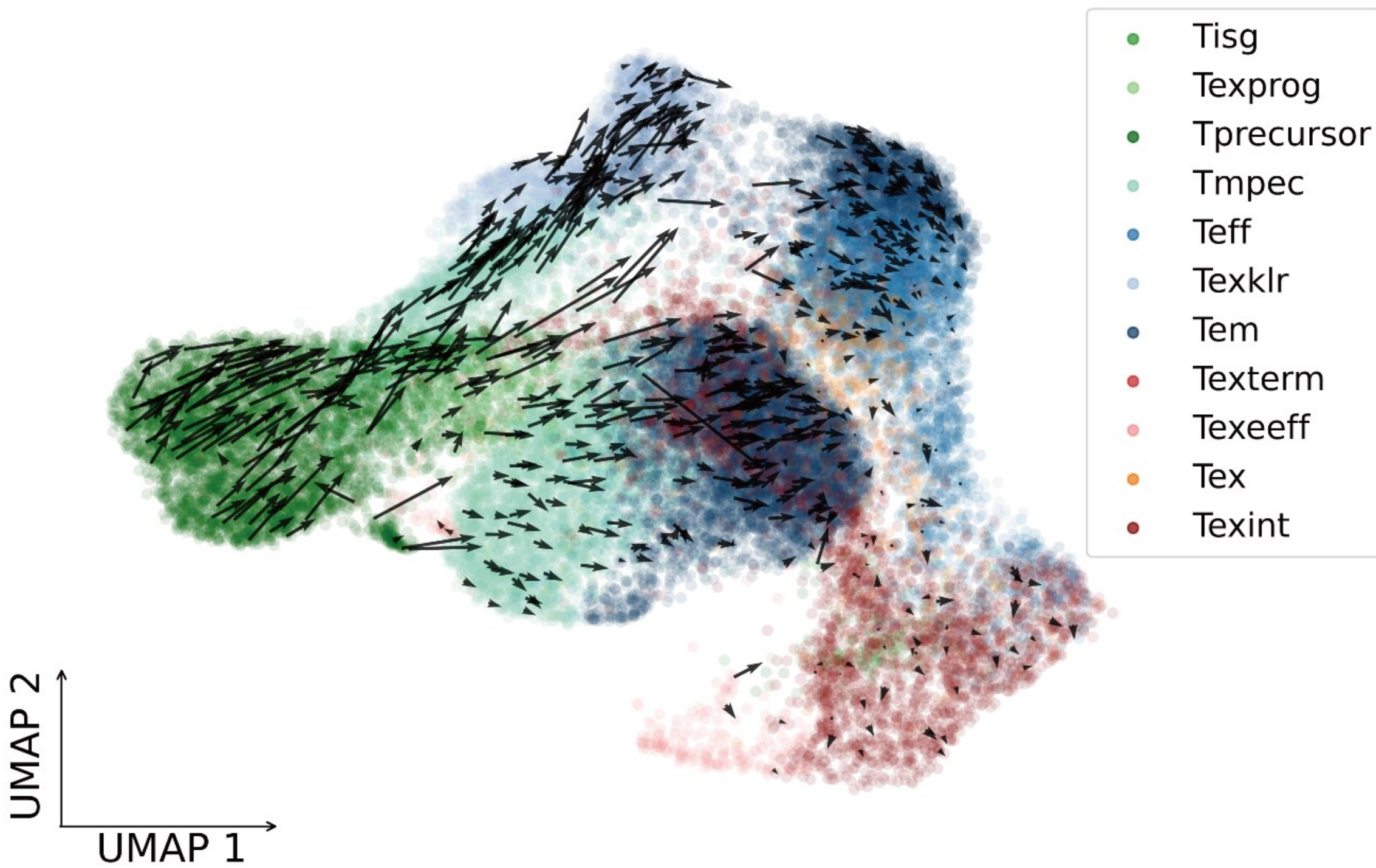

Appendix Figure S10. Visualization of the velocity predicted by GRNvelo in the acute LCMV infection data. The arrows indicate the direction of cell state transition, and their length is proportional to the magnitude of velocity.

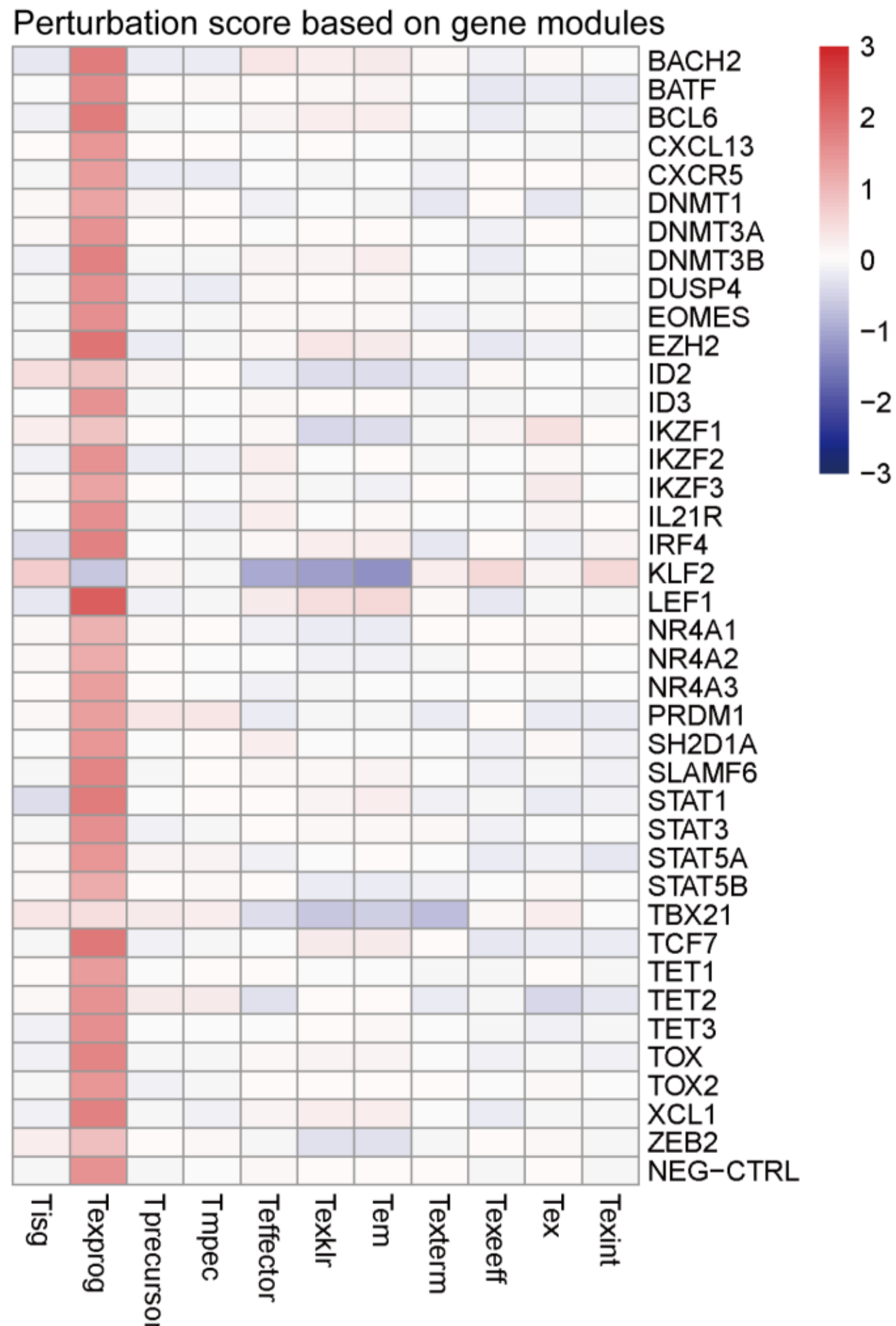

Appendix Figure S11. The ground truth for the enrichment of cluster gene modules in the acute LCMV infection data on D8. Each column represents a distinct gene module, and each row represents a gene. The color intensity in each tile corresponds to the z-score of enrichment.

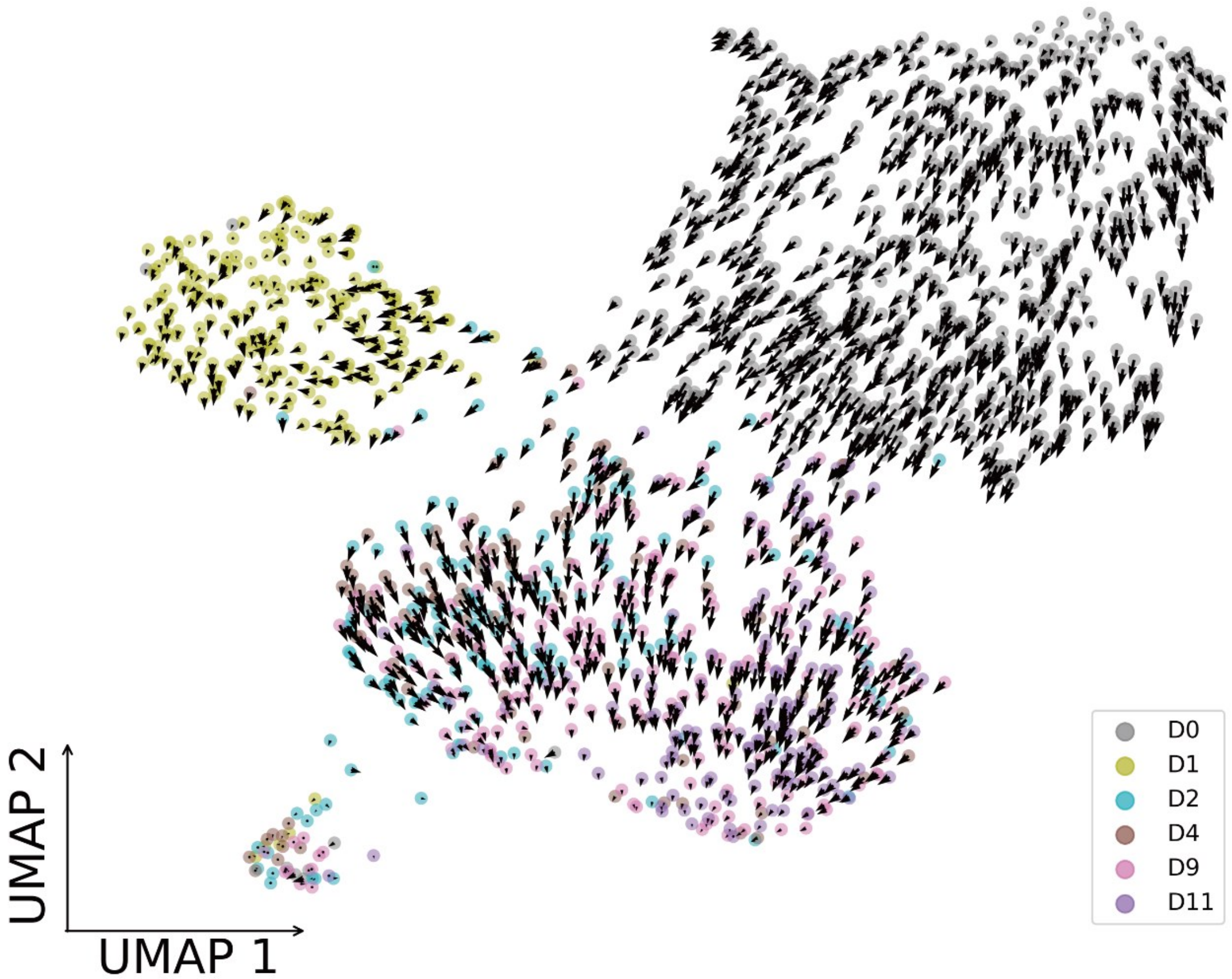

Appendix Figure S12. Visualization of the velocity predicted by GRNvelo in the drug-resistant PC9 tumor cells. The arrows indicate the direction of cell state transition, and their length is proportional to the magnitude of velocity.

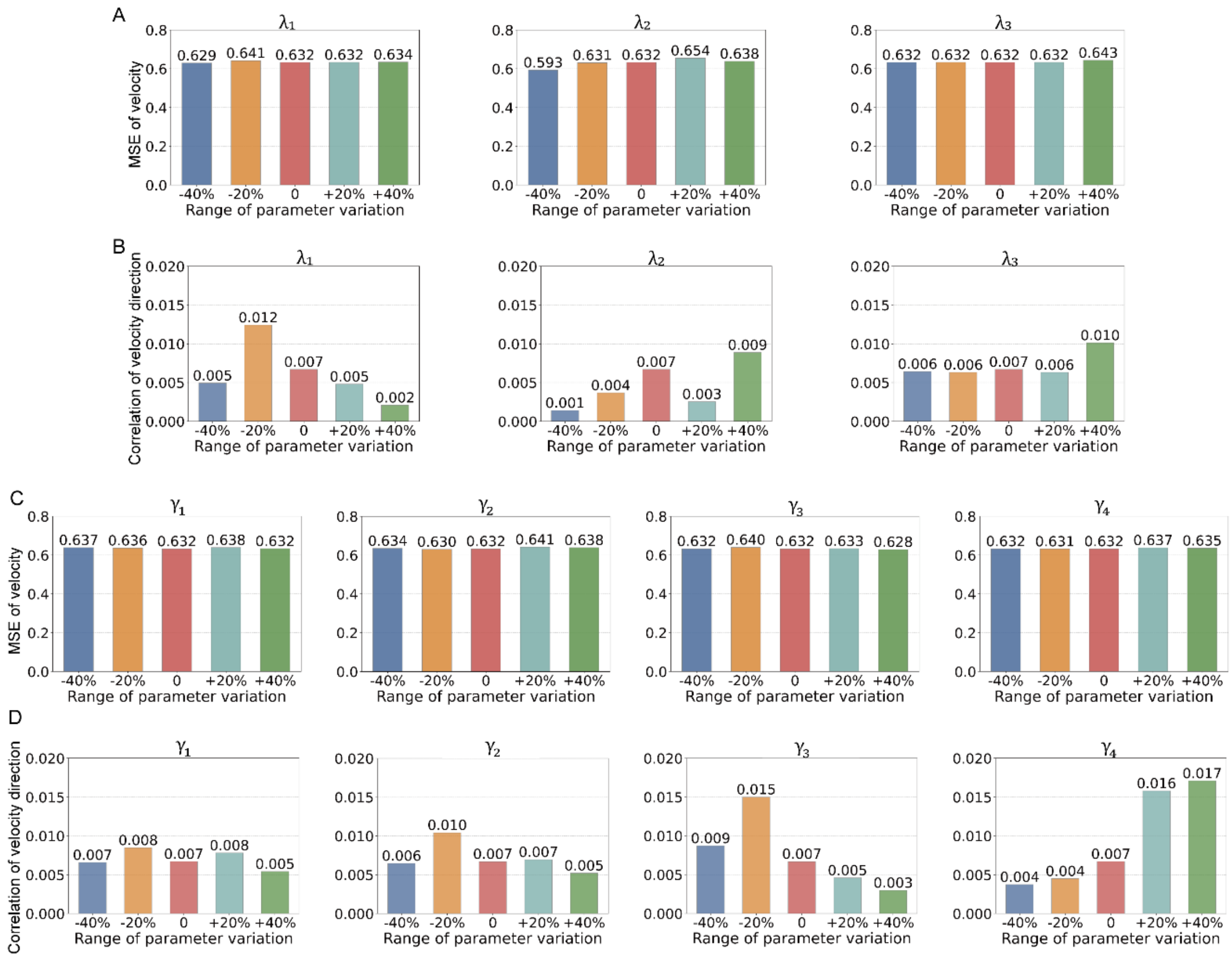

Appendix Figure S13. Sensitivity analysis of hyperparameters for the TC-PINN and MP-PINN frameworks. (A) MSE of predicted velocity versus ground truth when varying hyperparameters $\lambda_1, \lambda_2, \lambda_3$ in TC-PINN. (B) Direction correlation between predicted velocity and ground truth when varying hyperparameters $\lambda_1, \lambda_2, \lambda_3$ in TC-PINN. (C) MSE of predicted velocity versus ground truth when varying hyperparameters $\gamma_1, \gamma_2, \gamma_3, \gamma_4$ in MP-PINN. (D) Direction correlation between predicted velocity and ground truth when varying hyperparameters $\gamma_1, \gamma_2, \gamma_3, \gamma_4$ in MP-PINN.

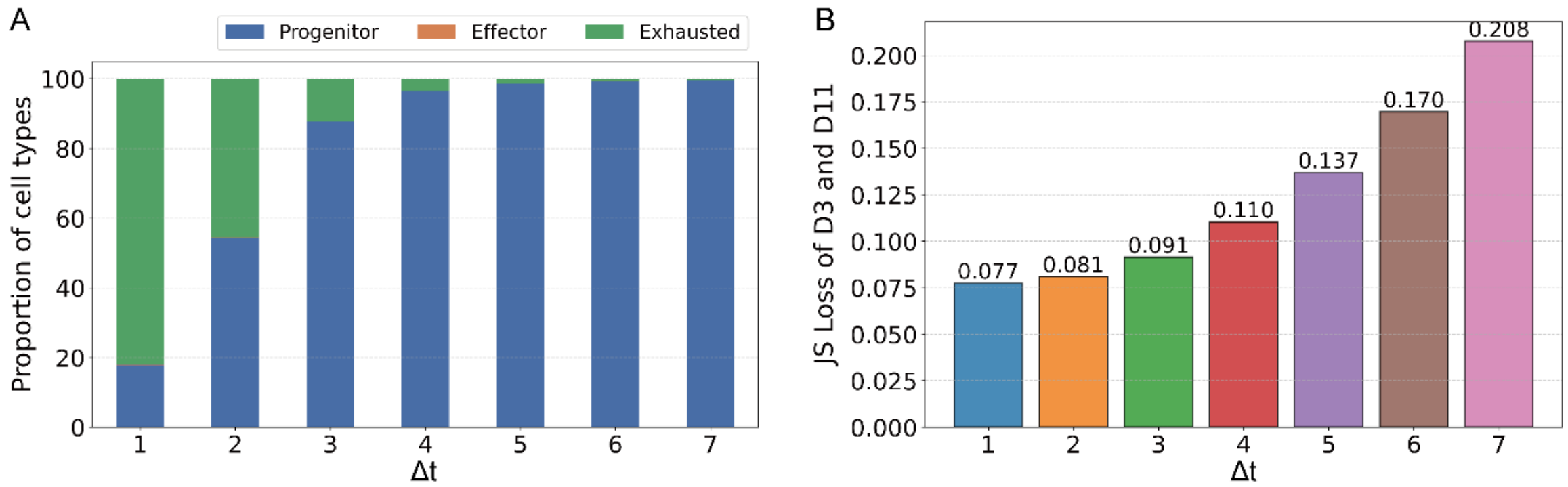

Appendix Figure S14. Sensitivity analysis of hyperparameter $\boldsymbol{\Delta t}$ in perturbation simulations. (A) For CD8+ T cells, predicted cell type proportions at day 8 after perturbation under different values of hyperparameter $\boldsymbol{\Delta t}$. (B) For PC9 tumor cells, distance between perturbed cells at day 3 and late-stage tumors (D11) under varying Criz doses, evaluated under different values of hyperparameter $\boldsymbol{\Delta t}$.

Appendix Tables

Appendix Table S1. Network centrality scores for genes in the EGFR downstream GRN.

	Degree	Betweenness	Closeness	Eigenvector
TPM1	0.9	0.866667	0.909091	0.599568
IGFBP3	0.4	0.2	0.625	0.409126
EGFR	0.1	0	0.4	0.112952
CCDC80	0.3	0	0.588235	0.384689
SERPINE1	0.3	0	0.588235	0.384689
MT-RNR2	0.1	0	0.5	0.165531
CALD1	0.1	0	0.5	0.165531
CD24	0.1	0	0.5	0.165531
PDLIM1	0.1	0	0.5	0.165531
KRT17	0.1	0	0.5	0.165531
MTRNR2L1	0.1	0	0.5	0.165531

Appendix Table S2. Network centrality scores for genes in the MET downstream GRN.

	Degree	Betweenness	Closeness	Eigenvector
EMP1	0.8	0.555556	0.833333	0.532819
HMGA1	0.6	0.311111	0.714286	0.448932
S100A4	0.5	0.133333	0.666667	0.423644
MET	0.1	0	0.434783	0.113009
ENO1	0.2	0	0.526316	0.240769
ALDOA	0.2	0	0.526316	0.240769
ITGA6	0.2	0	0.555556	0.247136
HIST1H4C	0.1	0	0.47619	0.134126
S100A6	0.2	0	0.555556	0.247136
AGR2	0.2	0	0.5	0.219652
TUBA1B	0.1	0	0.47619	0.134126

Appendix Table S3. Computational cost of the GRNvelo model on synthetic datasets.

Dataset	Cell number	Gene number	Running time	Memory usage
3-gene	2,778	3	1.49 min	0.58 GB
10-gene	2,851	10	2.46 min	0.94 GB
10-gene_2	100,000	10	38.85 min	8.36 GB

Note: All experiments were performed on a computer with an AMD Ryzen 9 7945HX (2.50 GHz), 16 GB RAM, running Windows 11. Python 3.8 was used.

Appendix Table S4. Computational cost of the GRNvelo model on real datasets.

Dataset	Cell number	Gene number	Running time	Memory usage
PC9 tumor cell	2,008	8437	2.05 min	1.24 GB
Lineage	15,007	2	15.75 min	1.10 GB
CD8+ T cell	50,577	16710	13.46 min	6.35 GB
Pancreas	51,274	2500	5.55 min	3.16 GB
C. elegans	8,9701	20222	45.54 min	6.04 GB

Note: All experiments were performed on a computer with an AMD Ryzen 9 7945HX (2.50 GHz), 16 GB RAM, running Windows 11. Python 3.8 was used.

Appendix Table S5. Hyperparameters in neural network of TC-PINN.

	**Simulation datasets**			**Real datasets**				
**Hyperparameters**	**3-gene**	**10-gene**	**10-gene_2**	**Pancreas**	**Lineage**	**PC9 tumor cell**	**CD8+ T cell**	**C. elegans**
Number of cluster-level lineages	2	2	2	4	2	1	1	2
Number of hidden layers	2/2	2/2	2/2	2/2/2/2	2/2	2	2	2/2
Number of hidden nodes	10/50	30/30	30/30	40/40/40/40	30/30	40	40	40
Learning rate	0.01	0.01	0.01	0.005	0.001	0.01	0.01	0.01
Number of iterations for fixing latent time	400	400	400	500	500	500	500	500
Number of iterations for updating latent time	400	400	400	500	2500	0	300	300
λ_1	1	1	1	1	0.3	1	1	1
λ_2	0.001	0.001	0.001	0.1	0.0001	0.1	0.1	0.1
λ_3	1	1	1	1	1	1	1	1

Notes: for training $NN_1(\theta)$, the loss function is defined as follows:
$loss = \lambda_1 * loss_{data} + \lambda_2 * loss_f + \lambda_3 * loss_{lt}$.

Appendix Table S6. Hyperparameters in neural network of MP-PINN.

	Simulation datasets			Real datasets				
**Hyperparameters**	**3-gene**	**10-gene**	**10-gene-2**	**Pancreas**	**Lineage**	**PC9 tumor cell**	**CD8+ T cell**	**C. elegans**
Number of hidden layers of the V network	2	2	2	2	2	2	2	2
Number of hidden nodes of the V network	20	10	10	30	30	30	30	30
Number of hidden layers of the ρ network	2	2	2	2	2	2	2	2
Number of hidden nodes of the ρ network	20	10	10	20	20	20	20	20
Number of hidden layers of the g network	2	2	2	2	2	2	2	2
Number of hidden nodes of the g network	20	10	10	20	20	20	20	20
Learning rate	0.001	0.001	0.001	0.001	0.001	0.01	0.01	0.01
Number of iterations	800	800	800	1000	2000	1000	1000	1000
γ_1	1	1	1	1	1	1	1	1
γ_2	1	1	1	1	0.5	1	1	1
γ_3	1	1	1	1	0.1	1	1	1
γ_4	1	1	1	1	1	1	1	1

Notes: for training $NN_2(\theta)$, the loss function is defined as follows:
$loss = \gamma_1 * loss_\rho + \gamma_2 * loss_{v_{size}} + \gamma_3 * loss_{v_{direction}} + \gamma_4 * loss_f.$
