# Supplementary material for: Multiscale learning of gene network-driven phenotypic dynamics of single cells
Source: Mol Syst Biol. 2026 Jun 5;22(8):1333–59. doi: 10.1038/s44320-026-00220-x (PMC13434742; doi:10.1038/s44320-026-00220-x)
Supplement: Supplementary file 2 [file 44320_2026_220_MOESM2_ESM.pdf]

Multiscale learning of gene network-driven phenotypic dynamics of single cells

Dongyan Zhang, Jinan Li, Qing Nie, and Xiaoqiang Sun

Corresponding author(s): Xiaoqiang Sun (sunxq6@mail.sysu.edu.cn)

Review Timeline:

Submission Date:	12th Jan 26
Editorial Decision:	18th Feb 26
Revision Received:	8th Apr 26
Editorial Decision:	4th May 26
Revision Received:	6th May 26
Accepted:	19th May 26

Editor: Poonam Bheda

Transaction Report:

(Note: With the exception of the correction of typographical or spelling errors that could be a source of ambiguity, letters and reports are not edited. Depending on transfer agreements, referee reports obtained elsewhere may or may not be included in this compilation. Referee reports are anonymous unless the Referee chooses to sign their reports.)

18th Feb 2026

Manuscript Number: MSB-2026-13577
Title: Multiscale learning of dynamic gene network-driven phenotypic evolution of single cells

Dear Prof Sun,

Thank you for the submission of your manuscript to Molecular Systems Biology. We have now received feedback from the three reviewers who agreed to evaluate your manuscript. As you will see from the reports below, the referees acknowledge the interest of the study and are overall supporting publication of your work pending appropriate revisions.

I think that the recommendations of the reviewers are rather clear and I therefore do not see the need to repeat the comments listed below. All issues raised would need to be satisfactorily addressed. On an editorial note, we would like to emphasize in line with Reviewer 3's comment that the code should be made available to referees for review. Please let me know in case you would like to discuss in further detail any of the any of the reviewer comments or your proposed revisions, I would be happy to schedule a call.

When submitting your revised manuscript, please carefully review the instructions that follow below. We perform an initial quality control of all revised manuscripts before re-review; failure to include requested items will delay the evaluation of your revision.

We require:

1) A .docx formatted version of the manuscript text (including legends for main figures, EV figures and tables). Please make sure that the changes are highlighted to be clearly visible. Alternatively you may choose to submit your manuscript as a LaTeX file.

2) Individual production quality figure files as .eps, .tif, .jpg (one file per figure). For guidance, download the 'Figure Guide PDF' (https://media.springernature.com/original/springer-cms/rest/v1/content/27825798/data/v1).

3) At EMBO Press we ask authors to provide source data for the main figures. Our source data coordinator will contact you to discuss which figure panels we would need source data for and will also provide you with helpful tips on how to upload and organize the files.

4) A .docx formatted letter INCLUDING the reviewers' reports and your detailed point-by-point responses to their comments. As part of the EMBO Press transparent editorial process, the point-by-point response is part of the Peer Review File (PRF), which will be published alongside your paper.

5) A complete author checklist, which you can download from our author guidelines (https://link.springer.com/journal/44320/submission-guidelines#cms). Please insert information in the checklist that is also reflected in the manuscript. The completed author checklist will also be part of the PRF.

6) Please note that all corresponding authors are required to supply an ORCID ID for their name upon submission of a revised manuscript.

7) It is mandatory to include a 'Data Availability' section after the Materials and Methods. Before submitting your revision, primary datasets produced in this study need to be deposited in an appropriate public database, and the accession numbers and database listed under 'Data Availability'. Please remember to provide a reviewer password if the datasets are not yet public (see https://link.springer.com/partners/embo-press/editorial-policies#Data%20availability%20statement).

In case you have no data that requires deposition in a public database, please state so in this section as follows: "This study includes no data deposited in external repositories". Note that the Data Availability Section is restricted to new primary data that are part of this study.

8) All Materials and Methods need to be described in the main text using our 'Structured Methods' format, which is required for all research articles. According to this format, the Methods section includes a Reagents and Tools Table (listing key reagents, experimental models, software and relevant equipment and including their sources and relevant identifiers) followed by a Methods and Protocols section describing the methods using a step-by-step protocol format. The aim is to facilitate adoption of the methodologies across labs. Please upload the Reagents and Tools table as a separate document when submitting your revised manuscript. More information on how to adhere to this format as well as a downloadable template (.docx) for the Reagents and Tools Table can be found in our author guidelines: https://link.springer.com/journal/44320/submission-guidelines#cms

9) For data quantification: please specify the name of the statistical test used to generate error bars and p-values, the number (n) of independent experiments (specify technical or biological replicates) underlying each data point and the test used to calculate p-values in each figure legend. The figure legends should contain a basic description of n, p-values and the test applied. Graphs must include a description of the bars and the error bars (s.d., s.e.m.). Please provide exact p-values (in either the figure or figure legend).

10) Our journal encourages inclusion of *data citations in the reference list* to directly cite datasets that were re-used and obtained from public databases. Data citations in the article text are distinct from normal bibliographical citations and should directly link to the database records from which the data can be accessed. In the main text, data citations are formatted as follows: "Data ref: Smith et al, 2001" or "Data ref: NCBI Sequence Read Archive PRJNA342805, 2017". In the Reference list, data citations must be labeled with "[DATASET]". A data reference must provide the database name, accession number/identifiers and a resolvable link to the landing page from which the data can be accessed at the end of the reference. Further instructions are available at .

11) We replaced Supplementary Information with Expanded View (EV) Figures and Tables that are collapsible/expandable online. EV Figures should be cited as 'Figure EV1, Figure EV2" etc... in the text and their respective legends should be included in the main text after the legends of regular figures.
- For the figures that you do NOT wish to display as Expanded View figures, they should be bundled together with their legends in a single PDF file called *Appendix*, which should start with a short Table of Content. Appendix figures should be referred to in the main text as: "Appendix Figure S1, Appendix Figure S2" etc.
- Additional Tables/Datasets should be labeled and referred to as Table EV1, Dataset EV1, etc. Legends should be provided in a separate tab in case of .xls files. Alternatively, the legend can be supplied as a separate text file (README) and zipped together with the Table/Dataset file.
See detailed instructions here:
https://link.springer.com/journal/44320/submission-guidelines#cms

12) Author contributions: CRediT has replaced the traditional author contributions section because it offers a systematic machine-readable author contributions format that allows for more effective research assessment. Please remove the Authors Contributions from the manuscript and use the free text boxes beneath each contributing author's name in our system to add specific details on the author's contribution. More information is available in our guide to authors.

13) Disclosure statement and competing interests: We updated our journal's competing interests policy in January 2022 and request authors to consider both actual and perceived competing interests. Please review the policy https://link.springer.com/partners/embo-press/editorial-policies#Competing%20interest%20disclosures and update your competing interests if necessary.

14) Every published paper now includes a 'Synopsis' to further enhance discoverability. Synopses are displayed on the journal webpage and are freely accessible to all readers. They include a short stand first (maximum of 300 characters, including space) as well as 2-5 one-sentences bullet points that summarizes the paper. Please write the bullet points to summarize the key NEW findings. They should be designed to be complementary to the abstract - i.e. not repeat the same text. We encourage inclusion of key acronyms and quantitative information (maximum of 30 words / bullet point). Please use the passive voice. Please attach these in a separate file or send them by email, we will incorporate them accordingly.

Please also suggest a striking image or visual abstract to illustrate your article as a PNG file 550 px wide x 300-600 px high.

Please note that these would be the final versions and changes during proofing are usually not allowed.

15) As part of the EMBO Publications transparent editorial process initiative (see our policy here: https://link.springer.com/partners/embo-press/editorial-policies#Peer%20review), Molecular Systems Biology will publish online a Peer Review File (PRF) to accompany accepted manuscripts.
In the event of acceptance, this file will be published in conjunction with your paper and will include the anonymous referee reports, your point-by-point response and all pertinent correspondence relating to the manuscript. Let us know whether you agree with the publication of the PRF and as here, if you want to remove or not any figures from it prior to publication.
Please note that the Author checklist will be published at the end of the PRF.

Molecular Systems Biology has a "scooping protection" policy, whereby similar findings that are published by others during review or revision are not a criterion for rejection. Should you decide to submit a revised version, I do ask that you get in touch after three months if you have not completed it, to update us on the status.

Yours sincerely,

Poonam Bheda, PhD
Scientific Editor

Reviewer #1:

This study introduces GRNvelo, a novel computational framework designed to reconstruct multiscale cell fate dynamics by integrating gene regulatory networks (GRNs) with cell phenotypic evolution using temporal single-cell RNA-seq data. The framework employs a two-phase cooperative optimization algorithm based on physics-informed neural networks (PINNs): TC-PINN calculates GRN velocity and latent time by enforcing temporal consistency, while MP-PINN refines these velocities within the context of population dynamics, including cell growth and density. Validated across synthetic and real-world datasets-such as pancreatic β-cell differentiation, lineage tracing, and drug-resistant tumor cells-GRNvelo demonstrates superior performance in inferring trajectory, velocity, and growth compared to existing methods. Crucially, the model allows for predictive simulations of complex genetic perturbations and drug combinations, offering new insights into modulating cell fate outcomes in development and disease.I have the following questions regarding the manuscript before recommending for publication:

1. How does the integration of spatial transcriptomics data with GRNvelo potentially enhance the accuracy of inferring cell-cell interactions during fate transitions?

2. What are the computational limitations of GRNvelo when scaling to extremely large datasets with hundreds of thousands of cells?

3. Can the GRNvelo framework be adapted to model cell fate dynamics in non-temporal or static single-cell datasets without explicit time-series information?

Reviewer #2:

In this manuscript, the authors present GRNvelo, a sophisticated multiscale framework that integrates gene regulatory networks with population dynamics to infer cell fate trajectories from time-series scRNA-seq data. The method is technically innovative, combining physics-informed neural networks with mechanistic ODE/PDE modeling, and is rigorously validated on both synthetic and real datasets. The ability to predict perturbation outcomes is particularly impressive.

Overall, this is an interesting study that proposes a valuable way for trajectory inference and cell fate decision mechanisms study by integrating single cell data and mechanistic modelling. Here I have some comments for the authors' consideration, and I believe that addressing these comments will help to improve the manuscript.

1, In this work, the method appears to model branching trajectories via separate, lineage-specific parameter sets for the GRN ODEs (according to the cluster-level lineage identification and independent TC-PINN training). This is conceptually distinct from classical multistability, where a single fixed-parameter system possesses multiple attractors, and fate transitions occur via stochastic hopping between them on an energy landscape (Kang et al., Advanced Science, 8: 2003133 (2021), Zhang et al., PNAS, 111: 10185 (2014)).
It seems that using the current framework, which fits distinct parameters to each branch, it's hard to analyze stochastic transitions between coexisting fates under constant conditions-a central theme in cell fate decision theories. It's suggested that the authors should add above references and related clarifications or discussions on this point.

2, A major methodology novelty is introduction of two PINN network. Some details need further clarification. For example, in Eq. (11), aij and bij are gene regulation parameters. Are they estimated along with the neural network? If so, did the authors use these estimated aij and bij in the following downstream analysis?
3, Supplementary Fig. 1A and B, from the simulation trajectories, the noise level seems very small. Did the authors try larger noise level?
4, Supplementary Fig. 2C, D and E, from Supplementary Fig. 2E, the system dynamics display bifurcations, however, this was not shown in trajectories in Supplementary Fig. 2C and D. Can the authors make clarifications on this?

5, The choice and impact of key hyperparameters (e.g., loss function weights λ_i, γ_i; network propagation time Δt; autoencoder architecture) are not sufficiently discussed. A brief description of the tuning strategy (e.g., grid search, validation-based selection) should be added to the Methods. Furthermore, a sensitivity analysis (e.g., in the Supplementary Information) demonstrating the robustness of results to variations in these parameters would strengthen the methodological rigor.

6, The description of how the high-dimensional GRN is recovered from the low-dimensional embedding (page 28) is currently difficult to follow. The presented formula (line 1131) seems problematic. Specifically, the second equality appears to be missing

a differential in the numerator (dV_h vs. dx_h/dt). Please clarify the derivation logic and correct the formula if necessary. A more intuitive explanation of this mapping process is recommended.

Minor points:
1, Fig. 4A, the color for D2 an D6 are similar, please use better color scheme.
2，The terms "latent time" and "pseudotime" are used throughout the manuscript. Please ensure their definitions are clearly stated and applied consistently. Specifically, please clarify how the "latent time" inferred by GRNvelo differs conceptually from the "pseudotime" estimated by conventional methods.
3, It might be useful to add a table on the computational cost of the proposed method.

Reviewer #3:

The manuscript presents GRNvelo, an analysis tool for establishing biologically interpretable and mathematically rigorous multiscale models, which couple GRN-driven single-cell velocity with cell growth-mediated population dynamics. This is an important and formidable problem that the GRNvelo tool addresses thoroughly, with ample demonstrations with simulated and real biological data, with improved performance over existing alternatives and the ability to predict altered cell fates in response to diverse genetic perturbations. Demonstrations exist for data simulated in a 3-gene and a 10-gene network, and for real data collected from beta-cell differentiation, murine hematopoiesis, CD8+ T cell responses to infection, and lung cancer cells' drug responses.

Overall, the manuscript offers a valuable contribution and should be published, after the following comments have been addressed.

(1) A bit of a historical background with some citations might be useful on the experimental demonstration of the problem addressed by the tool: coupled GRN-driven single-cell dynamics and cell growth-mediated population dynamics. Probably the simplest examples emerged in synthetic biology, see PMID:22511863 and PMID: 30341217
(2) GRNvelo seems to be operating on the assumption that stochasticity can be modeled by an additive noise term. However, many studies of stochastic chemical kinetics indicate that noise can arise inherently from the stochastic nature of reactions. This means that SDEs might not be appropriate. Could Gillespie simulations of the Chemical Master Equation be tested to see how GRNvelo works with that simulated data?
(3) Adding the simplest possible simulations to generate data for GRNvelo based on very simple synthetic biological systems, like a simple positive autoregulation (including with Gillespie simulations) could be interesting - in addition, before the 3-node and 10-node networks.
(4) What would experimental biologists do differently after GRNvelo is published? What new experiments would GRNvelo suggest that would not have been conceived without GRNvelo? This question should be answered specifically for the data that was analyzed as examples, and in general, for other data that will be analyzed.
(5) The phrase "phenotypic evolution dynamics" or "cell state evolution" might create some confusion. Here it means time-evolution in the mathematical sense. However, in the biology community, this means the phenotypic manifestation of some biological evolutionary process. It would be better to use "phenotypic time course" or "phenotypic dynamics" or something else to avoid connotations of biological (such as Darwinian) evolution.
(6) It is actually interesting to ask, can GRNvelo be developed towards predicting longer-term evolutionary processes in the biological sense? If cellular growth rates differ, that means there is evolutionary pressure.
(7) Some of the fonts are too small in multiple figures (axis and bar labels, colorbar marks, etc.)
(8) The code is not accessible. https://github.com/SunXQlab/%20GRNvelo is giving an error.
(9) The code should be friendly and accessible for interested biologists to be able to try and even modify for processing other data. Consider developing a GUI - or at least propose so for the future.

Response to Reviewer #1

This study introduces GRNvelo, a novel computational framework designed to reconstruct multiscale cell fate dynamics by integrating gene regulatory networks (GRNs) with cell phenotypic evolution using temporal single-cell RNA-seq data. The framework employs a two-phase cooperative optimization algorithm based on physics-informed neural networks (PINNs): TC-PINN calculates GRN velocity and latent time by enforcing temporal consistency, while MP-PINN refines these velocities within the context of population dynamics, including cell growth and density. Validated across synthetic and real-world datasets-such as pancreatic β-cell differentiation, lineage tracing, and drug-resistant tumor cells-GRNvelo demonstrates superior performance in inferring trajectory, velocity, and growth compared to existing methods. Crucially, the model allows for predictive simulations of complex genetic perturbations and drug combinations, offering new insights into modulating cell fate outcomes in development and disease. I have the following questions regarding the manuscript before recommending for publication:

Thank you for your positive feedback and insightful comments. Now we have carefully revised the manuscript according to your constructive suggestions. We believe that we have addressed all questions you raised and that the manuscript now has been significantly improved. Below we respond to your comments point-by-point.

1. How does the integration of spatial transcriptomics data with GRNvelo potentially enhance the accuracy of inferring cell-cell interactions during fate transitions?

R: Thank you for your insightful comment. We agree that integrating spatial transcriptomics data holds great promise for inferring cell-cell interactions (CCIs) during fate transitions. While the current GRNvelo focuses on intracellular dynamics from temporal scRNA-seq data, its multi-scale PINN-based framework is naturally extendable to spatiotemporal data. In the revised manuscript (Discussion section, lines 717-727), we have explicitly added the following statement:

"While GRNvelo currently focuses on intracellular dynamics from temporal scRNA-seq data, its multi-scale PINN-based framework can be extended to spatiotemporal data for inferring cell-cell interactions (CCIs). Existing methods like DeepTalk (Yang *et al*, 2024) and stMLnet (Yan *et al*, 2025) incorporate spatial information into cell-cell communication (CCC) inference but fail to connect CCC with phenotypic dynamics. CCCvelo (Yan *et al*, 2026) couples CCC with single-cell velocity but does not consider the constraint of cell population dynamics (e.g., proliferation, density feedback). In the future, an extended GRNvelo framework could incorporate the modulation of ligand-receptor-mediated CCC on gene expression velocity in TC-PINN, while capturing more general (e.g., non-local) CCIs through the MP-PINN module's explicit modeling of density-dependent dynamics. Such an extension would improve CCI inference accuracy and enable predictive simulations of CCI perturbations."

2. What are the computational limitations of GRNvelo when scaling to extremely large datasets with hundreds of thousands of cells?

R: Thank you for your good comment. According to your comment, we first generated a synthetic dataset with 100,000 cells (10-gene topology) to evaluate the scalability of GRNvelo. Furthermore, we tested GRNvelo on a real C. elegans dataset containing approximately 90,000 cells to further assess computational cost on a genuine large-scale dataset. We would like to note that we have searched and checked a lot of databases and publications but did not find real time-series scRNA-seq dataset that exactly exceeds hundreds of thousands of cells, and that the C. elegans dataset is the largest time-series scRNA-seq dataset we can find until now. For both the synthetic large dataset and the real large dataset, we recorded runtime and peak memory usage. The results for the synthetic large dataset are summarized in Appendix Table S3, and those for the real large dataset are shown in Appendix Table S4 (both included below). The results demonstrated that while, as expected, the computational costs were substantially higher for the large datasets, they remain within an acceptable range for application. We have added a discussion of this scalability consideration in the Discussion section (lines 731-738).

"In addition, we also evaluated the scalability of GRNvelo on multiple synthetic and real datasets of varying sizes, including a 100,000-cell synthetic dataset and the ~90,000-cell Caenorhabditis elegans (C. elegans) dataset (Packer *et al*, 2019; Data ref: Ullrich, 2022). The runtime and memory usage for all tested datasets are summarized in Appendix Tables S3 and S4. Nevertheless, for most existing and available time-series scRNA-seq datasets (which typically contain fewer than one hundred thousand cells), GRNvelo remains computationally feasible on standard workstations. In future studies, improving the computational efficiency of GRNvelo on large-scale datasets will increase its practical value."

Appendix Table S3. Computational cost of the GRNvelo model on synthetic datasets.

**Dataset**	**Cell number**	**Gene number**	**Running time**	**Memory usage**
3-gene	2,778	3	1.49 min	0.58 GB
10-gene	2,851	10	2.46 min	0.94 GB
10-gene_2	100,000	10	38.85 min	8.36 GB

Appendix Table S4. Computational cost of the GRNvelo model on real datasets.

**Dataset**	**Cell number**	**Gene number**	**Running time**	**Memory usage**
PC9 tumor cell	2,008	8437	2.05 min	1.24 GB
Lineage	15,007	2	15.75 min	1.10 GB
CD8+ T cell	50,577	16710	13.46 min	6.35 GB
Pancreas	51,274	2500	5.55 min	3.16 GB
C. elegans	89701	20222	45.54 min	6.04 GB

3. Can the GRNvelo framework be adapted to model cell fate dynamics in non-temporal or static single-cell datasets without explicit time-series information?

R: Thank you for your constructive comment. Adapting GRNvelo to static data would require incorporating additional information to compensate for the absence of temporal labels. We have elaborated on these possibilities (e.g., using clock genes to constrain

latent time for TC-PINN, and leveraging OSDR-like strategies for MP-PINN) in the Discussion section of the revised manuscript (lines 695-707).

"Extending GRNvelo to static datasets would require incorporating additional information to compensate for the absence of temporal labels. For the TC-PINN module, without time-stamped observations, one could incorporate prior temporal information using genes that are known to correlate with time (e.g., cell-cycle or differentiation-associated genes) to constrain the latent time. This approach has precedent in methods that use "clock" genes to order cells, e.g., Revelio (Schwabe *et al*, 2020) and DeepCycle (Riba *et al*, 2022). Alternatively, another line of work (Zhu & Wang, 2024; Zhu *et al*, 2024) reconstructs continuous vector fields directly from RNA velocity without requiring predefined clock genes, which may also inspire this direction. For the MP-PINN module, which infers cell growth from density changes over time, such dynamics could be estimated in static data by leveraging static spatial snapshots as demonstrated in OSDR (Somer *et al*, 2026): OSDR uses cell division markers (e.g., Ki67) and the composition of local cellular neighborhoods to infer proliferation rates, effectively reconstructing population dynamics without temporal sequences."

Response to Reviewer #2

In this manuscript, the authors present GRNvelo, a sophisticated multiscale framework that integrates gene regulatory networks with population dynamics to infer cell fate trajectories from time-series scRNA-seq data. The method is technically innovative, combining physics-informed neural networks with mechanistic ODE/PDE modeling, and is rigorously validated on both synthetic and real datasets. The ability to predict perturbation outcomes is particularly impressive.

Overall, this is an interesting study that proposes a valuable way for trajectory inference and cell fate decision mechanisms study by integrating single cell data and mechanistic modelling. Here I have some comments for the authors' consideration, and I believe that addressing these comments will help to improve the manuscript.

Thank you for your positive feedback and insightful comments. Now we have carefully revised the manuscript according to your constructive suggestions. We believe that we have addressed all questions you raised and that the manuscript now has been significantly improved. Below we respond to your comments point-by-point.

1, In this work, the method appears to model branching trajectories via separate, lineage-specific parameter sets for the GRN ODEs (according to the cluster-level lineage identification and independent TC-PINN training). This is conceptually distinct from classical multistability, where a single fixed-parameter system possesses multiple attractors, and fate transitions occur via stochastic hopping between them on an energy landscape (Kang et al., Advanced Science, 8: 2003133 (2021), Zhang et al., PNAS, 111: 10185 (2014)).
It seems that using the current framework, which fits distinct parameters to each branch, it's hard to analyze stochastic transitions between coexisting fates under constant conditions-a central theme in cell fate decision theories. It's suggested that the authors should add above references and related clarifications or discussions on this point.

R: Thank you for your insightful comment. We have added a new paragraph in the Discussion section to explicitly distinguish GRNvelo's modeling paradigm from classical multistability frameworks, clarifying that our focus is on fate prediction under non-stationary conditions rather than analysis of stochastic transitions. The added text (see Discussion, lines 673-683) cites the recommended references (Zhang et al., 2014; Kang et al., 2021) and aligns with the reviewer's insightful observation.

"GRNvelo models branching trajectories by fitting lineage-specific GRN parameters, which differs from classical multistability frameworks where a single fixed-parameter system gives rise to multiple attractors and stochastic transitions between them (Kang & Li, 2021; Zhang *et al*, 2014). This design reflects GRNvelo's focus on predicting cell fate outcomes from time-series snapshot data, particularly in non-stationary developmental processes where the regulatory landscape evolves over time due to external signals or epigenetic remodeling. In such contexts, branch-specific parameterization offers a flexible and data-driven approach for reconstructing trajectory dynamics and inferring

terminal fates. We note that fate prediction and mechanistic understanding of stochastic decision-making are complementary goals; integrating data-driven velocity or trajectory inference with energy landscape theories represents a promising direction for future investigation."

2, A major methodology novelty is introduction of two PINN network. Some details need further clarification. For example, in Eq. (11), aij and bij are gene regulation parameters. Are they estimated along with the neural network? If so, did the authors use these estimated aij and bij in the following downstream analysis?

R: Thank you for your positive evaluation and insightful comment. In the revised manuscript, we have added a dedicated paragraph in the Methods section (lines 883-894) to explain their biological meanings of a_{ij} and b_{ij} and to clarify that these parameters are jointly estimated with the neural network during training. These parameters are used within the TC-PINN to enforce transcriptional regulatory constraints; they are not directly employed in downstream perturbation analyses (which instead rely on the GRN derived from the MP-PINN module). Nevertheless, these two parameters enhance the model interpretability and their different combinations implicitly shape the velocity field, thereby influencing the downstream analysis. The added text is attached below for reference.

"In Eq. (11), a_{ij} and b_{ij} denote the maximum transcriptional activation and repression rates, respectively, from regulator gene j to target gene i. Specifically, a_{ij} scales the Hill activation term, while b_{ij} scales the Hill repression term. These parameters collectively define the strength and type (activation or repression) of each regulatory interaction. Biologically, a regulatory interaction is either activation or repression, but not both. Consequently, during training we enforce that for each gene pair (i, j), at most one of a_{ij} and b_{ij} is non-zero (i.e., either $a_{ij} > 0$ and $b_{ij} = 0$, or $a_{ij} = 0$ and $b_{ij} > 0$, or both zero if no regulation exists). Within the TC-PINN module, a_{ij} and b_{ij} are treated as trainable variables and are jointly estimated with the neural network weights by minimizing the composite loss function (including $Loss_{data}$, $Loss_f$ and $Loss_{lt}$). The specific combination of a_{ij} and b_{ij} directly determines the predicted velocity. Different combinations yield distinct velocity fields, which govern the dynamical evolution of gene expression. Incorporating a_{ij} and b_{ij} increases the model interpretability."

3, Supplementary Fig. 1A and B, from the simulation trajectories, the noise level seems very small. Did the authors try larger noise level?

R: Thank you for your constructive suggestion. We have now generated synthetic datasets with higher noise levels (standard deviation = 0.02-0.10) using the same 3-gene network and evaluated GRNvelo's performance. The results are summarized in Appendix Figure S3 and the following text has been added to the Results section (lines 239-244):

"Additionally, to evaluate robustness under higher noise, we generated synthetic datasets from the 3−gene network with noise standard deviations ranging from 0.02 to 0.10. Across all noise levels, the velocity magnitude MSE remained below 0.005. GRNvelo maintained stable performance for noise levels ≤ 0.06, with velocity direction correlation > 0.5 and latent

time correlation > 0.6, while performance slightly declined at higher noise levels (Appendix Figure S3).

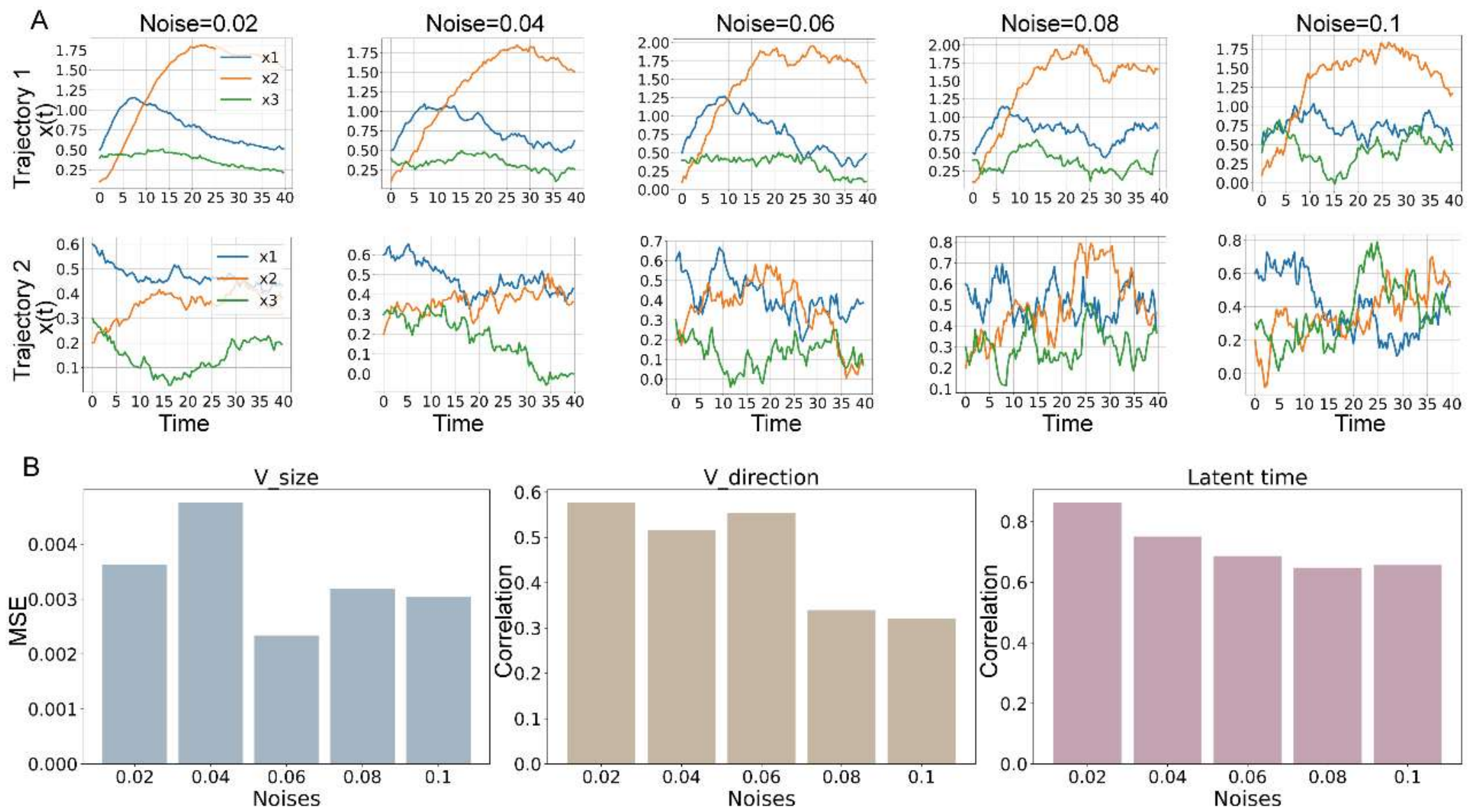

Appendix Figure S3. Performance of GRNvelo on simulated data under different noise levels. (**A**) Examples of gene dynamics for simulated data under different noise levels (standard deviation = 0.02, 0.04, 0.06, 0.08, 0.1). (B) Performance evaluation of GRNvelo on simulated data under different noise levels, including MSE of predicted velocity magnitudes, Pearson correlation of velocity directions, and Pearson correlation of latent time."

4, Supplementary Fig. 2C, D and E, from Supplementary Fig. 2E, the system dynamics display bifurcations, however, this was not shown in trajectories in Supplementary Fig. 2C and D. Can the authors make clarifications on this?

R: Thank you for your careful reading. Appendix Figure S4E presents a UMAP visualization of cells from the 10-gene simulation across different time points. The bifurcation observed in this low-dimensional embedding is driven by the differential expression of three key genes Gene 3, Gene 4 and Gene 7, which exhibit distinct regulatory dynamics between the two cell fates, as shown in Appendix Figure S4C and S4D.

To more clearly demonstrate the bifurcation, we have added a new panel to Appendix Figure S4, showing the expression dynamics of Gene 3, Gene 4, and Gene 7 along the two distinct cell fate trajectories. These three genes are the key drivers of the bifurcation observed in the UMAP embedding (Appendix Fig. S4E). Correspondingly, we have updated the figure legend to describe these additions. We believe this revision fully addresses the reviewer's concern.

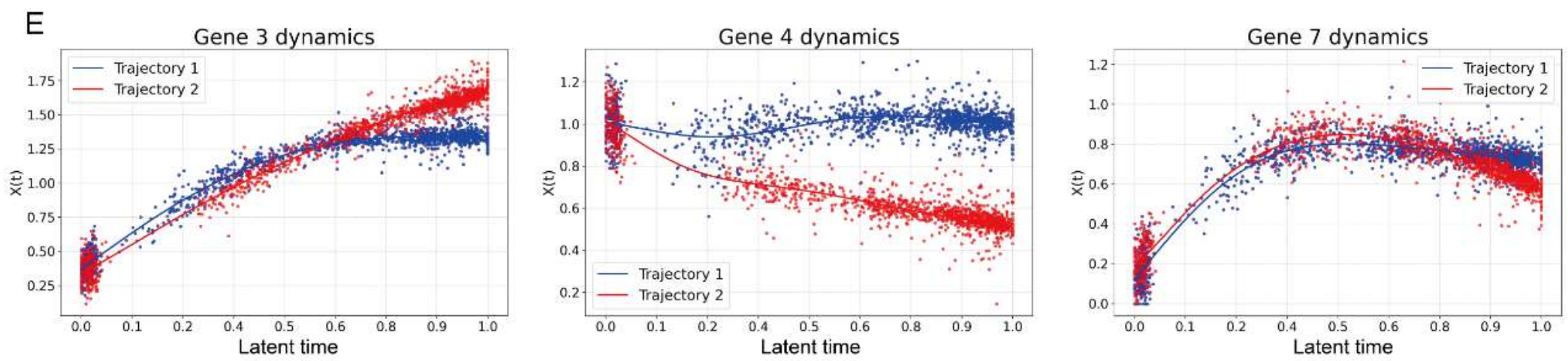

"***Appendix Figure S4. Supplementary information for 10-gene network***. *(**A**-**B**) The kinetic curves of synthetic data under the network of 10 genes.* (**C**-**D**) The kinetic curves of model fitting for two branches, where the differential expression of (**E**) three key genes (Gene 3, Gene 4 and Gene 7) defines the bifurcation. (**F**) UMAP visualization of cells from different time points, showing the branching manifold driven by the dynamics in (**C**) and (**D**). *(**G**) Pearson correlation between pseudotime and real time.*"

5, The choice and impact of key hyperparameters (e.g., loss function weights λ_i, γ_i; network propagation time Δt; autoencoder architecture) are not sufficiently discussed. A brief description of the tuning strategy (e.g., grid search, validation-based selection) should be added to the Methods. Furthermore, a sensitivity analysis (e.g., in the Supplementary Information) demonstrating the robustness of results to variations in these parameters would strengthen the methodological rigor.

R: Thank you for your constructive suggestion on clarifying tuning strategy and sensitivity analysis for these key hyperparameters. We agree that a thorough discussion of hyperparameter choices and their impact is essential for methodological rigor. To address this, we have performed sensitivity analyses on key hyperparameters and updated both the Methods section and Supplementary Information accordingly.

(1) Loss function weights ($\lambda_1 - \lambda_3$, $\gamma_1 - \gamma_4$)
We have added a description of hyperparameter tuning and sensitivity analysis for $\lambda_1 - \lambda_3$, $\gamma_1 - \gamma_4$ to the Methods section. The added text and the corresponding sensitivity figure (Appendix Figure S10) are provided below for reference.

(lines 877-882) "The loss weights $\lambda_1 - \lambda_3$ were set to 1 by default and optimized via grid search within [0,1] using a validation set. Sensitivity analysis on the synthetic 3−gene dataset (varying each weight by ±20% and ±40%) showed minimal impact on velocity directions and only slight fluctuations in magnitudes, confirming the robustness of the model with respect to these loss weights (Appendix Figure S13A)."

(lines 933-937) "The loss weights $\gamma_1 - \gamma_4$ were set to 1 by default and optimized via grid search within [0,1]. Sensitivity analysis on the synthetic 3-gene dataset (varying each weight by ±20% and ±40%) confirmed model robustness, with negligible effects on velocity directions and minor fluctuations in magnitudes (Appendix Figure S13B)."

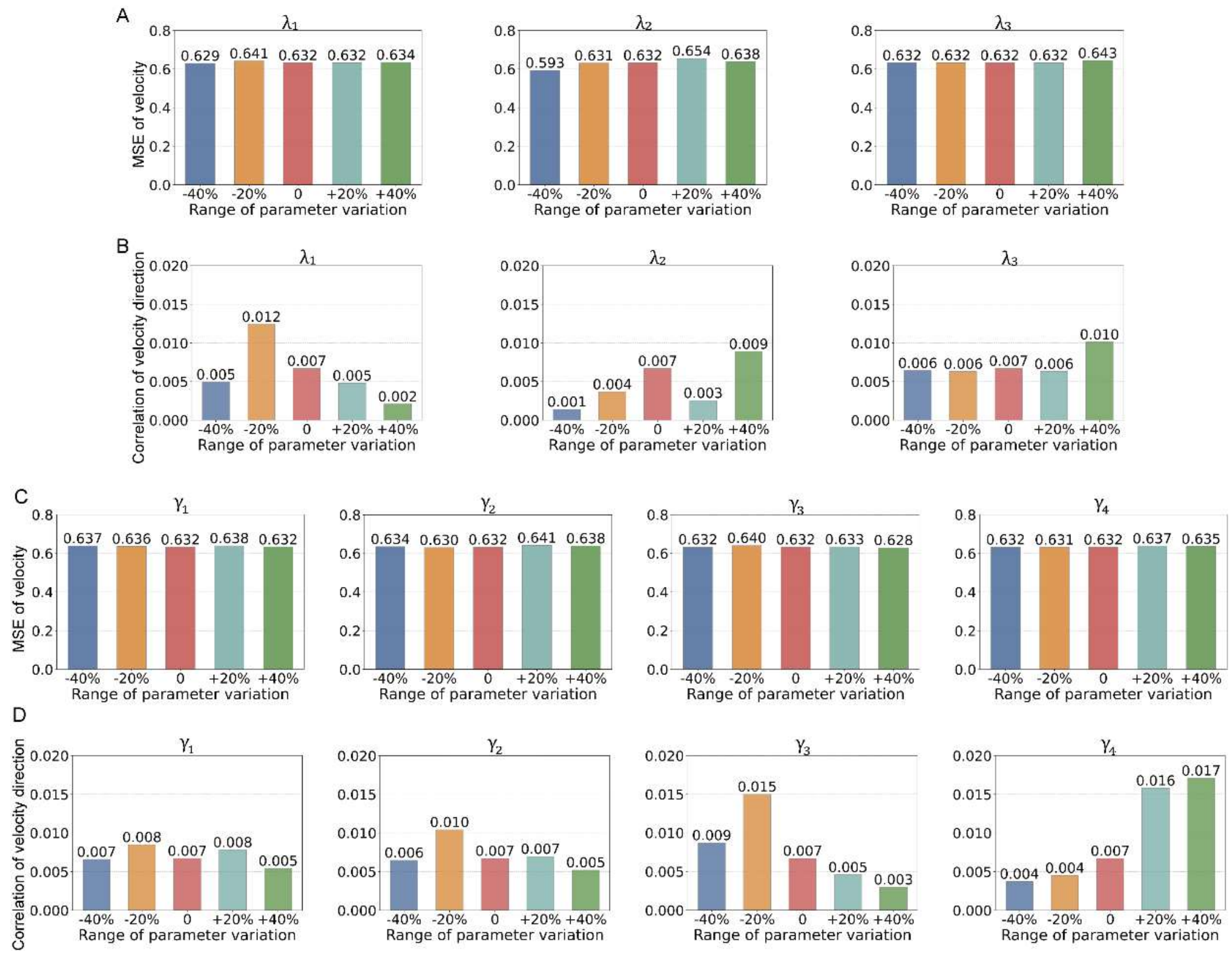

Appendix Figure S13. Sensitivity analysis of hyperparameters for the TC−PINN and MP−PINN frameworks. (**A**) MSE of predicted velocity versus ground truth when varying hyperparameters $\lambda_1, \lambda_2, \lambda_3$ in TC−PINN. (**B**) Direction correlation between predicted velocity and ground truth when varying hyperparameters $\lambda_1, \lambda_2, \lambda_3$ in TC−PINN. (**C**) MSE of predicted velocity versus ground truth when varying hyperparameters $\gamma_1, \gamma_2, \gamma_3, \gamma_4$ in MP−PINN. (**D**) Direction correlation between predicted velocity and ground truth when varying hyperparameters $\gamma_1, \gamma_2, \gamma_3, \gamma_4$ in MP−PINN.

(2) Network propagation time (Δt)

In the revised manuscript, we have added the following two paragraphs to the Methods section, placed after the description of perturbation simulations for the T cell dataset and for the PC9 dataset, respectively.

Added after single-gene KO T cell perturbation description (lines 1194-1205):

“The hyperparameter Δt represents the time required for detectable state changes of the system after perturbation. Since no ground-truth value is available, we selected $\Delta t = 3$ based on two considerations: (i) biological prior: in a study of mitochondrial unfolded protein response (UPRmt) during somatic cell reprogramming, perturbation applied on D1 led to detectable phenotypic changes (e.g., MET markers) on D3 (Ying *et al*, 2025), suggesting that a characteristic response time of approximately 3 days is plausible for gene regulatory network propagation; (ii) sensitivity analysis: over $\Delta t \in \{1, \ldots, 7\}$, the key metrics (precursor proportion at D8 for CD8+ T cells; transcriptional distance to D11 for PC9 cells) changed monotonically

and approximately linearly with Δt (Appendix Figure S14). We therefore chose the middle value $\Delta t = 3$ as a representative default. We tested and found that all qualitative conclusions remain consistent across $\Delta t \in \{2, \dots, 5\}$."

Added after multi-gene KO T cell perturbation description (lines 1223-1224):
"The hyperparameter Δt was set as described above (Appendix Figure S14A)."

Added after PC9 perturbation description (lines 1242-1243):
"The hyperparameter Δt was set as described above (Appendix Figure S14B)."

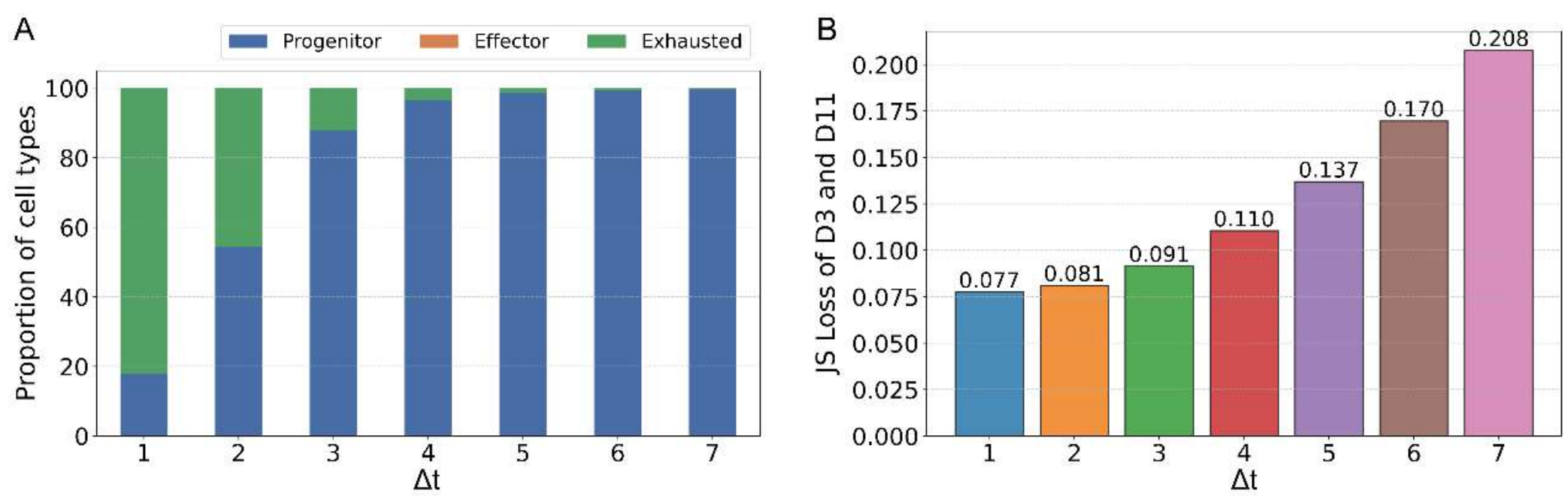

Appendix Figure S14. Sensitivity analysis of hyperparameter Δt in perturbation simulations. (**A**) For CD8+ T cells, predicted cell type proportions at day 8 after perturbation under different values of hyperparameter Δt. (**B**) or PC9 tumor cells, distance between perturbed cells at day 3 and late-stage tumors (D11) under varying Criz doses, evaluated under different values of hyperparameter Δt.

(3) Autoencoder architecture and tuning strategy

We have expanded the "Dimensionality reduction and visualization" section in the Methods to detail the AE architecture, training configuration, and the rationale for using a 10-dimensional latent space (adopting the default TIGON settings, with robustness supported by Appendix Figure S7a-e in (Sha *et al*, 2024)). These additions clarify the rationale and justification of the AE hyperparameters without requiring additional sensitivity analysis. These revisions are located in the Methods - "Dimensionality reduction and visualization" section (line 1115-1120).

"We adopted the default AE architecture following that used in TIGON (Sha *et al.*, 2024), which consists of a fully connected encoder and decoder with a single hidden layer of 300 units, ReLU activation, and a latent dimension of 10. The model is trained to minimize the mean squared error (MSE) reconstruction loss using the Adam optimizer (learning rate = 1e-3, weight decay = 1e-4, batch size = 128) for up to 500 epochs with early stopping (patience = 30)."

Furthermore, the hyperparameters of the neural networks (TC-PINN and MP-PINN) are listed in Appendix Tables S5-S6 for completeness.

We believe these additions fully address the reviewer's concern.

6, The description of how the high-dimensional GRN is recovered from the low-dimensional embedding (page 28) is currently difficult to follow. The presented formula (line 1131) seems problematic. Specifically, the second equality appears to be missing a differential in the numerator (dV_h vs. dx_h/dt). Please clarify the derivation logic and correct the formula if necessary. A more intuitive explanation of this mapping process is recommended.

R: Thank you for your carefully reading and identifying this oversight. This typo has now been corrected in the revised manuscript (line 1284). The corrected derivation is as follows:

$$GRN = \frac{\mathrm{d}V_h}{\mathrm{d}x_h} = \frac{\mathrm{d}\left(\frac{\mathrm{d}x_h}{\mathrm{d}t}\right)}{\mathrm{d}x_h} = \frac{\mathrm{d}\left(\frac{\mathrm{d}x_h}{\mathrm{d}x_l} \cdot \frac{\mathrm{d}x_l}{\mathrm{d}t}\right)}{\mathrm{d}x_h} = \frac{\mathrm{d}\left(\left(\frac{\mathrm{d}x_l}{\mathrm{d}x_h}\right)^{-1} \cdot V_l\right)}{\mathrm{d}x_h}.$$

We also carefully reviewed the implementation to confirm that the code correctly computes the high-dimensional GRN. In the code, we correctly compute the expression corresponding to the last equality i.e., $\frac{\mathrm{d}\left(\left(\frac{\mathrm{d}x_l}{\mathrm{d}x_h}\right)^{-1} \cdot V_l\right)}{\mathrm{d}x_h}$, without any missing terms. Therefore, while the manuscript had a typographical omission in the intermediate equality, the underlying computational implementation remains correct and unaffected.

Minor points:

1, Fig. 4A, the color for D2 an D6 are similar, please use better color scheme.

R: Thank you for your good suggestion. We have revised the color scheme in Fig. 4A to improve distinguishability between D2 and D6. The updated figure is provided below for reference and has been incorporated into the revised manuscript.

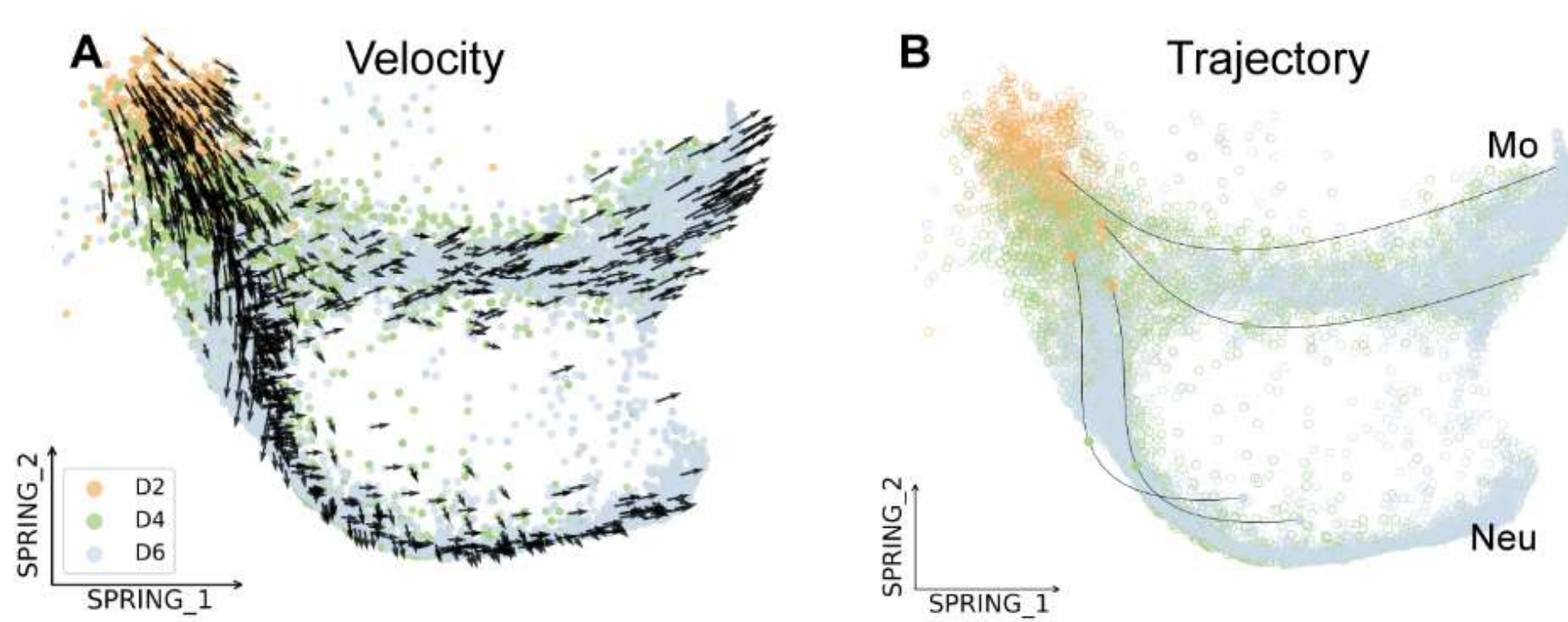

2, The terms "latent time" and "pseudotime" are used throughout the manuscript. Please ensure their definitions are clearly stated and applied consistently. Specifically, please clarify how the "latent time" inferred by GRNvelo differs conceptually from the "pseudotime" estimated by conventional methods.

R: We thank the reviewer for this suggestion. We have now explicitly defined latent time and distinguished it from conventional pseudotime in the Results section (first paragraph after introducing GRNvelo, lines 162-168). The added text is provided below for reference.

"In this study, latent time refers to the continuous temporal variable learned by GRNvelo through the TC-PINN module, which enforces consistency with experimental time points and gene regulatory ODE constraints. The latent time is normalized to [0,1] for interpretability, and it preserves an affine relationship with the true experimental time, thereby retaining absolute temporal information. This mechanistic model-based latent time distinguishes it from conventional expression similarity-based pseudotime that provides only a relative sequence of cell ordering without alignment to real time."

3, It might be useful to add a table on the computational cost of the proposed method.

R: We thank the reviewer for this suggestion. We have now added a summary of the computational cost of GRNvelo, including runtime and peak memory usage, for all datasets analyzed in the study. These results are presented in a new Supplementary Table and are also summarized in the tables provided below for reference. Additionally, we have added a note in the table to clarify that the runtime correlates with the number of cluster-level lineages and the number of iterations. The hyperparameters used in the neural networks of TC-PINN and MP-PINN are listed in Appendix Tables S5-S6 for reference.

Appendix Table S3. Computational cost of the GRNvelo model on synthetic dataset.

**Dataset**	**Cell number**	**Gene number**	**Running time**	**Memory usage**
3-gene	2,778	3	1.49 min	0.58 GB
10-gene	2,851	10	2.46 min	0.94 GB
10-gene_2	100,000	10	38.85 min	8.36 GB

Appendix Table S4. Computational cost of the GRNvelo model on real dataset.

**Dataset**	**Cell number**	**Gene number**	**Running time**	**Memory usage**
PC9 tumor cell	2,008	8437	2.05 min	1.24 GB
Lineage	15,007	2	15.75 min	1.10 GB
CD8+ T cell	50,577	16710	13.46 min	6.35 GB
Pancreas	51,274	2500	5.55 min	3.16 GB
C. elegans	89701	20222	45.54 min	6.04 GB

Response to Reviewer #3

The manuscript presents GRNvelo, an analysis tool for establishing biologically interpretable and mathematically rigorous multiscale models, which couple GRN-driven single-cell velocity with cell growth-mediated population dynamics. This is an important and formidable problem that the GRNvelo tool addresses thoroughly, with ample demonstrations with simulated and real biological data, with improved performance over existing alternatives and the ability to predict altered cell fates in response to diverse genetic perturbations. Demonstrations exist for data simulated in a 3-gene and a 10-gene network, and for real data collected from beta-cell differentiation, murine hematopoiesis, CD8+ T cell responses to infection, and lung cancer cells' drug responses.

Overall, the manuscript offers a valuable contribution and should be published, after the following comments have been addressed.

Thank you for your positive feedback and insightful comments. Now we have carefully revised the manuscript according to your constructive suggestions. We believe that we have addressed all questions you raised and that the manuscript now has been significantly improved. Below we respond to your comments point-by-point.

(1) A bit of a historical background with some citations might be useful on the experimental demonstration of the problem addressed by the tool: coupled GRN-driven single-cell dynamics and cell growth-mediated population dynamics. Probably the simplest examples emerged in synthetic biology, see PMID:22511863 and PMID: 30341217

R: Thank you for the valuable suggestion. Following your comment, we have added a brief historical background in the Introduction (lines 103-114), citing the two recommended references to highlight early synthetic biology studies that experimentally demonstrated the coupling between single-cell dynamics and population-level processes. The added text is as follows:

"The coupling between single-cell gene regulatory dynamics and population-level processes lies at the core of cell fate determination. Synthetic biology has provided direct experimental evidence for this multiscale interdependence. A bistable synthetic gene circuit in yeast established that predicting population-level fitness under drug stress requires knowledge of both single-cell division rates and the nongenetic memory of distinct expression states (Nevozhay *et al*, 2012). Subsequent work further demonstrated that nonoptimal temperatures induce a bifurcation between growth arrest and stress resistance; capturing such bimodal population behavior necessitated models integrating population dynamics with single-cell growth and reaction kinetics (Charlebois *et al*, 2018). Together, these findings highlight the necessity of a computational framework capable of bridging molecular regulatory mechanisms with population phenotypes, a capability largely absent from current approaches to predicting cell fate dynamics."

(2) GRNvelo seems to be operating on the assumption that stochasticity can be modeled

by an additive noise term. However, many studies of stochastic chemical kinetics indicate that noise can arise inherently from the stochastic nature of reactions. This means that SDEs might not be appropriate. Could Gillespie simulations of the Chemical Master Equation be tested to see how GRNvelo works with that simulated data?

R: Thank you for this good suggestion. Following the comment, we generated additional synthetic data using the Gillespie algorithm and evaluated GRNvelo on these datasets. The results, which are consistent with the SDE-based findings, have been added to the "Validation on synthetic data" section of the Results (lines 322-333) and are presented in Appendix Figure S13-S14.

"To assess whether the performance of GRNvelo depends on the specific stochastic modeling framework used for data generation, we further evaluated the method on synthetic data simulated using the Gillespie algorithm, which numerically solves the chemical master equation and captures the intrinsic stochasticity of biochemical reactions without the continuity assumption inherent in SDEs. Using the same 3-gene and 10-gene network topologies, we generated Gillespie-based time-series data and applied GRNvelo for trajectory inference. The Pearson correlation between inferred latent time and true simulation time was 0.81 for the 3-gene network and 0.93 for the 10-gene network (Appendix Figure S6-S7), comparable to the results obtained on SDE-generated data (0.81 and 0.97, respectively). These results demonstrate that GRNvelo maintains robust inference performance across distinct stochastic modeling frameworks, supporting its applicability to biological systems where gene expression noise arises from discrete molecular events."

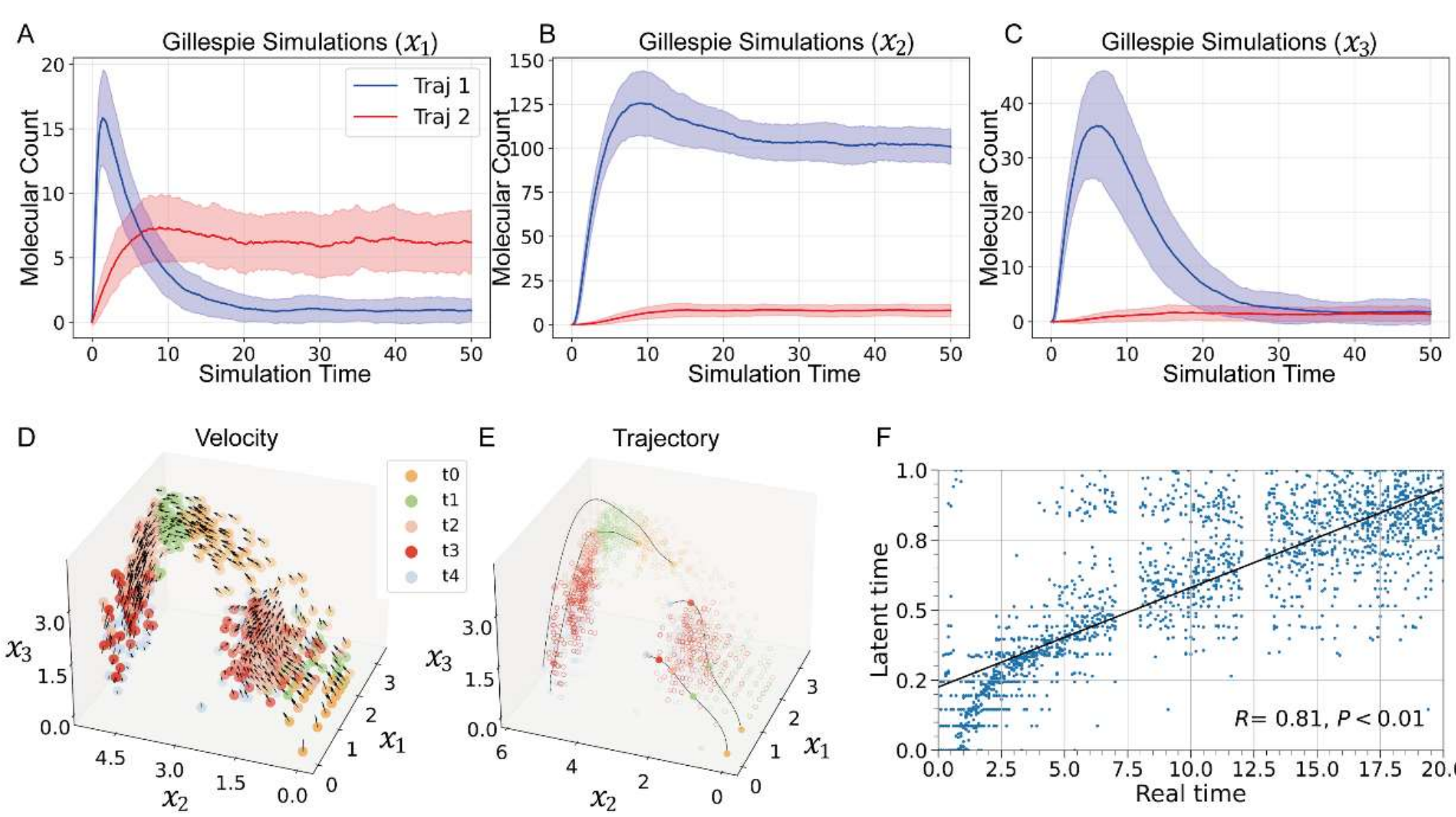

Appendix Figure S6. Gillespie-based simulation and model validation of a 3-gene regulatory network. (**A-C**) Two representative trajectory groups (mean ± SD) for gene (**A**)x_1, (**B**)x_2, (**C**)x_3 from Gillespie simulations. (**D**) Predicted cell velocities. (**E**) Predicted cell trajectories. (**F**) Pearson correlation between predicted latent time and true simulation time.

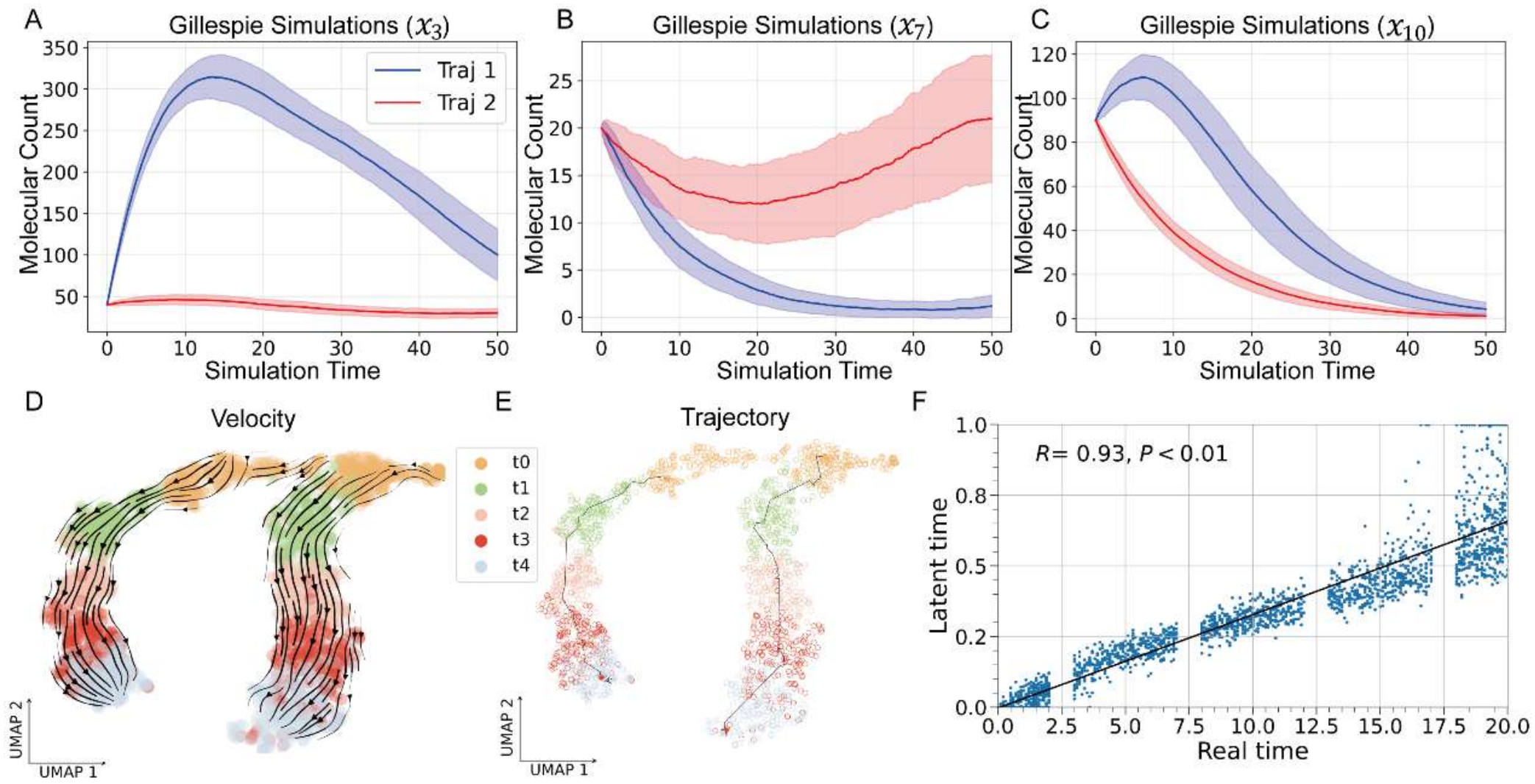

Appendix Figure S7. Gillespie−based simulation and model validation of a 10-gene regulatory network. (**A-C**) Two representative trajectory groups (mean ± SD) for gene (**A**) x_3 , (**B**) x_7 , (**C**) x_{10} , which shows large expression differences between the two trajectories. (**D**) Predicted cell velocities. (**E**) Predicted cell trajectories. (**F**) Pearson correlation between predicted latent time and true simulation time.

(3) Adding the simplest possible simulations to generate data for GRNvelo based on very simple synthetic biological systems, like a simple positive autoregulation (including with Gillespie simulations) could be interesting - in addition, before the 3-node and 10-node networks.

R: We thank the reviewer for this suggestion. Following the comment, we performed Gillespie simulations of a single-gene positive autoregulation system and validated GRNvelo on this dataset. The results have been added to the beginning of the “Validation on synthetic data” section of the Results (lines 214-225) and are presented as Appendix Figure S12. The original opening sentence of this section has been revised accordingly to maintain logical flow from the simplest motif to more complex networks.

“To evaluate GRNvelo on the simplest regulatory motif, we first simulated a single-gene positive autoregulation system using the Gillespie algorithm (Gillespie, 1977; Matthew *et al*, 2023), which captures the intrinsic stochasticity of biochemical reactions (Appendix Figure S1A). From the simulated trajectories, we sampled cells at five discrete time points for model training and validation (Appendix Figure S1B). GRNvelo successfully reconstructed the gene expression dynamics (Appendix Figure S1C) and accurately predicted cellular trajectories (Appendix Figure S1D), demonstrating its capability to infer cell fate dynamics from data generated under a fundamentally different stochastic modeling framework. Building on this validation, we next evaluated GRNvelo on more complex synthetic networks.

To further evaluate GRNvelo on more complex regulatory networks, we next conducted systematic evaluations using two synthetic datasets generated from networks of different scales (3-gene and 10-gene systems) (Fig. 2).”

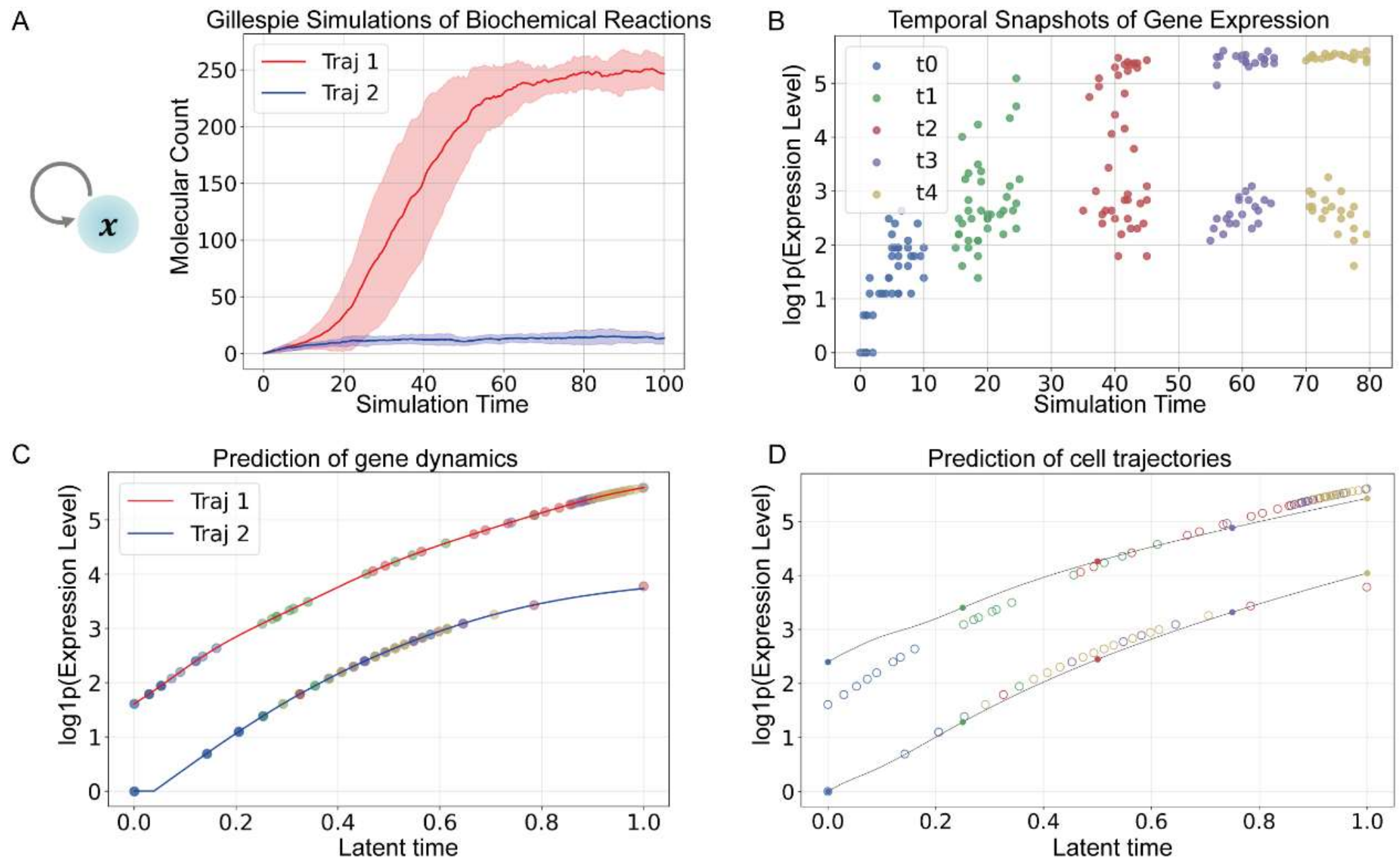

Appendix Figure S1. Gillespie-based simulation and model validation of a positive autoregulation system. (**A**) Topology of a single-gene autoregulatory circuit and two representative trajectory groups (mean ± SD) from Gillespie simulations. (**B**) Selected cells for model training and validation, annotated with five discrete time points. (**C**) Visualization of gene dynamics predicted by the model. (**D**) Predicted cell trajectories.

(4) What would experimental biologists do differently after GRNvelo is published? What new experiments would GRNvelo suggest that would not have been conceived without GRNvelo? This question should be answered specifically for the data that was analyzed as examples, and in general, for other data that will be analyzed.

R: Thank you for this constructive comment. Following the suggestion, we have added a paragraph in the Discussion section (lines 684-690) discussing the practical implications of GRNvelo for experimental biology.

"Beyond trajectory inference, GRNvelo offers predictive capabilities for experimental design. It enables biologists to computationally test perturbation hypotheses, predict responses to multi-gene or dynamic interventions, and optimize sampling intervals for time-series experiments. For the datasets analyzed here, GRNvelo identifies key regulators in β-cell differentiation, reveals in CD8+ T cells that higher proliferation rates correlate with a preferential commitment to effector fate over memory fate, and predicts effective drug combinations in resistant tumors, providing testable hypotheses for future investigations."

(5) The phrase "phenotypic evolution dynamics" or "cell state evolution" might create some confusion. Here it means time-evolution in the mathematical sense. However, in the biology community, this means the phenotypic manifestation of some biological evolutionary process. It would be better to use "phenotypic time course" or "phenotypic dynamics" or

something else to avoid connotations of biological (such as Darwinian) evolution.

R: Thank you for this good comment. Following your suggestion, we have uniformly replaced the potentially confusing phrases (e.g., “phenotypic evolution dynamics”, “cell state evolution”) with “phenotypic dynamics” throughout the manuscript (e.g., lines 135, 162, 843) to avoid unintended connotations of Darwinian evolution.

(6) It is actually interesting to ask, can GRNvelo be developed towards predicting longer-term evolutionary processes in the biological sense? If cellular growth rates differ, that means there is evolutionary pressure.

R: We thank the reviewer for this forward-looking question. Following the suggestion, we have added a paragraph in the Discussion section (lines 739-747) discussing the potential extension of GRNvelo to model long-term evolutionary processes, noting that the framework already incorporates growth rate differences and GRN-driven transitions as key ingredients, and outlining future directions for incorporating mutation accumulation and population bottlenecks.

“Beyond the developmental and disease-relevant timescales considered here, GRNvelo also holds potential for modeling longer-term evolutionary processes. The framework already captures two key drivers of evolution: growth rate differences among cell states, which generate selective pressure, and GRN-driven phenotypic transitions, which provide heritable variation in the broad sense. Extending GRNvelo to model Darwinian evolution would require incorporating additional components such as mutation accumulation, strict genetic heritability, and population bottlenecks. Such an extension could enable prediction of clonal selection dynamics in cancer progression, adaptive resistance to therapy, and microbial evolution under environmental stress. We envision this as a promising direction for future research.”

(7) Some of the fonts are too small in multiple figures (axis and bar labels, colorbar marks, etc.)

R: Thank you for pointing this out. We have carefully reviewed all figures and increased the font sizes for axis labels, bar labels, colorbar marks, and other text elements to ensure readability. Individual high-resolution figures have been uploaded into the submission system.

(8) The code is not accessible. https://github.com/SunXQlab/%20GRNvelo is giving an error.

R: Thank you for bringing this to our attention. The previous error may be due to a mistake of an unexpected space in the provided URL (i.e., “%20”) in the manuscript text. The code repository is now publicly accessible at the URL: https://github.com/SunXQlab/GRNvelo, which has been updated in the revised manuscript. All relevant code, including the main GRNvelo implementation, example scripts, and pre-processed datasets, has been uploaded and is ready for review. We appreciate the reviewer’s patience.

(9) The code should be friendly and accessible for interested biologists to be able to try

and even modify for processing other data. Consider developing a GUI - or at least propose so for the future.

R: Thank you for this important suggestion. We agree that making the code accessible and user-friendly is essential for enabling experimental biologists to adopt GRNvelo. In the revised code repository, we have added detailed documentation and a step-by-step example demonstrating how to run GRNvelo on a sample dataset. This example includes instructions for data preprocessing, parameter settings, and interpretation of outputs, allowing interested biologists to easily adapt the workflow to their own data.

We hope these improvements make GRNvelo more readily usable by the broader biology community.

References

Charlebois DA, Hauser K, Marshall S, Balázsi G (2018) Multiscale effects of heating and cooling on genes and gene networks. *Proceedings of the National Academy of Sciences of the United States of America* 115: E10797-E10806

Gillespie DT (1977) Exact stochastic simulation of coupled chemical reactions. *The Journal of Physical Chemistry* 81: 2340-2361

Kang X, Li C (2021) A Dimension Reduction Approach for Energy Landscape: Identifying Intermediate States in Metabolism-EMT Network. *Advanced Science* 8: 2003133

Matthew S, Carter F, Cooper J, Dippel M, Green E, Hodges S, Kidwell M, Nickerson D, Rumsey B, Reeve J *et al* (2023) GillesPy2: A Biochemical Modeling Framework for Simulation Driven Biological Discovery. *Letters in biomathematics* 10: 87-103

Nevozhay D, Adams RM, Van Itallie E, Bennett MR, Balázsi G (2012) Mapping the Environmental Fitness Landscape of a Synthetic Gene Circuit. *PLOS Computational Biology* 8: e1002480

Packer JS, Zhu Q, Huynh C, Sivaramakrishnan P, Preston E, Dueck H, Stefanik D, Tan K, Trapnell C, Kim J *et al* (2019) A lineage-resolved molecular atlas of C. elegans embryogenesis at single-cell resolution. 365: eaax1971

Riba A, Oravecz A, Durik M, Jiménez S, Alunni V, Cerciat M, Jung M, Keime C, Keyes WM, Molina N (2022) Cell cycle gene regulation dynamics revealed by RNA velocity and deep-learning. *Nature Communications* 13: 2865

Schwabe D, Formichetti S, Junker JP, Falcke M, Rajewsky N (2020) The transcriptome dynamics of single cells during the cell cycle. *Molecular Systems Biology* 16: MSB20209946

Sha Y, Qiu Y, Zhou P, Nie Q (2024) Reconstructing growth and dynamic trajectories from single-cell transcriptomics data. *Nature Machine Intelligence* 6: 25-39

Somer J, Mannor S, Alon U (2026) Temporal tissue dynamics from a spatial snapshot. *Nature* 650: 490-499

Ullrich KK. celegans scRNA data set objects (0.0.2) (2022) (https://doi.org/10.5281/zenodo.7496490) [DATASET]

Yan L, Cheng J, Nie Q, Sun X (2025) Dissecting multilayer cell-cell communications with

signaling feedback loops from spatial transcriptomics data. *Genome research* 35: 1400-1414

Yan L, Zhang D, Sun X (2026) Decoding cell state transitions driven by dynamic cell–cell communication in spatial transcriptomics. *Nature Computational Science* 6: 193-207

Yang W, Wang P, Xu S, Wang T, Luo M, Cai Y, Xu C, Xue G, Que J, Ding Q *et al* (2024) Deciphering cell–cell communication at single-cell resolution for spatial transcriptomics with subgraph-based graph attention network. *Nature Communications* 15: 7101

Ying Z, Xin Y, Liu Z, Tan T, Huang Y, Ding Y, Hong X, Li Q, Li C, Guo J *et al* (2025) The mitochondrial unfolded protein response inhibits pluripotency acquisition and mesenchymal-to-epithelial transition in somatic cell reprogramming. *Nature Metabolism* 7: 940-951

Zhang R, Lahens NF, Ballance HI, Hughes ME, Hogenesch JB (2014) A circadian gene expression atlas in mammals: Implications for biology and medicine. 111: 16219-16224

Zhu L, Wang J (2024) Quantifying Landscape-Flux via Single-Cell Transcriptomics Uncovers the Underlying Mechanism of Cell Cycle. *Advanced science (Weinheim, Baden-Wurttemberg, Germany)* 11: e2308879

Zhu L, Yang S, Zhang K, Wang H, Fang X, Wang J (2024) Uncovering underlying physical principles and driving forces of cell differentiation and reprogramming from single-cell transcriptomics. *Proceedings of the National Academy of Sciences* 121: e2401540121

4th May 2026

Manuscript Number: MSB-2026-13577R
Title: Multiscale learning of gene network-driven phenotypic dynamics of single cells

Dear Prof Sun,

Thank you for the submission of your revised manuscript to Molecular Systems Biology. We have now received the enclosed reports from the referees that were asked to re-assess it. As you will see the reviewers are now globally supportive and I am pleased to inform you that we will be able to accept your manuscript pending the following final amendments:

1) Please format the Data availability section according to the example below. You may remove the statements that 'This study includes no data deposited in external repositories', and those regarding the Source data and Expanded view data, etc. These will be added in by Production when necessary. The format of the statement should be as follows:
"The datasets and computer code produced in this study are available in the following databases:
- Chip-Seq data: Gene Expression Omnibus GSE46748 (https://www.ncbi.nlm.nih.gov/geo/query/acc.cgi?acc=GSE46748)
- Modeling computer scripts: GitHub (https://github.com/SysBioChalmers/GECKO/releases/tag/v1.0)
- [data type]: [full name of the resource] [accession number/identifier] ([doi or URL or identifiers.org/DATABASE:ACCESSION])"
2) Please ensure that the README file on Github has sufficient practical use instructions for potential future users of your code.
3) Please rename "Competing interest statement" to "Disclosure and competing interests statement". We updated our journal's competing interests policy in January 2022 and request authors to consider both actual and perceived competing interests. Please review the policy https://link.springer.com/partners/embo-press/editorial-policies#Competing%20interest%20disclosures and update your competing interests if necessary.
4) Please remove the authors contributions from the manuscript (it is sufficient that you already provided the author contributions in our submission system).
5) In the Methods, please take care of the following:
- The Materials and Methods section should be renamed to "Methods".
6) Please place individual sections of the manuscript in the following order: Title page - Abstract & Keywords - Introduction - Results - Discussion - Methods - Data Availability - Acknowledgements - Disclosure and Competing Interests Statement - References - Figure Legends - (Main Tables with legends if applicable) - Expanded View Figure Legends.
7) For the figures and figure legends, please take care of the following:
- Please make sure to update the callouts of all figures in the main manuscript text (currently figure callouts are missing for Figure 1A, C-F).
- Please note that the exact p values are not provided in the legends of figures 2H, P
8) In the Appendix file, please remove the author list and affiliations from the cover page, and add "Appendix for [manuscript title]" and a Table of Contents with each item listed (figures, tables) with its page number. Please therefore remove the 'Supplementary information' section from the main manuscript.
9) Source Data should be organized as a single source data file (zipped) per figure for main figures (all EV and/or Appendix figure Source Data can be included in a single folder), with the panels clearly visible in the folder structure instead of a single excel file for all Source Data. e.g. all the Source data files for figure 1 need to be saved in a single folder and this needs to be zipped and then uploaded as "SD figure 1.zip" file. Please also update the Source Data checklist, as you have indicated Source Data should be provided for Figure 1, 2A as macroscopic photos - this does not seem to match the main figures provided, i.e. it does not appear that source data are necessary for these figures.
10) As part of the EMBO Publications transparent editorial process initiative (see our policy here: https://www.embopress.org/transparent-process#Review_Process), Molecular Systems Biology will publish online a Peer Review File (PRF) to accompany accepted manuscripts. This file will be published in conjunction with your paper and will include the anonymous referee reports, your point-by-point response and all pertinent correspondence relating to the manuscript. Let us know whether you agree with the publication of the PRF and as here, if you want to remove or not any figures from it prior to publication. Please note that the Authors checklist will be published at the end of the PRF.
11) After your paper is published, we may promote it on social media. If you have any handles or hashtags for Bluesky you would like included, please let us know.
12) Please provide a point-by-point letter INCLUDING my comments and your detailed responses (as Word file).

I look forward to reading a new revised version of your manuscript as soon as possible.

Yours sincerely,

Poonam Bheda, PhD
Senior Scientific Editor
Molecular Systems Biology

Reviewer #1:

The authors have addressed my concerns. Recommend for publication.

Reviewer #2:

I appreciate the authors' efforts to address all of my concerns. I recommend the publication of this paper.

Reviewer #3:

I would like to thank the authors for thoroughly addressing my comments. I would like to recommend the revised manuscript for publication.

Response to Editor

Thank you for your positive decision and the detailed guidance on the final amendments. We have carefully addressed all the comments point by point. Below is our detailed response.

1) Please format the Data availability section according to the example below. You may remove the statements that 'This study includes no data deposited in external repositories', and those regarding the Source data and Expanded view data, etc. These will be added in by Production when necessary. The format of the statement should be as follows:
"The datasets and computer code produced in this study are available in the following databases:
- Chip-Seq data: Gene Expression Omnibus GSE46748 (https://www.ncbi.nlm.nih.gov/geo/query/acc.cgi?acc=GSE46748)
- Modeling computer scripts: GitHub (https://github.com/SysBioChalmers/GECKO/releases/tag/v1.0)
- [data type]: [full name of the resource] [accession number/identifier] ([doi or URL or identifiers.org/DATABASE:ACCESSION])"

R: We have reformatted the Data availability section exactly as requested. The previous statements regarding "no external data" and source data have been removed. The revised section now reads (on Lines 1296-1298 of the manuscript):
"The datasets and computer code produced in this study are available in the following databases:
Modeling computer scripts: *GitHub (https://github.com/SunXQlab/GRNvelo).*"

2) Please ensure that the README file on Github has sufficient practical use instructions for potential future users of your code.

R: We have substantially updated the README file on our GitHub repository (https://github.com/SunXQlab/GRNvelo). Specifically, we added detailed file-by-file comments for all scripts in the Model folder to help future users understand the purpose and usage of each component. The added comments are as follows:
"model/AE – Reduces the dimensionality of high-dimensional data;

- model/TFvelo – The package used for drawing velocity fields and calculating pseudotime;
- model/Train_Generation.py – Combines three standalone codes (cell density estimation after dimensionality reduction, TC‑PINN, and MP‑PINN) into a single training script. Running this script trains all three components together in one unified process;
- model/DensityGeneration.py – Code for calculating the density of the data after dimensionality reduction;
- model/VelocityGeneration.py – Implements the TC‑PINN (Temporally-Consistent

Physics‐Informed Neural Network), which learns cellular velocity fields from single‐cell data;

- model/VelocityRefine.py – Implements the MP‐PINN (Multi-Physics Informed Neural Network), which refines the raw velocity fields generated by TC‐PINN, enforcing physical constraints such as mass conservation or divergence‐free flow. This module is trained jointly with TC‐PINN and density estimation in the unified pipeline."

These annotations, together with other usage instructions already present, ensure that potential users can readily understand and run our code. The updated README has been committed and is publicly visible.

3) Please rename "Competing interest statement" to "Disclosure and competing interests statement". We updated our journal's competing interests policy in January 2022 and request authors to consider both actual and perceived competing interests. Please review the policy https://link.springer.com/partners/embo-press/editorial-policies#Competing%20interest%20disclosures and update your competing interests if necessary.

R: We have renamed the heading from "Competing interest statement" to "Disclosure and competing interests statement" as requested. The updated heading appears on Line 1304 of the manuscript. We have reviewed the journal's updated competing interests policy (January 2022) and confirm that our disclosure remains accurate and complete; no additional competing interests need to be declared.

4) Please remove the authors contributions from the manuscript (it is sufficient that you already provided the author contributions in our submission system).

R: We have removed the "Author contributions" section entirely from the manuscript, as requested. The author contributions have already been provided in the submission system.

5) In the Methods, please take care of the following:
- The Materials and Methods section should be renamed to "Methods".

R: We have renamed the section heading from "Materials and Methods" to "Methods" as requested. The updated heading appears on Line 750 of the manuscript.

6) Please place individual sections of the manuscript in the following order: Title page - Abstract & Keywords - Introduction - Results - Discussion - Methods - Data Availability - Acknowledgements - Disclosure and Competing Interests Statement - References - Figure Legends - (Main Tables with legends if applicable) - Expanded View Figure Legends.

R: We have reorganized the manuscript sections according to the required order. The sections now appear sequentially as: Title page, Abstract & Keywords, Introduction, Results, Discussion, Methods, Data Availability, Acknowledgements, Disclosure and Competing Interests Statement, References, and Figure Legends. We note that the items "Main Tables with legends (if applicable)" and "Expanded View Figure Legends" do not apply to our manuscript.

7) For the figures and figure legends, please take care of the following:
- Please make sure to update the callouts of all figures in the main manuscript text (currently figure callouts are missing for Figure 1A, C-F).

R: We have added the missing figure callouts for Figure 1 in the main text. The specific locations are as follows:
- Figure 1A – Line 147
- Figure 1B – Line 148
- Figure 1C – Lines 170–171
- Figure 1D – Line 149
- Figure 1E – Line 174
- Figure 1F – Line 174

All missing callouts have now been included.

- Please note that the exact p values are not provided in the legends of figures 2H, P

R: We have updated the figure legends for Figures 2H and 2P. The revised legends (on Lines 1528 and 1532) now include the exact p-value ($p < 0.0001$), along with the Pearson's correlation coefficient and sample size, as shown below:
"(H) correlation between latent time and real time. Pearson's correlation, r = 0.81, n = 2778, $p < 0.0001$.
(P) correlation between latent time and real time. Pearson's correlation, r = 0.97, n = 2851, $p < 0.0001$."

8) In the Appendix file, please remove the author list and affiliations from the cover page, and add "Appendix for [manuscript title]" and a Table of Contents with each item listed (figures, tables) with its page number. Please therefore remove the 'Supplementary information' section from the main manuscript.

R: We have modified the Appendix file as requested. Specifically:
- The author list and affiliations have been removed from the cover page;
- The cover page now includes the title "Appendix for [manuscript title]" and a Table of Contents listing all items (figures and tables) with their corresponding page numbers;
- The "Supplementary information" section has been removed from the main manuscript.

All requested changes to the Appendix and main manuscript have been completed.

9) Source Data should be organized as a single source data file (zipped) per figure for main figures (all EV and/or Appendix figure Source Data can be included in a single folder), with the panels clearly visible in the folder structure instead of a single excel file for all Source Data. e.g. all the Source data files for figure 1 need to be saved in a single folder and this needs to be zipped and then uploaded as "SD figure 1.zip" file. Please also update the Source Data checklist, as you have indicated Source Data should be provided for Figure 1, 2A as macroscopic photos - this does not seem to match the main figures provided, i.e. it does not appear that source data are necessary for these figures.

R: We have reorganized the Source Data as requested. Each main figure now has its own zipped source data file (e.g., SD Figure 2.zip), with clear folder structure per panel. The Source Data checklist has also been updated accordingly.

10) As part of the EMBO Publications transparent editorial process initiative (see our policy here: https://www.embopress.org/transparent-process#Review_Process), Molecular Systems Biology will publish online a Peer Review File (PRF) to accompany accepted manuscripts. This file will be published in conjunction with your paper and will include the anonymous referee reports, your point-by-point response and all pertinent correspondence relating to the manuscript. Let us know whether you agree with the publication of the PRF and as here, if you want to remove or not any figures from it prior to publication. Please note that the Authors checklist will be published at the end of the PRF.

R: We agree to the publication of the Peer Review File accompanying our manuscript. We have no figures or other content to remove from the PRF.

11) After your paper is published, we may promote it on social media. If you have any handles or hashtags for Bluesky you would like included, please let us know.

R: We do not have any Bluesky handles or hashtags to include.

12) Please provide a point-by-point letter INCLUDING my comments and your detailed responses (as Word file).

R: This document serves as our point-by-point response letter, including all editorial comments and our detailed responses as requested. We provide this letter as a Word file upon submission.

19th May 2026
Manuscript number: MSB-2026-13577RR
Title: Multiscale learning of gene network-driven phenotypic dynamics of single cells

Dear Prof Sun,

Thank you again for sending us your revised manuscript. We are now satisfied with the modifications made and I am pleased to inform you that your paper has been accepted for publication.

Your manuscript will be processed for publication by EMBO Press. It will be copy edited and you will receive page proofs prior to publication. Please note that you will be contacted by Springer Nature Author Services to complete licensing and payment information.

You may qualify for financial assistance for your publication charges - either via a Springer Nature fully open access agreement or an EMBO initiative. Check your eligibility: https://link.springer.com/journal/44320/how-to-publish-with-us

Should you be planning a Press Release on your article, please get in contact with embo_production@springernature.com as early as possible in order to coordinate publication and release dates.

If you have any questions, please do not hesitate to contact the Editorial Office. Thank you for your contribution to Molecular Systems Biology.

Yours sincerely,

Poonam Bheda, PhD
Scientific Editor
Molecular Systems Biology

>>> Please note that it is Molecular Systems Biology policy for the transcript of the editorial process (containing referee reports and your response letter) to be published as an online supplement to each paper. If you do NOT want this, you will need to inform the Editorial Office via email immediately. More information is available here: https://link.springer.com/partners/embo-press/editorial-policies#Peer%20review

EMBO Press Author Checklist

Corresponding Author Name: Xiaoqiang Sun	
Journal Submitted to: Molecular Systems Biology	
Manuscript Number: MSB-2026-13577	

USEFUL LINKS FOR COMPLETING THIS FORM
The EMBO Journal - Author Guidelines
EMBO Reports - Author Guidelines
Molecular Systems Biology - Author Guidelines
EMBO Molecular Medicine - Author Guidelines

Reporting Checklist for Life Science Articles (updated January
This checklist is adapted from Materials Design Analysis Reporting (MDAR) Checklist for Authors. MDAR establishes a minimum set of requirements in transparent reporting in the life sciences (see Statement of Task: 10.31222/osf.io/9sm4x). Please follow the journal's guidelines in preparing your
Please note that a copy of this checklist will be published alongside your article.

Abridged guidelines for figures
1. Data
The data shown in figures should satisfy the following conditions:
- the data were obtained and processed according to the field's best practice and are presented to reflect the results of the experiments in an accurate and unbiased manner.
- ideally, figure panels should include only measurements that are directly comparable to each other and obtained with the same assay.
- plots include clearly labeled error bars for independent experiments and sample sizes. Unless justified, error bars should not be shown for technical
- if n<5, the individual data points from each experiment should be plotted. Any statistical test employed should be justified.
- Source Data should be included to report the data underlying figures according to the guidelines set out in the authorship guidelines on Data

2. Captions
Each figure caption should contain the following information, for each panel where they are relevant:
- a specification of the experimental system investigated (eg cell line, species name).
- the assay(s) and method(s) used to carry out the reported observations and measurements.
- an explicit mention of the biological and chemical entity(ies) that are being measured.
- an explicit mention of the biological and chemical entity(ies) that are altered/varied/perturbed in a controlled manner.
- the exact sample size (n) for each experimental group/condition, given as a number, not a range;
- a description of the sample collection allowing the reader to understand whether the samples represent technical or biological replicates (including how many animals, litters, cultures, etc.).
- a statement of how many times the experiment shown was independently replicated in the laboratory.
- definitions of statistical methods and measures:
  - common tests, such as t-test (please specify whether paired vs. unpaired), simple χ2 tests, Wilcoxon and Mann-Whitney tests, can be unambiguously identified by name only, but more complex techniques should be described in the methods section;
  - are tests one-sided or two-sided?
  - are there adjustments for multiple comparisons?
  - exact statistical test results, e.g., P values = x but not P values < x;
  - definition of 'center values' as median or average;
  - definition of error bars as s.d. or s.e.m.

Please complete ALL of the questions below.
Select "Not Applicable" only when the requested information is not relevant for your study.

Materials

Newly Created Materials	Information included in the manuscript?	In which section is the information available? (Reagents and Tools Table, Materials and Methods, Figures, Data Availability Section)
New materials and reagents need to be available; do any restrictions apply?	Not Applicable	

Antibodies	Information included in the manuscript?	In which section is the information available? (Reagents and Tools Table, Materials and Methods, Figures, Data Availability Section)
For **antibodies** provide the following information: - Commercial antibodies: RRID (if possible) or supplier name, catalogue number and or/clone number - Non-commercial: RRID or citation	Not Applicable	

DNA and RNA sequences	Information included in the manuscript?	In which section is the information available? (Reagents and Tools Table, Materials and Methods, Figures, Data Availability Section)
**Short novel DNA or RNA including primers, probes**: provide the sequences.	Not Applicable	

Cell materials	Information included in the manuscript?	In which section is the information available? (Reagents and Tools Table, Materials and Methods, Figures, Data Availability Section)
**Cell lines:** Provide species information, strain. Provide accession number in repository **OR** supplier name, catalog number, clone number, and/**OR** RRID.	Not Applicable	
**Primary cultures:** Provide species, strain, sex of origin, genetic modification status.	Not Applicable	
Report if the cell lines were recently **authenticated** (e.g., by STR profiling) and tested for mycoplasma contamination.	Not Applicable	

Experimental animals	Information included in the manuscript?	In which section is the information available? (Reagents and Tools Table, Materials and Methods, Figures, Data Availability Section)
**Laboratory animals or Model organisms:** Provide species, strain, sex, age, genetic modification status. Provide accession number in repository **OR** supplier name, catalog number, clone number, **OR** RRID.	Not Applicable	
**Animal observed in or captured from the field:** Provide species, sex, and age where possible.	Not Applicable	
Please detail **housing and husbandry conditions**.	Not Applicable	

Plants and microbes	Information included in the manuscript?	In which section is the information available? (Reagents and Tools Table, Materials and Methods, Figures, Data Availability Section)
**Plants:** provide species and strain, ecotype and cultivar where relevant, unique accession number if available, and source (including location for collected wild specimens).	Not Applicable	
**Microbes:** provide species and strain, unique accession number if available, and source.	Not Applicable	

Human research participants	Information included in the manuscript?	In which section is the information available? (Reagents and Tools Table, Materials and Methods, Figures, Data Availability Section)
If collected and within the bounds of privacy constraints report on age, sex and gender or ethnicity for all study participants.	Not Applicable	

Core facilities	Information included in the manuscript?	In which section is the information available? (Reagents and Tools Table, Materials and Methods, Figures, Data Availability Section)
If your work benefited from core facilities, was their service mentioned in the acknowledgments section?	Not Applicable	

Design

Study protocol	Information included in the manuscript?	In which section is the information available? (Reagents and Tools Table, Materials and Methods, Figures, Data Availability Section)
If study protocol has been **pre-registered, provide DOI in the manuscript**. For clinical trials, provide the trial registration number **OR** cite DOI.	Not Applicable	
Report the **clinical trial registration number** (at ClinicalTrials.gov or equivalent), where applicable.	Not Applicable	

Laboratory protocol	Information included in the manuscript?	In which section is the information available? (Reagents and Tools Table, Materials and Methods, Figures, Data Availability Section)
Provide DOI OR other citation details if **external detailed step-by-step protocols** are available.	Not Applicable	

Experimental study design and statistics	Information included in the manuscript?	In which section is the information available? (Reagents and Tools Table, Materials and Methods, Figures, Data Availability Section)
Include a statement about **sample size** estimate even if no statistical methods were used.	Not Applicable	
Were any steps taken to minimize the effects of subjective bias when allocating animals/samples to treatment (e.g. **randomization procedure**)? If yes, have they been described?	Not Applicable	
Include a statement about **blinding** even if no blinding was done.	Not Applicable	
Describe **inclusion/exclusion criteria** if samples or animals were excluded from the analysis. Were the criteria pre-established?<br><br>If sample or data points were omitted from analysis, report if this was due to attrition or intentional exclusion and provide justification.	Not Applicable	
For every figure, are **statistical tests** justified as appropriate? Do the data meet the assumptions of the tests (e.g., normal distribution)? Describe any methods used to assess it. Is there an estimate of variation within each group of data? Is the variance similar between the groups that are being statistically compared?	Not Applicable	

Sample definition and in-laboratory replication	Information included in the manuscript?	In which section is the information available? (Reagents and Tools Table, Materials and Methods, Figures, Data Availability Section)
In the figure legends: state number of times the experiment was **replicated** in laboratory.	Not Applicable	
In the figure legends: define whether data describe **technical or biological replicates**.	Not Applicable	

Ethics

Ethics	Information included in the manuscript?	In which section is the information available? (Reagents and Tools Table, Materials and Methods, Figures, Data Availability Section)
Studies involving **human participants**: State details of **authority granting ethics approval** (IRB or equivalent committee(s), provide reference number for approval.	Not Applicable	
Studies involving **human participants**: Include a statement confirming that **informed consent** was obtained from all subjects and that the experiments conformed to the principles set out in the WMA Declaration of Helsinki and the Department of Health and Human Services Belmont Report.	Not Applicable	
Studies involving **human participants:** For publication of **patient photos**, include a statement confirming that consent to publish was obtained.	Not Applicable	
Studies involving experimental **animals**: State details of **authority granting ethics approval** (IRB or equivalent committee(s), provide reference number for approval. Include a statement of compliance with ethical regulations.	Not Applicable	
Studies involving **specimen and field samples:** State if relevant **permits** obtained, provide details of authority approving study; if none were required, explain why.	Not Applicable	

Dual Use Research of Concern (DURC)	Information included in the manuscript?	In which section is the information available? (Reagents and Tools Table, Materials and Methods, Figures, Data Availability Section)
Could your study fall under dual use research restrictions? Please check biosecurity documents and list of **select agents and toxins** (CDC): https://www.selectagents.gov/sat/list.htm	Not Applicable	
If you used a select agent, is the security level of the lab appropriate and reported in the manuscript?	Not Applicable	
If a study is subject to dual use research of concern regulations, is the name of the **authority granting approval and reference number** for the regulatory approval provided in the manuscript?	Not Applicable	

Reporting

The MDAR framework recommends adoption of discipline-specific guidelines, established and endorsed through community initiatives. Journals have their own policy about requiring specific guidelines and recommendations to complement MDAR.

Adherence to community standards	Information included in the manuscript?	In which section is the information available? (Reagents and Tools Table, Materials and Methods, Figures, Data Availability Section)
State if relevant guidelines or checklists (e.g., **ICMJE, MIBBI, ARRIVE, PRISMA**) have been followed or provided.	Not Applicable	
For **tumor marker prognostic studies,** we recommend that you follow the **REMARK** reporting guidelines (see link list at top right). See author guidelines, under 'Reporting Guidelines'. Please confirm you have followed these guidelines.	Not Applicable	
For **phase II and III randomized controlled trials**, please refer to the **CONSORT** flow diagram (see link list at top right) and submit the CONSORT checklist (see link list at top right) with your submission. See author guidelines, under 'Reporting Guidelines'. Please confirm you have submitted this list.	Not Applicable	

Data Availability

Data availability	Information included in the manuscript?	In which section is the information available? (Reagents and Tools Table, Materials and Methods, Figures, Data Availability Section)
Have **primary datasets** been deposited according to the journal's guidelines (see 'Data Deposition' section) and the respective accession numbers provided in the Data Availability Section?	Not Applicable	
Were **human clinical and genomic datasets** deposited in a public access-controlled repository in accordance to ethical obligations to the patients and to the applicable consent agreement?	Not Applicable	
Are **computational models** that are central and integral to a study available without restrictions in a machine-readable form? Were the relevant accession numbers or links provided?	Yes	Data Availability
If publicly available data were reused, provide the respective **data citations in the reference list**.	Yes	Materials and Methods
